# Molecular and Morphological Species Boundaries in the Gorgonian Octocoral Genus *Pterogorgia* (Octocorallia: Gorgoniidae)

**DOI:** 10.1371/journal.pone.0133517

**Published:** 2015-07-21

**Authors:** Herman H. Wirshing, Andrew C. Baker

**Affiliations:** Department of Marine Biology and Ecology, Rosenstiel School of Marine and Atmospheric Science, University of Miami, Miami, Florida, United States of America; Tel-Aviv University, ISRAEL

## Abstract

Most gorgonian octocoral species are described using diagnostic characteristics of their sclerites (microscopic skeletal components). Species in the genus *Pterogorgia*, however, are separated primarily by differences in their calyx and branch morphology. Specimens of a morphologically unusual *Pterogorgia* collected from Saba Bank in the NE Caribbean Sea were found with calyx morphology similar to *P*. *citrina* and branch morphology similar to *P*. *guadalupensis*. In order to test morphological species boundaries, and the validity of calyx and branch morphology as systematic characters, a phylogenetic analysis was undertaken utilizing partial gene fragments of three mitochondrial (*mtMutS*, cytochrome *b*, and *igr*4; 726bp total) and two nuclear (*ITS2*, 166bp; and *SRP54* intron, 143bp) loci. The datasets for nuclear and mitochondrial loci contained few phylogenetically informative sites, and tree topologies did not resolve any of the morphological species as monophyletic groups. Instead, the mitochondrial loci and *SRP54* each recovered two clades but were slightly incongruent, with a few individuals of *P*. *guadalupensis* represented in both clades with *SRP54*. A concatenated dataset of these loci grouped all *P*. *anceps* and *P*. *guadalupensis* in a clade, and *P*. *citrina* and the *Pterogorgia* sp. from Saba Bank in a sister clade, but with minimal variation/resolution within each clade. However, in common with other octocoral taxa, the limited genetic variation may not have been able to resolve whether branch variation represents intraspecific variation or separate species. Therefore, these results suggest that there are at least two phylogenetic lineages of *Pterogorgia* at the species level, and the atypical *Pterogorgia* sp. may represent an unusual morphotype of *P*. *citrina*, possibly endemic to Saba Bank. Branch morphology does not appear to be a reliable morphological character to differentiate *Pterogorgia* species (e.g., branches “flat” or “3–4 edges” in *P*. *guadalupensis* and *P*. *anceps*, respectively), and a re-evaluation of species-level characters (e.g., sclerites) is needed.

## Introduction

Gorgonian octocorals comprise an informal grouping of taxonomically diverse marine organisms nested within the octocorallian order Alcyonacea (Cnidaria: Octocorallia). Like all octocorals, they possess polyps with an eight-part symmetry, but also produce a central skeletal axis that can be comprised of different mixtures of proteinaceous material (gorgonin) and /or calcitic material [1], and their colonial growth forms consist of various vertically-branching morphologies. Gorgonian octocorals are abundant in tropical reef environments in the Caribbean/tropical western Atlantic [2–4] and locally abundant in the deep sea [5]. They are ecologically valuable as ecosystem engineers in providing shelter and physical complexity to the reef environment [6] as well as contributing significantly to the reef substrate [7].

Efforts to understand gorgonian octocoral diversity, and their phylogenetic relationships, have been confounded by significant morphological plasticity in their systematic characters, ranging from gross colony/branch structure to sclerite (microscopic skeletal components) morphology [8–17]. Furthermore, the extent to which the environment governs morphological plasticity in gorgonian octocorals, and how strong a role genetic influences play, remains unknown. For example, Prada [18, 19] demonstrated that the differences between shallow (~3m) and deep (~20m) populations of the gorgonian octocoral *Eunicea flexuosa* in sites across the Caribbean were attributable to genetic differences between each lineage, i.e., were the result of local selection, not environmental plasticity. In contrast, in shallow-water *E*. *flexuosa* in the Florida keys, Kim [20] found discrete morphological differences within back reef, shallow forereef, and forereef environments, but no evidence of genetic differentiation among the three reef habitats. Gutierrez-Rodriguez [21] conducted morphological and genetic analyses of the gorgonian octocoral *Pseudopterogogia elisabethae* from three sites in the Bahamas, Florida Keys and Colombia, and, although genetic differences were found across geographic locations, there was no clear correlation between genetic variation and branch/sclerite morphology. Interacting environmental factors such as water flow, depth and biomechanical constraints [22] were considered to play a significant role in shaping morphological differences.

**Fig 1:**
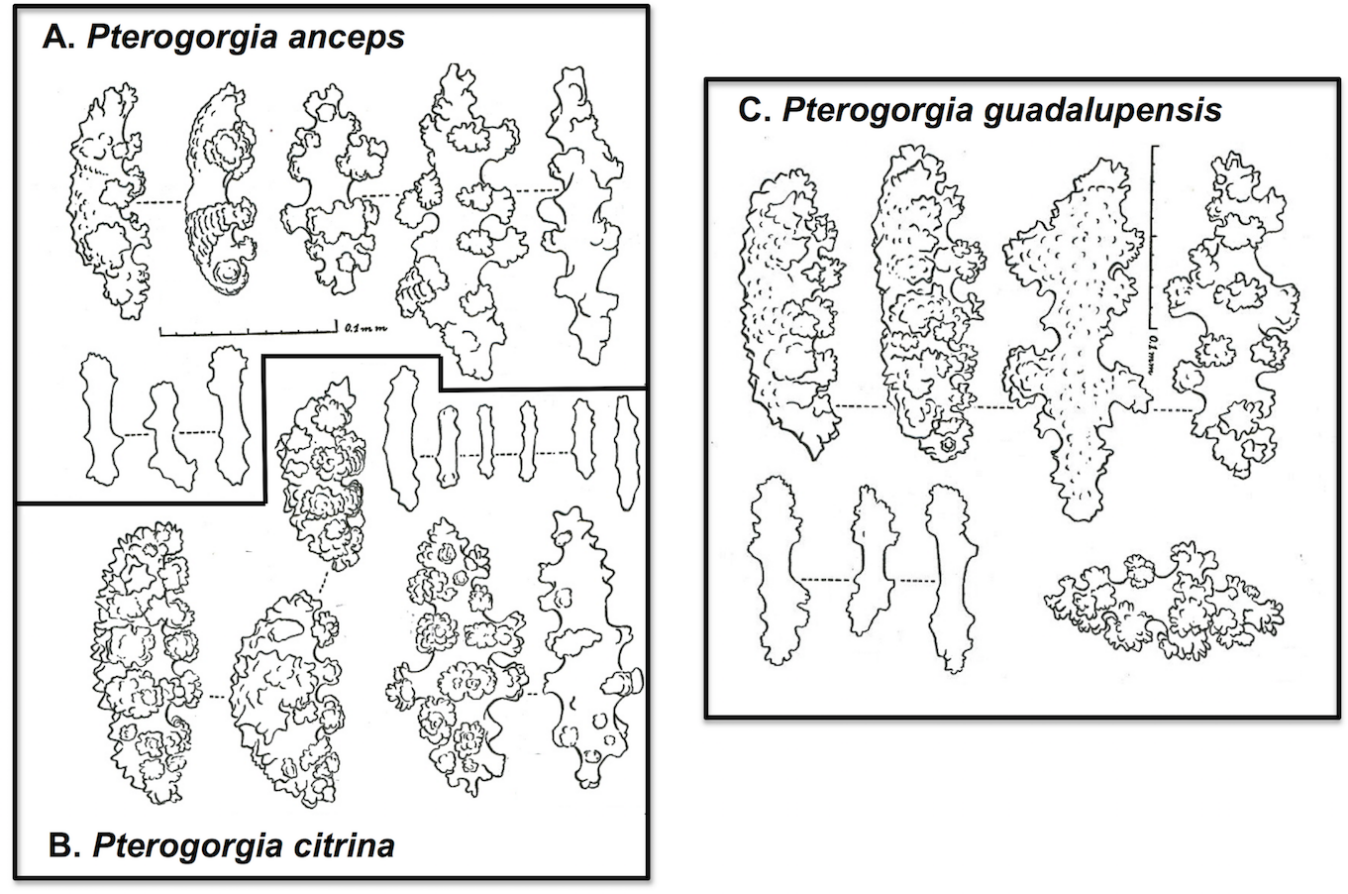
*Pterogorgia* sclerites. Drawings depicting the similarity in sclerite form among the three species of *Pterogorgia*, (A) *P*. *anceps*, (B) *P*. *citrina*, and (C) *P*. *guadalupensis*. As a result, and unlike many gorgoniid octocorals, sclerite morphology is not used as a character to separate and define species of *Pterogorgia*. Drawings adapted from [7].

**Fig 2:**
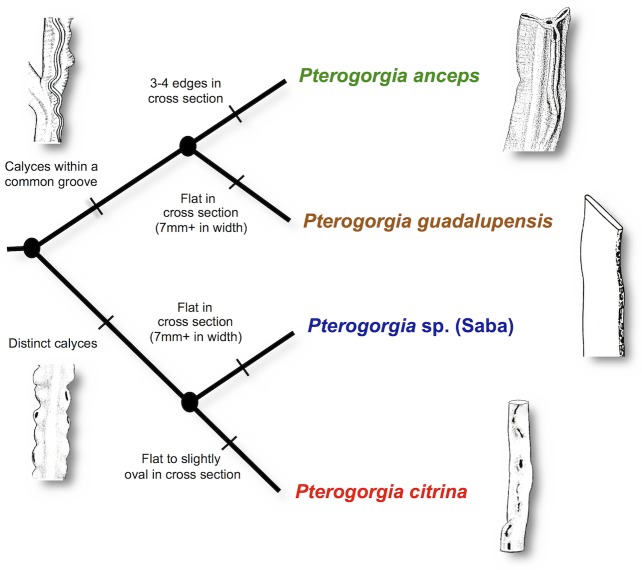
Cladogram depicting hypothesized phylogenetic relationships among *Pterogorgia* species using morphological characters. *P*. *anceps* and *P*. *guadalupensis* share the synapomorphic character “calyces within a common groove”. Autapomorphic characters, branches “3–4 edges in cross section” define *P*. *anceps* while branches “flat in cross section (7mm+ in width)” separate *P*. *guadalupensis*. *P*. *citrina* and the morphotype from Saba Bank share the synapomorphic character “distinct calyces”. *P*. *citrina* contains the autapomorphic character of branches “flat to slightly oval in cross section”, while the Saba Bank morphotype contains branches “flat in cross section (7mm+ in width)”. Drawings adapted from [7, 23, 57].

The gorgonian octocoral genus *Pterogorgia* Ehrenberg, 1834, (Family: Gorgoniidae) consists of three described species, *P*. *anceps* Pallas, 1766; *P*. *citrina* Esper, 1792; and *P*. *guadalupensis* Duchassaing & Michelin, 1846, all endemic to the Caribbean and tropical western Atlantic [7]. Gorgonian octocoral species in the various other genera within the Gorgoniidae are mostly differentiated by morphological differences in sclerite architecture (see [7, 23]). However, sclerites of the three *Pterogorgia* spp. exhibit very similar morphologies, and are not specifically used for defining autapomorphies for each species (Fig 1). Bayer [7] commented “[a]n examination of the spicules [sclerites] of [*P*. *guadalupensis*] reveals that they are little different from those of *P*. *anceps* and *P*. *citrina*” (p. 277). Alternatively, calyx and branch morphology are used to define *Pterogorgia* spp. and can be used to hypothesize evolutionary relationships (Fig 2). Colonies with distinct calyces, each with their own opening, characterize *P*. *citrina*. Colonies with calyces that do not appear as individual openings, but instead form a common groove, are a synapomorphy in *P*. *anceps* and *P*. *guadalupensis*. These two species are distinguished by the shape of their branches in cross section: *P*. *anceps* contains branches that bear 3 to 4 edges when viewed in cross section, while *P*. *guadalupensis* consists of branches that are flat in cross section, and usually over 7mm wide.

**Fig 3:**
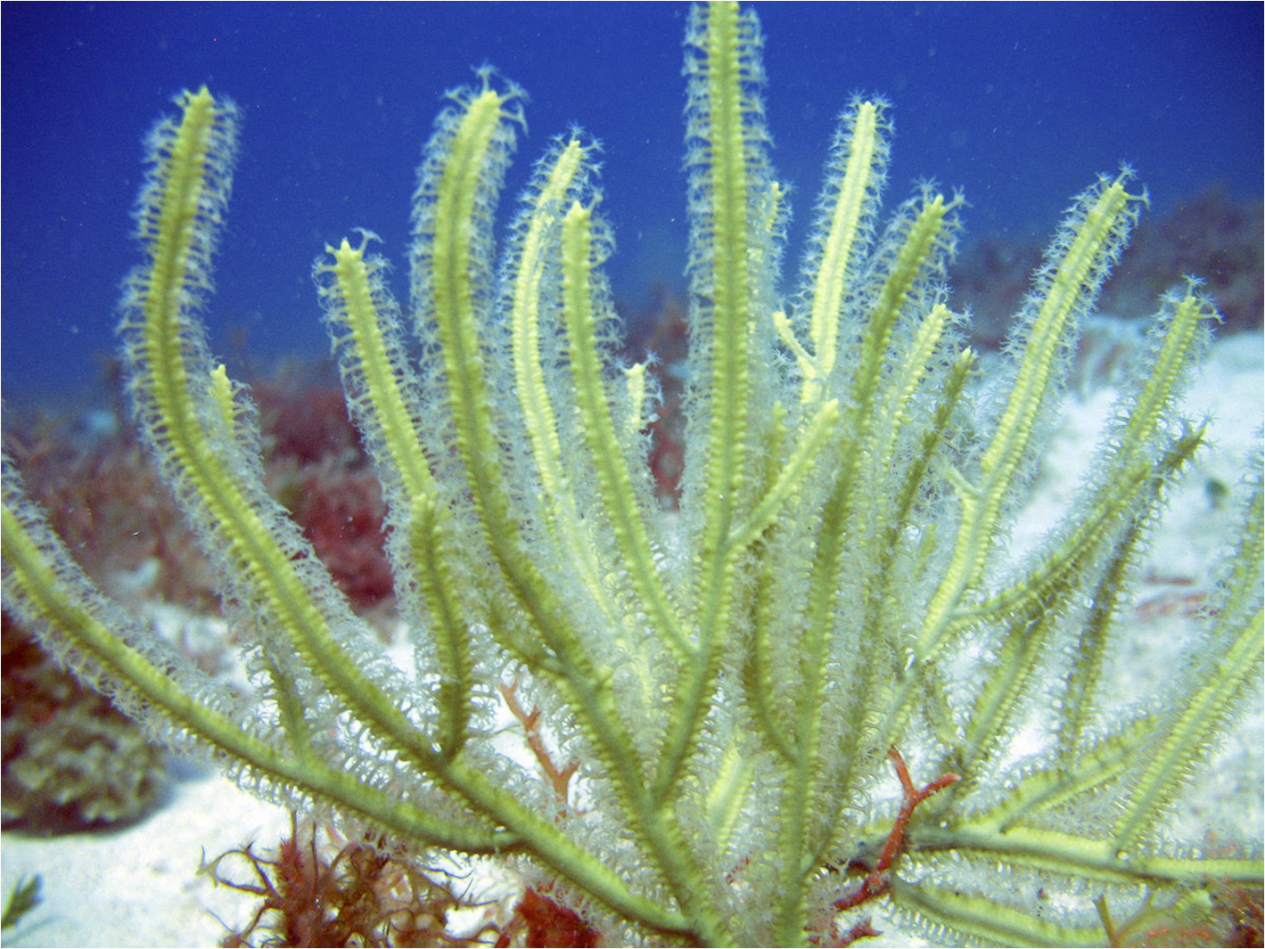
A unique morphotype of *Pterogorgia* from Saba Bank. This morphotype is similar to *P*. *citrina* by containing distinct calyces, but approaches *P*. *guadalupensis* in branch shape with flat branches over 7mm in width. Photo by J.A. Sanchez.

Colonies of a unique morphotype of *Pterogorgia* (Fig 3) were collected from Saba Bank [24], in the northeastern Caribbean (October, 2007). This morphotype exhibited distinct calyces characteristic of *P*. *citrina*, but also consisted of large (>7mm) flat branches not found on other *P*. *citrina* (*sensu stricto*) colonies collected from the same reef environments on Saba Bank. The large flat branches were similar to those of *P*. *guadalupensis* colonies found in the same area. Therefore, a morphological phylogenetic hypothesis places the Saba *Pterogorgia* sp. morphotype as a putative sister species to *P*. *citrina*, but convergent with *P*. *guadalupensis* with respect to branch morphology (Fig 2).

Molecular markers for lower-level phylogenetic studies in octocorals have been useful (e.g., [15, 25]), and attempts to utilize mitochondrial loci (e.g., *mtMutS*+*COI*+*igr*1) for molecular barcoding of octocoral species have yielded practical, albeit limited, results [26] despite the relative lack of mitochondrial sequence variation found in anthozoans [27] and the dearth of nuclear markers available for octocorals [28]. Therefore, despite the lack of a clear “barcoding gap” (i.e., maximum intraspecific and minimum interspecific genetic overlap), some octocoral species may be distinguished using mitochondrial genetic distances or nucleotide character-based criteria.

In addition to the mitochondrial loci available, very few nuclear markers have been utilized for species-level diagnoses in gorgonian octocorals. The most common are the ribosomal transcribed spacer (*ITS*) regions, which have been shown to exhibit suitable variation for inter- and intra-generic phylogenetic analyses [29–31]. However, because nuclear ribosomal loci are multi-copy, sequence variants within individuals may confound the phylogenetic signal of this marker. An *SRP54* intron (referred to simply as *SRP54*) is a presumed single copy nuclear marker that has been used for among- and within-species analyses in both octocorals and scleractinian corals [32–34]. Pairwise sequence divergences suggest that *SRP54* may adequately resolve congeneric relationships within these groups. However, significant length variation in intron size across taxa, and the technical difficulty of PCR-amplifying *SRP54* among disparate taxonomic groups, has hindered the assimilation of this marker into comprehensive phylogenetic analyses of octocorals [28].

An accurate assessment of diversity on Saba Bank is crucial for effective conservation polices [35] and the monitoring of the effects of environmental change or disturbance over time [36]. In order to better quantify the evolutionary diversity within *Pterogorgia*, molecular phylogenetic analyses were used to test morphological species hypotheses among individuals of the three described species of *Pterogorgia* and the unusual morphotype from Saba Bank. Partial gene fragments of mitochondrial *mtMutS*, cytochrome *b*, and *igr4*, along with two hyper-variable nuclear loci, *ITS2* and *SRP54*, were used to determine (1) whether calyx and branch morphology are valid systematic characters in *Pterogorgia*, and (2) whether there is genetic evidence for the unusual *Pterogorgia* sp. morphotype from Saba Bank as a new (phylogenetic) species.

## Materials and Methods

### Sample Collection

*Pterogorgia* spp. samples from Saba Bank (USNM1122721-USNM1122730) were obtained by permission from the Department of Invertebrate Zoology at the Smithsonian National Museum of Natural History, Washington, DC, USA, originally collected as part of a biodiversity assessment of the area (see [24]). Samples collected from southeastern Florida (PCFK1-PCFK6; PAFK1-PAFK5; PAFL6) were deposited into the permanent collection of the Coral Reef Conservation Research Laboratory, University of Miami, USA (Table 1). Collection permits were issued by the National Park Service (Biscayne National Park) and the Florida Fish and Wildlife Conservation Commission. Sub-samples of three individuals of *Pterogorgia citrina*, four individuals of *P*. *guadalupensis*, one individual of *P*. cf. *anceps*, and two individuals of a putative new morphospecies of *Pterogorgia*, were used from Saba Bank. In order to incorporate variation within each species across its geographic range, six individuals of *P*. *citrina* and *P*. *anceps* were used from reefs in south eastern Florida. Samples of *P*. *guadalupensis* were only available for analysis from Saba Bank. In total, nine individuals of *P*. *citrina*, seven of *P*. *anceps*, five of *P*. *guadalupensis*, and two of a putative new morphospecies of *Pterogorgia* were utilized for phylogenetic analysis (Table 1). All samples were collected using SCUBA or by freediving. Branch tips of ~3cm were cut from all colonies and stored in 10mL vials of 95% ethanol.

**Table 1:** Sample information including Voucher/Sample ID*, sample collection locations, and GenBank Accession Numbers. USNM vouchers were relabeled with an "SB" (Saba Bank) for their sample ID in the text and figures.

				GenBank Accession #s			
Genus	Species	Voucher/Sample ID*	Sample Location	*mtMutS*	*COI + igr4*	*ITS2*	*SRP54*
*Pterogoriga*	*citrina*	PCFK1	East Washerwoman Shoal, Florida Keys	KP687619	KP713966	KP687649	KP687704
						KP687650	KP687705
*Pterogoriga*	*citrina*	PCFK2	East Washerwoman Shoal, Florida Keys	KP687620	KP713967	KP687651	KP687706
						KP687652	KP687707
*Pterogoriga*	*citrina*	PCFK3	East Washerwoman Shoal, Florida Keys	KP687621	KP713968	KP687653	KP687708
*Pterogoriga*	*citrina*	PCFK4	East Washerwoman Shoal, Florida Keys	KP687622	KP713969	KP687654	KP687709
						KP687655	
						KP687656	
*Pterogoriga*	*citrina*	PCFK5	East Washerwoman Shoal, Florida Keys	KP687623	KP713970	KP687657	KP687710
						KP687658	
						KP687659	
						KP687660	
*Pterogoriga*	*citrina*	PCFK6	East Washerwoman Shoal, Florida Keys	KP687624	KP713971	KP687661	KP687711
						KP687662	
						KP687663	
*Pterogoriga*	*citrina*	USNM1122722, SB722	Saba Bank	KP687625	KP713972	KP687664	KP687712
						KP687665	
						KP687666	
*Pterogoriga*	*citrina*	USNM1122723, SB723	Saba Bank	KP687626	KP713973	KP687667	KP687713
							KP687714
							KP687715
*Pterogoriga*	*citrina*	USNM1122724, SB724	Saba Bank	KP687627	KP713974	KP687668	KP687716
						KP687669	
*Pterogorgia*	sp.	USNM1122730, SB2	Saba Bank	KP687634	KP713981	KP687686	KP687730
							KP687731
							KP687732
*Pterogorgia*	sp.	SB1	Saba Bank	KP687633	KP713980	KP687683	KP687727
						KP687684	KP687728
						KP687685	KP687729
*Pterogorgia*	*anceps*	PAFK1	Key Largo, Florida Keys	KP687612	KP713960	KP687638	KP687690
						KP687639	KP687691
						KP687640	
*Pterogorgia*	*anceps*	PAFK2	Key Largo, Florida Keys	KP687613	KP713961	KP687641	KP687692
							KP687693
							KP687694
							KP687695
*Pterogorgia*	*anceps*	PAFK3	Key Largo, Florida Keys	KP687614	KP713962	KP687642	KP687696
						KP687643	KP687697
							KP687698
							KP687699
*Pterogorgia*	*anceps*	PAFK4	Key Largo, Florida Keys	KP687615	KP713963	KP687644	KP687700
						KP687645	KP687701
*Pterogorgia*	*anceps*	PAFK5	Key Largo, Florida Keys	KP687616	KP713964	KP687646	KP687702
*Pterogorgia*	*anceps*	PAFL6	Long Reef, Florida (Bicayne National Park)	KP687617	KP713959	KP687647	KP687703
						KP687648	
*Pterogorgia*	cf. *anceps*	USNM1122721, SB721	Saba Bank	KP687618	KP713965	KP687635	KP687687
						KP687636	KP687688
						KP687637	KP687689
*Pterogorgia*	*guadalupensis*	USNM1122725, SB725	Saba Bank	KP687628	KP713975	KP687670	KP687717
						KP687671	KP687718
*Pterogorgia*	*guadalupensis*	USNM1122726, SB726	Saba Bank	KP687629	KP713976	KP687672	KP687719
*Pterogorgia*	*guadalupensis*	USNM1122727, SB727	Saba Bank	KP687630	KP713977	KP687673	KP687720
						KP687674	KP687721
						KP687675	KP687722
*Pterogorgia*	*guadalupensis*	USNM1122728, SB728	Saba Bank	KP687631	KP713978	KP687676	KP687723
						KP687677	KP687724
						KP687678	KP687725
						KP687679	
*Pterogorgia*	*guadalupensis*	USNM1122729, SB729	Saba Bank	KP687632	KP713979	KP687680	KP687726
						KP687681	
						KP687682	

* Repository Information: USNM samples deposited in the Smithsonian National Museum of Natural History, Washinton, DC, USA; PCFK, PAFK, SB1 samples deposited in the Coral Reef Conservation Research Laboratory, University of Miami, USA.

### Molecular Analyses–DNA Extraction, PCR, Cloning and Sequencing

Total genomic DNA was extracted from each sample using a modified organic protocol [37]. Final elution was in 100μL of TE buffer and samples were stored temporarily at -20°C and permanently at -80°C. The Polymerase Chain Reaction (PCR) was used to amplify two nuclear loci (the internal transcribed spacer 2 [*ITS2*] region of ribosomal DNA, and the signal recognition particle 54 [*SRP54*] intron), and partial gene regions of three mitochondrial loci (*mtMutS*; cytochrome *b*; and intergenic region 4 [*igr*4]). Primer (oligonucleotide) sequences for *ITS2*, 5.8S-436: 5’- AGC ATG TCT GTC TGA GTG TTG G -3’ and 28S-663: 5’- GGG TAA TCT TGC CTG ATC TGA G -3’ [31] amplified a 210bp product (~166bp with primers removed) with partial segments of *5*.*8S* and *28S* regions at the 3’ and 5’ ends, respectively. Primer sequences for *SRP54* intron, SRP54F: 5’- ATG GGT GAY ATY GAA GGA CTG ATW GAT AAA GTC AA -3’ and SRP54R: 5’- TTC ATG ATG TTY TGG AAT TGY TCA TAC ATG TC -3’ [38] amplified a 206bp product (~143bp with primers removed) with small segments of the exon on either end of the intron. The three mitochondrial loci were amplified with two primer sets. The 5’ end of *mtMutS* (~620bp used for analyses) was amplified using ND42599F: 5’- GCC ATT ATG GTT AAC TAT TAC -3’ and Mut3458R: 5’- TSG AGC AAA AGC CAC TCC -3’ [39–40]. CytbBam1279-F: 5’- AGG AGC CAA TCC AGT AGA GGA ACC -3’ and ND6Bam1648-R: 5’- TAY AGG TAA GAA ATG CGA GTG ATC -3’ [41] amplified a 130bp product of the 3’ end of cytochrome *b* (~66bp used for analyses), the entire *igr*4 region (40bp), and ~257bp of the 5’ end of *ND6*. Of the ~257bp of *ND6*, only one site was variable among all of the *Pterogorgia* spp. samples. Therefore, this gene region was not used for phylogenetic analyses.

PCR-amplifications used 10pmol of each primer, 200μM dNTPS, 2mM MgCl_2_, and 1μL of genomic DNA (varying final elution concentrations) using 0.6U of Taq polymerase (GoTaq, Promega, Inc.) and manufacturer provided buffers. Sterile DNA-grade water (VWR, Inc.) was added to make a final reaction volume of 25μL. Thermal cycler reaction parameters included an initial denaturing step of 94°C for 3 min, followed by 35 rounds of 94°C for 1 min, an annealing temperature of 55°C for *mtMutS*, cytochrome *b*/*igr*4 and *ITS2*, or 45°C *SRP54* for 1 min, and extension at 74°C for 1 min. A final extension step of 72°C for 7 min completed the reaction. PCR products for the nuclear loci, *ITS2* and *SRP54*, were cloned using pGEM-T Easy Vector Systems (Promega, Inc.) following manufacturer protocols. Successfully transformed and cloned bacterial colonies were used as template DNA for PCR by picking the plated bacterial colony with a pipette tip and inserting it into a PCR-ready reaction. M13 primers (M13F 5’-TGT AAA ACG ACG GCC AGT-3’) and M13R 5’-CAG GAA ACA GCT ATG ACC-3’), which flanked the insertion region of the vector, were used to amplify the cloned inserts. Eight clones for each locus (*ITS2* and *SRP54*) were sequenced for each individual. Duplicate clones found for each individual were discarded in later analyses. PCR products for *mtMutS* and cytochrome *b*/*igr*4 were directly sequenced. Sequencing was performed using an ABI 3130xl automated DNA sequencer. Sequence contigs were collated and edited using Geneious Pro 4.8.5 [42].

### Phylogenetic Analyses

Nuclear and mitochondrial loci were analyzed both separately and jointly (as a concatenated data set). Phylogenetic relationships for each nuclear locus, *ITS2* and *SRP54*, were first examined using separate data sets for each marker. One contained all unique sequence clones found for each individual (therefore, one individual could contain more than one sequence in a tree). The other contained a single consensus sequence for each individual. Consensus sequences were created by reducing variable positions found among clones to ambiguity codes for each individual. This allowed for concatenation with the sequences of the mitochondrial loci, which were not cloned. Because the ITS2 clones contained intra-individual variation that matched interspecific variation, and, thus, contained minimal phylogenetic information, they were excluded from the concatenation of all loci. In total, phylogenetic reconstruction was performed on six data sets (S1 Fig): *ITS2* –(1) unique clones and (2) consensus alignments; *SRP54* - (3) unique clones and (4) consensus alignments; (5) Mitochondrial concatenation of *mtMutS*, cytochrome *b*, *igr*4; and (6) concatenation of all loci excluding *ITS2*. Edited sequences were aligned using MUSCLE (Multiple Sequence Comparison by Log-Expectation) [43], and trimmed to nearest variable position in the alignment on either side. jModelTest 0.1.1 [44–45], using Akaike Information Criterion (AIC) was used to determine the best model of nucleotide sequence evolution for each locus: *ITS2* (unique clones alignment–K80+G, shape = 0.2910; consensus alignment—K80+I, pinv = 0.9850), *SRP54* (unique clones alignment—GTR+I, pinv = 0.7610; consensus alignment–HKY), *mtMutS* (HKY+I+G, shape = 0.0210, pinvar = 0.9200), cytochrome *b* (HKY+I+G, shape = 0.0230, pinvar = 0.9090), *igr*4 (HKY+I, pinvar = 0.9620).

Bayesian Inference (BI) was performed using MrBayes 3.1.2 [46], Maximum Parsimony analyses were completed using POY 4.1.2.1 [47], and Maximum Likelihood (ML) analyses were conducted using RAxML v.8 [48]. All Bayesian analyses consisted of two independent runs with four chains, with trees sampled every 100^th^ generation. The parameters of the evolutionary models used for each locus were set to match the outputs obtained from jModelTest. For among-site rate variation (+G) and proportion of invariable sites (+I), the options “gamma” and “propinv” were used. When the model required a proportion of the sites as invariable, with the remaining sites drawn from a gamma distribution (+G+I), “invgamma” was used. Concatenated alignments were partitioned into character sets for each locus, and the model of sequence evolution chosen by jModelTest applied to each partition (i.e., locus). All parameters (tratio, statefreq, shape, pinvar) were unlinked to allow each partition to have its own set of parameters, and the overall rate was allowed to differ across partitions by setting the rate prior (prset ratepr) to “variable” for all partitions. Markov chain Monte Carlo (MCMC) runs were carried out for 1 to 5 million generations for all datasets (*ITS2* consensus– 1 million; *ITS2* unique clones– 4 million; mitochondrial concatenation, *SRP54* consensus and unique clones, and total concatenation– 5 million). Convergence for each run was determined when the average standard deviation of split frequencies was <0.01 and the potential scale reduction factor (PSRF) was 1.00. Posterior probabilities were calculated by setting the “burn-in” to 25%, which was sufficient to eliminate trees sampled prior to the runs becoming stationary. MP using POY was applied to all datasets using the Simple Search option with static homology, which utilizes a combination of TBR and SRP branch swapping and random sequence addition with 100 repetitions. Node support was assessed by 1,000 bootstrap replicates for ML and MP. Bremer decay values were calculated using TreeRot v.3 [49].

Three outgroups were used for each dataset (except for *ITS2* alignments, which used two), and consisted of species in different genera within the same family as *Pterogorgia* (Gorgoniidae). *Gorgonia ventalina* and *Pseudopterogorgia acerosa* were used in the mitochondrial and *SRP54* datasets, and *Pinnigorgia flava* was used in the *ITS2* dataset. A species from a separate family (Plexauridae), *Eunicea flexuosa*, was used in all datasets.

## Results

### Mitochondrial Loci

Initially, the phylogenetic information from each mitochondrial locus, *mtMutS*, cytochrome *b* and *igr*4, was explored individually. Each locus contained minimal sequence variation among the three species of *Pterogorgia* and the morphotype from Saba Bank. The trimmed alignment for *mtMutS* contained 620bp with only 9 variable positions, 7 of which were parsimony-informative. The partial sequence of cytochrome *b* contained 66bp with 1 variable position that was also parsimony-informative. The *igr*4 alignment was 40bp in length and also contained 1 variable parsimony-informative site. Initial phylogenetic analyses with each individual mitochondrial locus recovered similar but weakly supported trees. Because of the limited variation among the three mitochondrial loci, a concatenated mitochondrial dataset was used.

**Fig 4:**
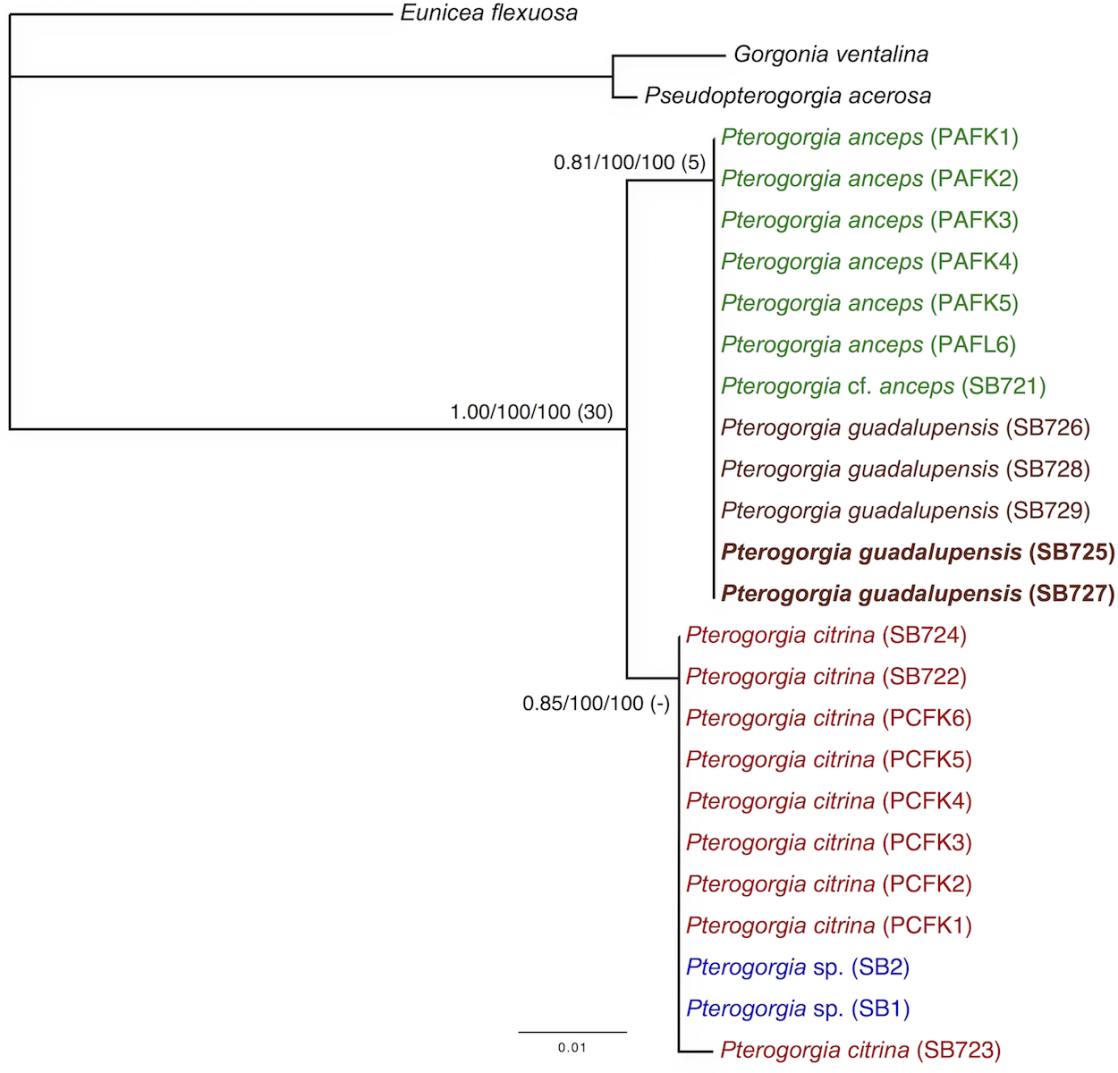
Mitochondrial phylogeny. Phylogram generated by a concatenated mitochondrial data set (*mtMutS* + cytb + *igr*4) using Bayesian Inference (BI), maximum likelihood (ML), and maximum parsimony (MP). Node support for BI, ML and MP is shown from left to right, with Bremer decay values in parentheses. Taxa in bold are individuals whose placement differed between the mitochondrial and *SRP54* (nuclear) phylogenies (see Fig 5).

Phylogenetic reconstruction using the concatenated mitochondrial dataset recovered a tree with two clades that were well-supported with ML and MP analyses, but were deficient with Bremer support and BI (posterior probabilities 81 and 85) (Fig 4). All of the *P*. *anceps* and *P*. *guadalupensis* individuals grouped within a clade, and all of the *P*. *citrina* and *Pterogorgia* sp. individuals grouped in a sister clade. There was no inter- or intra-specific sequence variation among the individuals grouped within each clade, with the exception of *P*. *citrina* (SB723). No geographic variation was found among individuals of the same species collected from eastern Florida and Saba Bank.

### Nuclear Loci

#### *SRP54* intron

**Fig 5:**
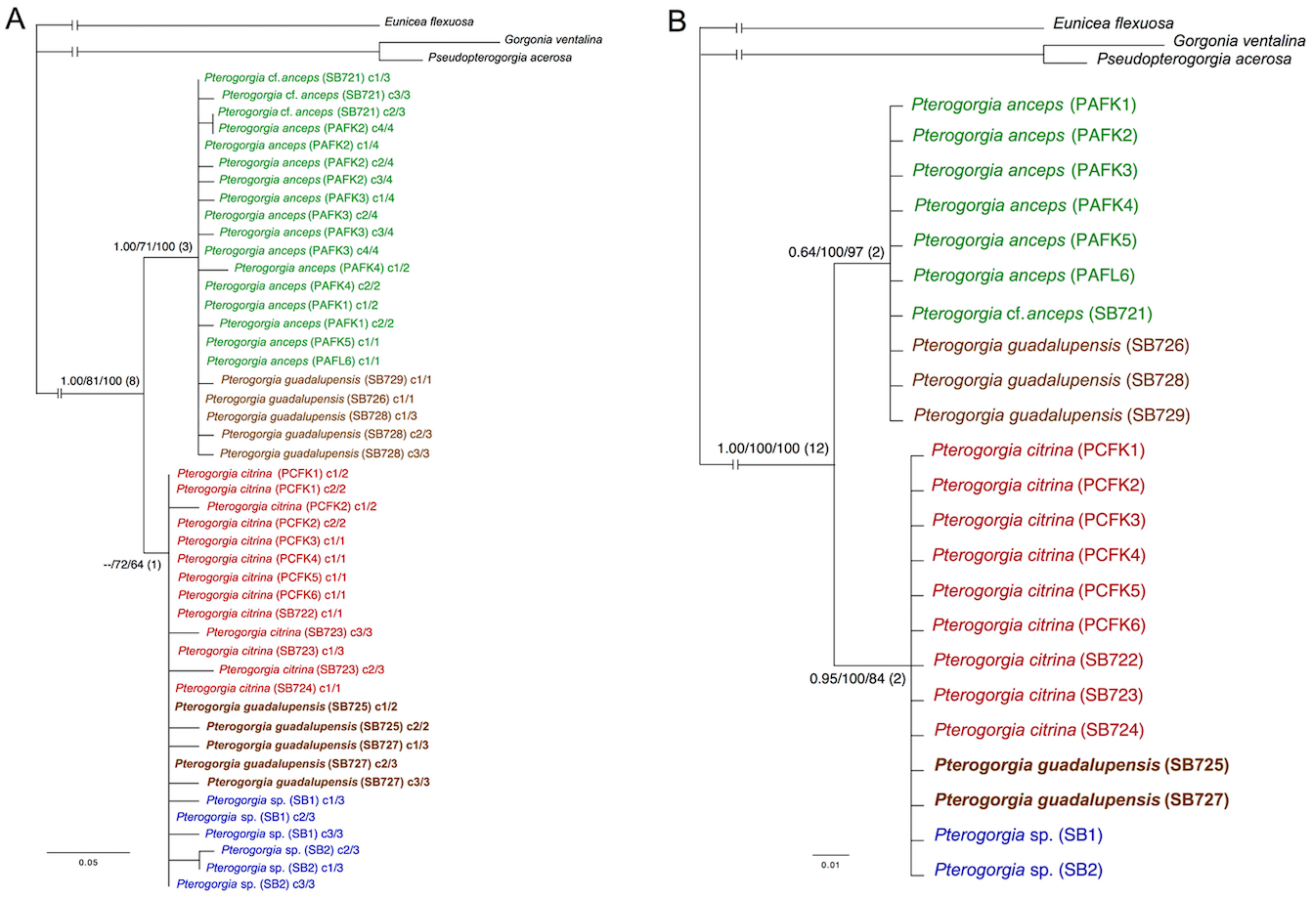
*SRP54* phylogenies. Phylograms generated from *SRP54* sequences for (a) unique clones found for each individual and (b) consensus sequences for each individual using Bayesian Inference (BI), maximum likelihood (ML), and maximum parsimony (MP). Node support for BI, ML and MP is shown from left to right, with Bremer decay values in parentheses. A method with node support <50 contains “—". In (a) clones are coded by a “c” (clone) followed by the number of the clone relative to the total unique clones found for that individual, and arranged by taxonomic affinity for clarity. Taxa in bold are individuals whose placement differed between the *SRP54* (nuclear) and mitochondrial phylogenies (see Fig 4).

Clones from each individual yielded one to four unique *SRP54* sequences, and their alignment contained 7 parsimony-informative and 17 variable but parsimony-uninformative characters. Phylogenetic reconstruction recovered a tree with two primary clades (Fig 5A). None of the three morphospecies of *Pterogorgia*, nor the Saba morphotype, were resolved monophyletically. However, and unlike with the mitochondrial loci (Fig 4), two *P*. *guadalupensis* individuals (SB725 and SB727) grouped within a poorly-supported clade that consisted of the *P*. *citrina* and *Pterogorgia* sp. individuals. The remaining *P*. *anceps* and *P*. *guadalupensis* individuals grouped in a well-supported sister clade. None of the *Pterogorgia* spp. clustered by geographic location. *SRP54* consensus sequences (5 parsimony-informative characters) for each individual similarly recovered a phylogeny with two sister clades (Fig 5B). Similar to the unique clones tree for *SRP54* (Fig 5A), one of the clades contained all of the individuals of *P*. *anceps* and three of the five individuals of *P*. *guadalupensis*. However, support was low with BI. Unlike the unique clones tree, the clade that grouped the *P*. *citrina* and *Pterogorgia* sp. individuals together with two individuals of *P*. *guadalupensis* (SB725 and SB727) was well-supported.

#### *ITS2*

Clones from each individual yielded one to four unique *ITS2* sequences, and their alignment contained 5 parsimony-informative and 22 variable but parsimony-uninformative characters. Phylogenetic reconstruction using these sequences recovered a largely unsupported tree with a single clade that contained clones from some *P*. *anceps* and *P*. *guadalupensis* individuals (S2A Fig). The remaining sequences of *P*. *citrina*, *Pterogorgia* sp., and *P*. *guadalupensis* were unresolved. *ITS2* consensus sequences for each individual (2 parsimony-informative characters in the alignment) recovered a similarly unresolved tree with a single poorly supported clade that grouped clones from most *P*. *anceps* and *P*. *guadalupensis* individuals (S2B Fig).

### Mitochondrial and Nuclear Loci

**Fig 6:**
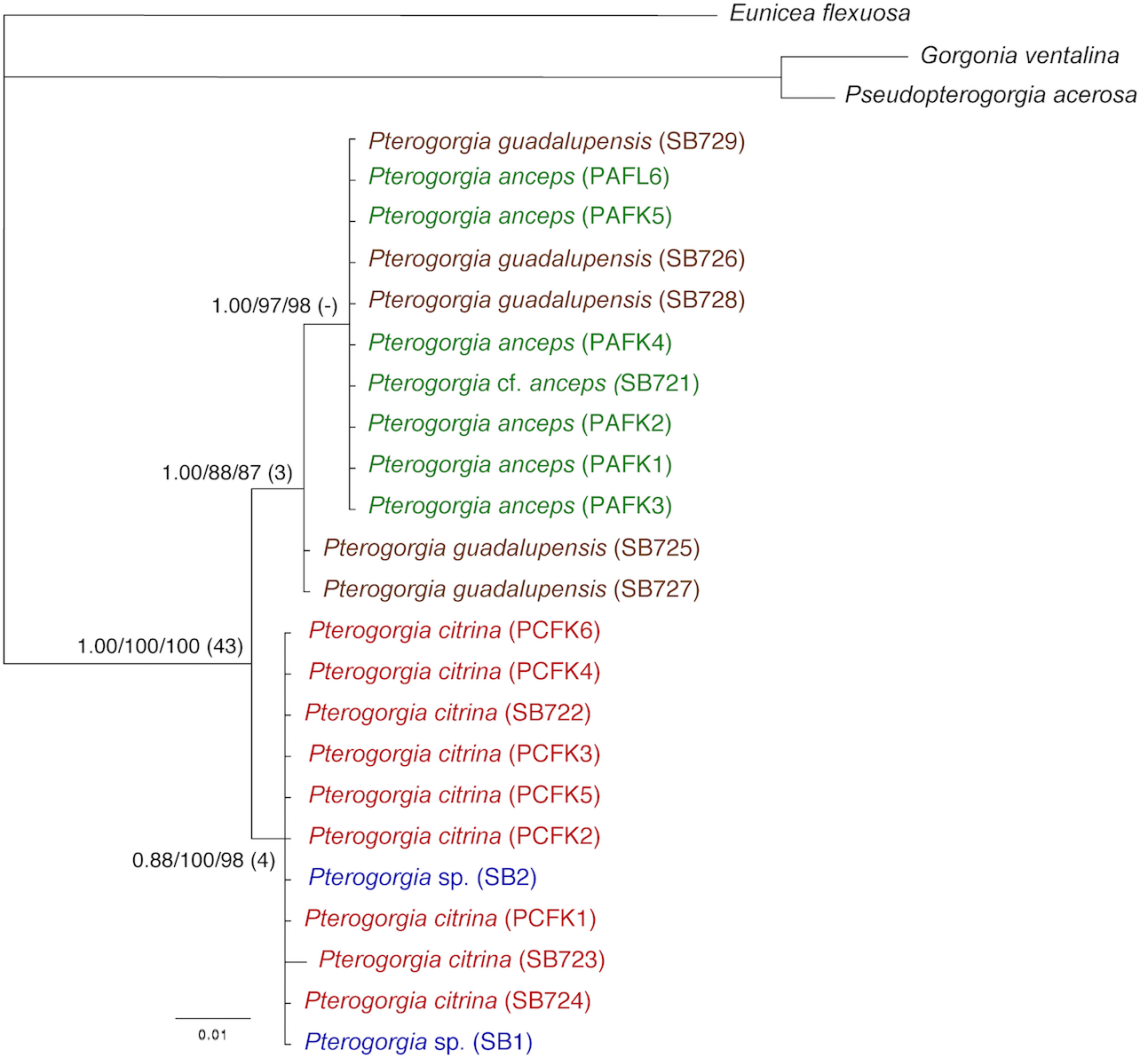
Concatenated dataset. Phylogram generated by a concatenated dataset including three mitochondrial fragments (*mtMutS* + cytb + *igr*4) and one nuclear (*SRP54*) locus using Bayesian Inference (BI), maximum likelihood (ML), and maximum parsimony (MP). Node support for BI, ML and MP is shown from left to right, with Bremer decay values in parentheses.

A concatenated dataset containing fragments of the mitochondrial loci *mtMutS*, cytochrome *b*, and *igr*4, and the nuclear locus *SRP54* (14 parsimony-informative and 2 variable but parsimony-uninformative characters) recovered a tree with two well-supported sister clades (BI was relatively low for the *P*. *citrina*-*Pterogorgia* sp. clade–posterior probability 88) (Fig 6). Similar to the mitochondrial analyses, the *P*. *anceps* and *P*. *guadalupensis* individuals grouped together in one clade. However, unlike the other datasets, *P*. *guadalupensis* was paraphyletic within this clade. A sister clade contained all of the *P*. *citrina* and *Pterogorgia* sp. individuals. There was no support for the separation of *P*. *citrina* and the *Pterogorgia* sp. morphotype.

## Discussion

The mitochondrial and nuclear markers used in this study each demonstrated minimal genetic variation at the interspecific level in *Pterogorgia*. For example, among the mitochondrial fragments used, cytochrome *b* and *igr4* each contained only one parsimony-informative site, and *mtMut*S contained seven. However, the fragments used were relatively short in length, which may have led to a limited number of phylogenetically useful sites. Nevertheless, although interspecific variation was minimal and there was no distance-based “barcoding gap” among the *Pterogorgia* morphospecies, there were variable positions and/or indels (indels with *SRP54* only) among each locus which consistently separated *P*. *citrina* and *Pterogorgia* sp. from *P*. *anceps* and *P*. *guadalupensis*, and which could serve as nucleotide character-based “barcodes” [26]. However, these “barcodes” would not be able to differentiate between the morphospecies *P*. *anceps* and *P*. *guadalupensis* or *P*. *citrina* and the morphotype from Saba.

Although the nuclear loci (*ITS2* and *SRP54*) contained more overall variation, much of it was uninformative and consisted of intra-individual and/or unshared single nucleotide differences that may have been artificially generated by cloning [50–51]. These uninformative sites may have contributed to the lower node support observed in the *P*. *citrina—Pterogorgia* sp.—*P*. *guadalupensis* clade of the *SRP54* unique clones phylogeny (Fig 5A). When these substitutions are accounted for, *SRP54* did not contain any more interspecific variation than the mitochondrial loci. Therefore, although it is considered hyper-variable, *SRP54* may not be useful as a reliable species-level marker among other gorgonian octocoral taxa. With *ITS2*, although it has been useful as a species-level marker in other gorgonian taxa (see Introduction), with *Pterogorgia* it was less informative than the mitochondrial loci at the interspecific level, and was not able to recover a supported phylogeny. Consequently, these data fundamentally consist of two loci, one mitochondrial (the combined *mtMutS*+cytochrome *b*+*igr4*) and one nuclear (*SRP54*).

*SRP54* is considered a single copy gene [32], yet three to four unique sequences were recovered from clones from individuals of each *Pterogorgia* species, indicating that there may be multiple copies of this gene. However, a third or fourth *SRP54* sequence variant was only recovered once among individuals (S1 Table), suggesting that Taq error might explain some of these cloned sequence variants. Sequencing more clones per individual would help determine whether *SRP54* is indeed multi-copy.

The mitochondrial and *SRP54* data generated slightly conflicting phylogenies. The difference was the placement of two of the five *P*. *guadalupensis* individuals (SB725 and SB727). For example, the mitochondrial loci grouped all of the *P*. *guadalupensis* and *P*. *anceps* individuals in a single clade, and all of the *P*. *citrina* and *Pterogorgia* sp. individuals in a sister clade (Fig 4). However, *SRP54* grouped the *P*. *guadalupensis* individuals SB725 and SB727 within a sister clade that contained all of the *P*. *citrina* and *Pterogorgia* sp. individuals (Fig 5B). A concatenated dataset of these markers recovered all of the *P*. *guadalupensis* individuals in a clade together with the *P*. *anceps* individuals but in an unresolved subclade (Fig 6). Nevertheless, this result was likely influenced by the relatively longer and more informative mitochondrial dataset, whose loci have been shown to be useful at the interspecific level [26]. Alternatively, the discrepancy between the nuclear and mitochondrial phylogenies may be explained by competing hypotheses based on contrasting evolutionary histories of each marker/incomplete lineage sorting [52] or hybridization/reticulate evolution [53–54]. For example, in the scleractinian coral genus *Acropora*, molecular analyses suggest that hybridization has resulted in several species complexes (or syngameons) [55].

Overall, these data did not support the *Pterogorgia* sp. from Saba Bank as a separate species, or even *P*. *anceps* and *P*. *guadalupensis* (two longstanding morphological species that are generally uncontested in the literature [7, 23, 56]) as distinct species. However, two well-supported clades were recovered among the *Pterogorgia* spp. with the mitochondrial and concatenated datasets, suggesting that there are at least two lineages within the genus at the species level. One lineage consists of the morphospecies *P*. *anceps* and *P*. *guadalupensis*, and the other contains *Pterogorgia* sp. and *P*. *citrina*. This phylogenetic arrangement is in general agreement with what is hypothesized based on morphology, albeit less resolved (see Fig 2). It confirms the morphological relatedness of *P*. *anceps* and *P*. *guadalupensis*, but cannot differentiate among them. For example, the character “calyces within a common groove” is supported as a synapomorphy of the *P*. *anceps*—*P*. *guadalupensis* clade. However, the separation of the two morphospecies based on branch morphology (e.g., branches “flat” or “3–4 edges” in cross section) was not supported despite conspicuous differences between them. Bayer [23], after examining material collected from the Gulf of Mexico, commented that *P*. *guadalupensis* “is perfectly distinct and worthy of recognition. I have been unable to find specimens of *P*. *anceps* that grade into it”. Since then, however, unusual specimens of *P*. *anceps* have been collected from Panama and the Bahamas with aberrant morphologies (Juan Sanchez, per. comm.). In addition, the specimen of *Pterogorgia* cf. *anceps* (SB721) collected from Saba Bank appeared most like *P*. *anceps*, but had branches whose morphology approached that of *P*. *guadalupensis*.

Similarly, the morphological character “distinct calyces” was supported as a synapomorphy of the *P*. *citrina*—*Pterogorgia* sp. clade. However, branch morphology among *P*. *citrina* and *Pterogorgia* sp. (e.g., “oval” or “flat”) was not supported as a species-level character. Therefore, the large (>7mm) flat branches of the novel *Pterogorgia* sp. may represent an unusual morphological type of *P*. *citrina*, possibly unique to Saba Bank, within a larger genetic lineage of *P*. *citrina* that encompasses various branch morphologies. In this case, the branch morphology of the novel *Ptergorgia* sp. is convergent with those of *P*. *guadalupensis*.

Together, these results suggest that, in common with other octocoral taxa [26, 28], the variation exhibited by these molecular markers may not be sufficient to distinguish morphospecies within each of the phylogenetic clades recovered. Despite this, it is clear that branch morphology may not be reliable for differentiating *Pterogorgia* species. Morphological differences observed in branch morphology among members of the two recovered lineages may instead represent intraspecific colony variation. A reanalysis of sclerite morphology among the morphotypes that fall within each of the two genetic lineages of *Pterogorgia* is required in order to more accurately delineate *Pterogorgia* species, and, ultimately, reevaluate the taxonomy of the genus. This, together with more robust geographic sampling and the incorporation of additional nuclear and/or population-level markers, will help to further illuminate the molecular and morphological divide within *Pterogorgia*.

## Supporting Information

S1 Fig Datasets. Alignments (in Nexus format) used for phylogenetic analyses. *ITS2* –(1) unique clones (2) consensus; *SRP54* - (3) unique clones (4) consensus; (5) Mitochondrial loci—*mtMutS*, cytochrome *b*, *igr*4; and (6) concatenation of all loci excluding *ITS2*.
(PDF)

S2 Fig *ITS2* phylogenies. Phylograms generated from *ITS2* sequences for (a) unique clones found for each individual and (b) consensus sequences for each individual using Bayesian Inference (BI), maximum likelihood (ML), and maximum parsimony (MP). Node support for BI, ML and MP is shown from left to right. A method with node support <50 contains “—". In (a) clones are coded by a “c” (clone) followed by the number of the clone relative to the total unique clones found for that individual.
(PDF)

S1 Table *SRP54* clones. The number of clones found for each individual for *SRP54*.
(PDF)
