# Supplementary material for: Molecular and Morphological Species Boundaries in the Gorgonian Octocoral Genus Pterogorgia (Octocorallia: Gorgoniidae)
Source: PLoS One. 2015 Jul 21;10(7):e0133517. doi: 10.1371/journal.pone.0133517 (PMC4510298; doi:10.1371/journal.pone.0133517)
Supplement: S1 Fig — Alignments (in Nexus format) used for phylogenetic analyses. ITS2 –(1) unique clones (2) consensus; SRP54 - (3) unique clones (4) consensus; (5) Mitochondrial loci—mtMutS, cytochrome b, igr4; and (6) concatenation of all loci excluding ITS2. (PDF) [file pone.0133517.s001.pdf]

Figure S1. Alignments (in Nexus format) used for phylogenetic analyses. *ITS2* – (1) unique clones (2) consensus; *SRP54* - (3) unique clones (4) consensus; (5) Mitochondrial loci - *mtMutS*, cytochrome b, *igr*4; and (6) concatenation of all loci excluding *ITS2*.

(1) *ITS2* unique

```
#NEXUS
begin taxa;
	dimensions ntax=54;
	taxlabels
	'Pterogorgia_anceps_FK1_c2/3'[&description=""]
	'Pterogorgia_guadalupensis_SB727_c1/3'[&description=""]
	'Pterogorgia_anceps_FK3_c1/2'[&description=""]
	'Pterogorgia_anceps_FK1_c1/3'[&description=""]
	'Pterogorgia_guadalupensis_SB725_c1/2'[&description=""]
	'Pterogorgia_guadalupensis_SB727_c2/3'[&description=""]
	'Pterogorgia_anceps_FK4_c2/2'[&description=""]
	'Pterogorgia_anceps_FKlr6_c2/2'[&description=""]
	'Pterogorgia_anceps_cf_SB721_c2/2'[&description=""]
	'Pterogorgia_anceps_cf_SB721_c3/3'[&description=""]
	'Pterogorgia_anceps_FK1_c3/3'[&description=""]
	'Pterogorgia_anceps_FK2_c1/1'[&description=""]
	'Pterogorgia_anceps_FK3_c2/2'[&description=""]
	'Pterogorgia_anceps_FK4_c1/2'[&description=""]
	'Pterogorgia_anceps_FK5_c1/1'[&description=""]
	'Pterogorgia_anceps_FKlr6_c1/2'[&description=""]
	'Pterogorgia_guadalupensis_SB725_c2/2'[&description=""]
	'Pterogorgia_guadalupensis_SB726_c1/1'[&description=""]
	'Pterogorgia_guadalupensis_SB727_c3/3'[&description=""]
	'Pterogorgia_guadalupensis_SB728_c1/4'[&description=""]
	'Pterogorgia_guadalupensis_SB729_c1/3'[&description=""]
	'Pterogorgia_citrina_FKews4_c1/3'[&description=""]
	'Pterogorgia_citrina_FKews1_c1/2'[&description=""]
	'Pterogorgia_guadalupensis_SB728_c4/4'[&description=""]
	'Pterogorgia_guadalupensis_SB729_c2/3'[&description=""]
	'Pterogorgia_citrina_FKews4_c2/3'[&description=""]
	'Pterogorgia_citrina_FKews6_c1/3'[&description=""]
	'Pterogorgia_citrina_FKews6_c2/3'[&description=""]
	'Pterogorgia_citrina_SB722_c1/3'[&description=""]
	'Pterogorgia_citrina_FKews2_c1/2'[&description=""]
	'Pterogorgia_citrina_FKews5_c1/4'[&description=""]
	'Pterogorgia_citrina_FKews5_c2/4'[&description=""]
	'Pterogorgia_citrina_FKews5_c3/4'[&description=""]
	'Pterogorgia_sp_SB1_c3/3'[&description=""]
	'Pterogorgia_guadalupensis_SB728_c2/4'[&description=""]
	'Pterogorgia_citrina_SB724_c1/2'[&description=""]
```

'Pterogorgia_citrina_SB722_c3/3'[&description=""]
'Pterogorgia_sp_SB1_c2/3'[&description=""]
'Pterogorgia_sp_SB2_c1/1'[&description=""]
'Pterogorgia_sp_SB1_c1/3'[&description=""]
'Pterogorgia_anceps_cf_SB721_c1/3'[&description=""]
'Pterogorgia_citrina_FKews1_c2/2'[&description=""]
'Pterogorgia_citrina_FKews2_c2/2'[&description=""]
'Pterogorgia_citrina_FKews3_c1/1'[&description=""]
'Pterogorgia_citrina_FKews4_c3/3'[&description=""]
'Pterogorgia_citrina_FKews5_c4/4'[&description=""]
'Pterogorgia_citrina_FKews6_c3/3'[&description=""]
'Pterogorgia_citrina_SB722_c2/3'[&description=""]
'Pterogorgia_citrina_SB723__c1/1'[&description=""]
'Pterogorgia_citrina_SB724_c2/2'[&description=""]
'Pterogorgia_guadalupensis_SB728_c3/4'[&description=""]
'Pterogorgia_guadalupensis_SB729_c3/3'[&description=""]
Pinnigorgia_flava[&description=""]
Plexaura_flexuosa[&description=""]
;
end;

begin characters;
dimensions nchar=187;
format datatype=dna missing=? gap=-;
matrix
'Pterogorgia_anceps_FK1_c2/3' TTATCAAA-CAATGAACG-
GCACACGTGCTCGTTCGATTGGGG-GTCGCGTAGCG--------------TTGC-
GTTACTATGCGTCTCTCGAATTGCAGTACGTTCTGCTTTCTGCACGAACAAGG
CCTAAAGTGTTTTGCCCTTGCCAGTG---CGTTAGTGGA-
GCCCTCTTACCATTCTTAAC
'Pterogorgia_guadalupensis_SB727_c1/3' TTATCAAA-CAATGAACG-
GCACACGTGCTCGTTCGATTGGGG-GTCGCGTAGCG--------------TTGC-
ATTACTATGCGTCTCTCGAATTGCAGTACGTTCTGCCTTCTGCACGAACAAGG
CCTAAAGTGTTTTGCCCTTGCCAGTG---CGTTAGTGGA-
GCTCTCTTACCATTCTTAAC
'Pterogorgia_anceps_FK3_c1/2' TTATCAAA-CAATGAACG-
GCACACGTGCTCGTTCGGTTGGGG-GTCGCGTAGCG--------------TTGC-
ATTACTATGCGTCTCTCGAATTGCAGTACGTTCTGCTTTCTGCACGAACAAGG
CCTAAAGTGTTTTGCCCTTGCCAGTG---CGTTAGTGGA-
GCTCTCTTACCATTCTTAAC
'Pterogorgia_anceps_FK1_c1/3' TTATCAAA-CAATGAACG-
GCACACGTGCTCGTTCGATTGGGG-GTCGCGCAGCG--------------TTGC-
ATTACTATGCGTCTCTCGAATTGCAGTACGTTCTGCTTTCTGCACGAACAAGG
CCTAAAGTGTTTTGCCCTTGCCAGTG---CGTTAGTGGA-
GCTCTCTTACCATTCTTAAC

'Pterogorgia_guadalupensis_SB725_c1/2' TTATCAAA-CAATGAACG-GCACACGTGCTCGTTCGATTGGGG-GTCGCGTAGCG--------------TTGC-ATTACTATGCGTCTCTCGAATTGCAGTACGTTCTGCTTTCTGCACGAACAAGGCCTAAAGTGTTTCGCCCTTGCCAGTG---CGTTAGTGGA-GCTCTCTTACCATTCTTAAC

'Pterogorgia_guadalupensis_SB727_c2/3' TTATCAAA-CAATGAACG-GCACACGTGCTCGTTCGATTGGGG-GTCGCGTAGCG--------------TTGC-ATTACTATGCGTCTCTCGAATTGCAGTACGTTCTGCTTTCTGCACGAACAAGGCCTAAAGTGTTTTGCCCTTGCCAGTG---CGTTAGTGGA-GCTCTCTTATCATTCTTAAC

'Pterogorgia_anceps_FK4_c2/2' TTATCAAA-CAATGAACG-GCACACGTGCTCGTTCGATTGGGG-GTCGCGTAGCG--------------TTGC-ATCACTATGCGTCTCTCGAATTGCAGTACGTTCTGCTTTCTGCACGAACAAGGCCTAAAGTGTTTTGCCCTTGCCAGTG---CGTTAGTGGA-GCTCTCTTACCATTCTTAAC

'Pterogorgia_anceps_FKlr6_c2/2' TTATCAAA-CAATGAACG-GCACACGTGCTCGTTCGATTGGGG-GTCGCGTAGCG--------------TTGC-ATTACTATGCGTCTCTCGAATTGCAGTACGTTCTGCTTTCTGCACGAACAAGGCCTAAAGTGTTTTGCCCTTGCCAGTG---CGTTAGTGGG-GCTCTCTTACCATTCTTAAC

'Pterogorgia_anceps_cf_SB721_c2/2'TTATCAAA-CAATGAACG-GCACACGTGCTCGTTCGATTGGGG-GTCGCGTAGCG--------------TTGC-ATTACTATGCGCCTCTCGAATTGCAGTACGTTCTGCTTTCTGCACGAACAAGGCCTAAAGTGTTTTGCCCTTGCCAGTG---CGTTAGTGGA-GCTCTCTTACCATTCTTAAC

'Pterogorgia_anceps_cf_SB721_c3/3'TTATCAAA-CAATGAACG-GCACACGTGCTCGTTCGATTGGGG-GTCGCGTAGCG--------------TTGC-ATTACTATGCGTCTCTCGAATTGCAGTACGTTCTGCTTTCTGCACGAACAAGGCCTAAAGTGTTTTGCCCTTGCCAGTG---CGTTAGTGGA-GCTCTCTTACCATTCTTAAC

'Pterogorgia_anceps_FK1_c3/3' TTATCAAA-CAATGAACG-GCACACGTGCTCGTTCGATTGGGG-GTCGCGTAGCG--------------TTGC-ATTACTATGCGTCTCTCGAATTGCAGTACGTTCTGCTTTCTGCACGAACAAGGCCTAAAGTGTTTTGCCCTTGCCAGTG---CGTTAGTGGA-GCTCTCTTACCATTCTTAAC

'Pterogorgia_anceps_FK2_c1/1' TTATCAAA-CAATGAACG-GCACACGTGCTCGTTCGATTGGGG-GTCGCGTAGCG--------------TTGC-ATTACTATGCGTCTCTCGAATTGCAGTACGTTCTGCTTTCTGCACGAACAAGGCCTAAAGTGTTTTGCCCTTGCCAGTG---CGTTAGTGGA-GCTCTCTTACCATTCTTAAC

'Pterogorgia_anceps_FK3_c2/2' TTATCAAA-CAATGAACG-GCACACGTGCTCGTTCGATTGGGG-GTCGCGTAGCG--------------TTGC-ATTACTATGCGTCTCTCGAATTGCAGTACGTTCTGCTTTCTGCACGAACAAGGCCTAAAGTGTTTTGCCCTTGCCAGTG---CGTTAGTGGA-GCTCTCTTACCATTCTTAAC

'Pterogorgia_anceps_FK4_c1/2' TTATCAAA-CAATGAACG-GCACACGTGCTCGTTCGATTGGGG-GTCGCGTAGCG--------------TTGC-ATTACTATGCGTCTCTCGAATTGCAGTACGTTCTGCTTTCTGCACGAACAAGGCCTAAAGTGTTTTGCCCTTGCCAGTG---CGTTAGTGGA-GCTCTCTTACCATTCTTAAC

'Pterogorgia_anceps_FK5_c1/1' TTATCAAA-CAATGAACG-GCACACGTGCTCGTTCGATTGGGG-GTCGCGTAGCG--------------TTGC-ATTACTATGCGTCTCTCGAATTGCAGTACGTTCTGCTTTCTGCACGAACAAGGCCTAAAGTGTTTTGCCCTTGCCAGTG---CGTTAGTGGA-GCTCTCTTACCATTCTTAAC

'Pterogorgia_anceps_FKlr6_c1/2' TTATCAAA-CAATGAACG-GCACACGTGCTCGTTCGATTGGGG-GTCGCGTAGCG--------------TTGC-ATTACTATGCGTCTCTCGAATTGCAGTACGTTCTGCTTTCTGCACGAACAAGGCCTAAAGTGTTTTGCCCTTGCCAGTG---CGTTAGTGGA-GCTCTCTTACCATTCTTAAC

'Pterogorgia_guadalupensis_SB725_c2/2' TTATCAAA-CAATGAACG-GCACACGTGCTCGTTCGATTGGGG-GTCGCGTAGCG--------------TTGC-ATTACTATGCGTCTCTCGAATTGCAGTACGTTCTGCTTTCTGCACGAACAAGGCCTAAAGTGTTTTGCCCTTGCCAGTG---CGTTAGTGGA-GCTCTCTTACCATTCTTAAC

'Pterogorgia_guadalupensis_SB726_c1/1' TTATCAAA-CAATGAACG-GCACACGTGCTCGTTCGATTGGGG-GTCGCGTAGCG--------------TTGC-ATTACTATGCGTCTCTCGAATTGCAGTACGTTCTGCTTTCTGCACGAACAAGGCCTAAAGTGTTTTGCCCTTGCCAGTG---CGTTAGTGGA-GCTCTCTTACCATTCTTAAC

'Pterogorgia_guadalupensis_SB727_c3/3' TTATCAAA-CAATGAACG-GCACACGTGCTCGTTCGATTGGGG-GTCGCGTAGCG--------------TTGC-ATTACTATGCGTCTCTCGAATTGCAGTACGTTCTGCTTTCTGCACGAACAAGGCCTAAAGTGTTTTGCCCTTGCCAGTG---CGTTAGTGGA-GCTCTCTTACCATTCTTAAC

'Pterogorgia_guadalupensis_SB728_c1/4' TTATCAAA-CAATGAACG-GCACACGTGCTCGTTCGATTGGGG-GTCGCGTAGCG--------------TTGC-ATTACTATGCGTCTCTCGAATTGCAGTACGTTCTGCTTTCTGCACGAACAAGGCCTAAAGTGTTTTGCCCTTGCCAGTG---CGTTAGTGGA-GCTCTCTTACCATTCTTAAC

'Pterogorgia_guadalupensis_SB729_c1/3' TTATCAAA-CAATGAACG-GCACACGTGCTCGTTCGATTGGGG-GTCGCGTAGCG--------------TTGC-ATTACTATGCGTCTCTCGAATTGCAGTACGTTCTGCTTTCTGCACGAACAAGGCCTAAAGTGTTTTGCCCTTGCCAGTG---CGTTAGTGGA-GCTCTCTTACCATTCTTAAC

'Pterogorgia_citrina_FKews4_c1/3' TTATCAAA-CAATGAACG-GCACACGTGCTCGTTCGATTGGGG-GTCGCGTAGCG--------------TTGC-ATTACTACGCGTCTCTCGAATTGCAGTACGTTCCGCTTTCTGCACGAACAAGGTCTAAAGTGTTTTGCCCTTGTCAGTG---CGTTAGTGGA-GCTCTCTTACCATTCTTAAC

'Pterogorgia_citrina_FKews1_c1/2' TCATCAAA-CAATGAACG-GCACACGTGCTCGTTCGATTGGGG-GTCGCGTAGCG--------------TTGC-ATTACTGCGCGTCTCTCGAATTGCAGTACGTTCCGCTTTCTGCACGAACAAGGCCTAAAGTGTTTTGCCCTTGCCAGTG---CGTTAGTGGA-GCTCTCTTACCATTCTTAAC

'Pterogorgia_guadalupensis_SB728_c4/4' TTATCAAA-CAATGAACG-GCACACGTGCTCGTTCGATTGGGG-GTCGCGTAGCG--------------TTGC-ATTACTACGCGTCTCTCGAATTGCAGTACGTTCCGCTTCCGGCACGAACAAGGCCTAAAGTGTTTTGCCCTTGCCAGTG---CGTTAGTGGA-GCTCTCTTACCATTCTTAAC

'Pterogorgia_guadalupensis_SB729_c2/3' TTATCAAA-CAATGAACG-GCACACGTGCTCGTTCGATTGGGG-GTCGCGTAGCG--------------TTGC-ATTACTACGCGTCTCTCGAATTGCAGTACGTTCCGCTTCCGGCACGAACAAGGCCTAAAGTGTTTTGCCCTTGCCAGTG---CGTTAGTGGA-GCTCTCTTACCATTCTTAAC

'Pterogorgia_citrina_FKews4_c2/3' TTATCAAA-CAATGAACG-GCACACGTGCTCGTTCGATTGGGG-GTCGCGTAGCG--------------TTGC-ATTACTACGCGTCTCTCGAATTGCAGTACGTTCCGCTTTCTGCACGAACAAGGCCCAAAGTGTTTTGCCCTTGCCAGTG---CGTTAGTGGA-GCTCTCTTACCATTCTTAAC

'Pterogorgia_citrina_FKews6_c1/3' TTATCAAA-CAATGAACG-GCACACGTGCTCGTTCGATTGGGG-GTCGCGTAGCG--------------TTGC-ATTACTACGCGTCTCTCGAATTGCAGTACGTTCCGCTTTCTGCACGAACAAGGCCTAAAGTGTTTTGCCCTTGCCAGTG---CGTTAGTGAA-GCTCTCTTACCATTCTTAAC

'Pterogorgia_citrina_FKews6_c2/3' TTATCAAG-CAATGAACG-GCACACGTGCTCGTTCGATTGGGG-GTCGCGTAGCG--------------TTGC-ATTACTACGCGTCTCTCGAATTGCAGTACGTTCCGCTTTCTGCACGAACAAGGCCTAAAGTGTTTTGCCCTTGCCAGTG---CGTTAGTGGA-GCTCTCTTACCATTCTTAAC

'Pterogorgia_citrina_SB722_c1/3' TTATCAAA-CAATGAACG-GCACACGTGCTCGTTCGATTGGGG-GTCGCGTAGCG--------------TTGC-ATTACTACGCGTCTCTCGAATTGCAGTACGTTCCGCTTTCTGCACGAACAAGGCCTAAAGTGTTTTGCCCTCGCCAGTG---CGTTAGTGGA-GCTCTCTTACCATTCTTAAC

'Pterogorgia_citrina_FKews2_c1/2' TTATCAAA-CAATGAACG-GCACACGTGCTCGTTCGATTAGGG-GTCGCGTAGCG--------------TTGC-ATTACTACGCGTCTCTCGAATTGCAGTACGTTCCGCTTTCTGCACGAACAAGGCCTAAAGTGTTTTGCCCTTGCCAGTG---CGTTAGTGGA-GCTCTCTTACCATTCTTAAC

'Pterogorgia_citrina_FKews5_c1/4' TTATCAAA-CAATGAACG-GCACACGTGCTCGTTCGATTAGGG-GTCGCGTAGCG--------------TTGC-ATTACTACGCGTCTCTCGAATTGCAGTACGTTCCGCTTTCTGCACGAACAAGGCCTAAAGTGTTTTGCCCTTGCCAGTG---CGTTAGTGGA-GCTCTCTTACCATTCTTAAC

'Pterogorgia_citrina_FKews5_c2/4' TTATCAAA-CAATGAACG-
GCACACGTGCTCGTTCGATTGGGG-GTCGCGTAGCG--------------TTGC-
ATTACTACGCGTCTCTCGAATTGCAGTACGTTCCGCTTTCCGCACGAACAAGG
CCTAAAGTGTTTTGCCCTTGCCAGTG---CGTTAGTGGA-
GCTCTCTTACCATTCTTAAC
'Pterogorgia_citrina_FKews5_c3/4' TTATCAAA-CAATGAACG-
GCACACGTGCTCGTTCGATTGGGG-GTCGCGTAGCG--------------TTGC-
ATTACTACGCGTCTCTCGAATTGCAGTACGTTCCGCTTTCTGCACGAACAAGG
CCTAAAGTGTTTTGCCCTTGCCAGTG---TGTTAGTGGA-
GCTCTCTTACCATTCTTAAC
'Pterogorgia_sp_SB1_c3/3' TTATCAAACCAATGAACG-
GCACACGTGCTCGTTCGATTGGGG-GTCGCGTAGCG--------------TTGC-
ATTACTACGCGTCTCTCGAATTGCAGTGCGTTCCGCTTTCTGCACGAACAAGG
CCTAAAGTGTTTTGCCCTTGCCAGTG---CGTTAGTGGA-
GCTCTCTTACCATTCTTAAC
'Pterogorgia_guadalupensis_SB728_c2/4' TTATCAAA-CAATGAACG-
GCACACGTGCTCGTTCGATTGGGG-GTCGCGTAGCG--------------TTGC-
ATTACTACGCGTCTCTCGAATTGCAGTACGTTCCGCTTTCTGCACGAACAAGG
CCTAAAGTGTTTTGCCGTTGCCAGTG---CGTTAGTGGA-
GCTCTCTTACCATTCTTAAC
'Pterogorgia_citrina_SB724_c1/2' TTATCAAA-CAATGAACG-
GCACACGTGCTCGTTCGATTGGGG-GTCGCGTAGCG--------------TTGC-
ATTACTACGCGTCTCTCGAATTGCAGTACGTTCCGCTTTCTGCACGAACAAGG
CCTAAAGTGCTTTGCCCTTGCCAGTG---CGTTAGTGGA-
GCTCTCTTACCATTCTTAAC
'Pterogorgia_citrina_SB722_c3/3' TTATCAAACCAATGAACG-
GCACACGTGCTCGTTCGATTGGGG-GTCGCGTAGCG--------------TTGC-
ATTACTACGCGTCTCTCGAATTGCAGTACGTTCCGCTTTCTGCACGAACAAGG
CCTAAAGTGTTTTGCCCTTGCCAGTG---CGTTAGTGGA-
GCTCTCTTACCATTCTTAAC
'Pterogorgia_sp_SB1_c2/3' TTATCAAACCAATGAACG-
GCACACGTGCTCGTTCGATTGGGG-GTCGCGTAGCG--------------TTGC-
ATTACTACGCGTCTCTCGAATTGCAGTACGTTCCGCTTTCTGCACGAACAAGG
CCTAAAGTGTTTTGCCCTTGCCAGTG---CGTTAGTGGA-
GCTCTCTTACCATTCTTAAC
'Pterogorgia_sp_SB2_c1/1' TTATCAAA-CAATGAACG-
GCACACGTGCTCGTTCGATTGGGG-GTCGCGTAGCG--------------TTGC-
ATTACTACGCGTCTCTCGAATTGCAGTACGTTCCGCTTTCTGCACGAACAAGG
CCTAAAGTGTTTTGCCCTTGCCAGTG---CGTTAGTGGA-
GCTCTCTTACCATTCTTAAC
'Pterogorgia_sp_SB1_c1/3' TTATCAAA-CAATGAACG-
GCACACGTGCTCGTTCGATTGGGG-GTCGCGTAGCG--------------TTGC-
ATTACTACGCGTCTCTCGAATTGCAGTACGTTCCGCTTTCTGCACGAACAAGG
CCTAAAGTGTTTTGCCCTTGCCAGTG---CGTTAGTGGA-
GCTCTCTTACCATTCTTAAC

'Pterogorgia_anceps_cf_SB721_c1/3'TTATCAAA-CAATGAACG-GCACACGTGCTCGTTCGATTGGGG-GTCGCGTAGCG--------------TTGC-ATTACTACGCGTCTCTCGAATTGCAGTACGTTCCGCTTTCTGCACGAACAAGGCCTAAAGTGTTTTGCCCTTGCCAGTG---CGTTAGTGGA-GCTCTCTTACCATTCTTAAC

'Pterogorgia_citrina_FKews1_c2/2' TTATCAAA-CAATGAACG-GCACACGTGCTCGTTCGATTGGGG-GTCGCGTAGCG--------------TTGC-ATTACTACGCGTCTCTCGAATTGCAGTACGTTCCGCTTTCTGCACGAACAAGGCCTAAAGTGTTTTGCCCTTGCCAGTG---CGTTAGTGGA-GCTCTCTTACCATTCTTAAC

'Pterogorgia_citrina_FKews2_c2/2' TTATCAAA-CAATGAACG-GCACACGTGCTCGTTCGATTGGGG-GTCGCGTAGCG--------------TTGC-ATTACTACGCGTCTCTCGAATTGCAGTACGTTCCGCTTTCTGCACGAACAAGGCCTAAAGTGTTTTGCCCTTGCCAGTG---CGTTAGTGGA-GCTCTCTTACCATTCTTAAC

'Pterogorgia_citrina_FKews3_c1/1' TTATCAAA-CAATGAACG-GCACACGTGCTCGTTCGATTGGGG-GTCGCGTAGCG--------------TTGC-ATTACTACGCGTCTCTCGAATTGCAGTACGTTCCGCTTTCTGCACGAACAAGGCCTAAAGTGTTTTGCCCTTGCCAGTG---CGTTAGTGGA-GCTCTCTTACCATTCTTAAC

'Pterogorgia_citrina_FKews4_c3/3' TTATCAAA-CAATGAACG-GCACACGTGCTCGTTCGATTGGGG-GTCGCGTAGCG--------------TTGC-ATTACTACGCGTCTCTCGAATTGCAGTACGTTCCGCTTTCTGCACGAACAAGGCCTAAAGTGTTTTGCCCTTGCCAGTG---CGTTAGTGGA-GCTCTCTTACCATTCTTAAC

'Pterogorgia_citrina_FKews5_c4/4' TTATCAAA-CAATGAACG-GCACACGTGCTCGTTCGATTGGGG-GTCGCGTAGCG--------------TTGC-ATTACTACGCGTCTCTCGAATTGCAGTACGTTCCGCTTTCTGCACGAACAAGGCCTAAAGTGTTTTGCCCTTGCCAGTG---CGTTAGTGGA-GCTCTCTTACCATTCTTAAC

'Pterogorgia_citrina_FKews6_c3/3' TTATCAAA-CAATGAACG-GCACACGTGCTCGTTCGATTGGGG-GTCGCGTAGCG--------------TTGC-ATTACTACGCGTCTCTCGAATTGCAGTACGTTCCGCTTTCTGCACGAACAAGGCCTAAAGTGTTTTGCCCTTGCCAGTG---CGTTAGTGGA-GCTCTCTTACCATTCTTAAC

'Pterogorgia_citrina_SB722_c2/3' TTATCAAA-CAATGAACG-GCACACGTGCTCGTTCGATTGGGG-GTCGCGTAGCG--------------TTGC-ATTACTACGCGTCTCTCGAATTGCAGTACGTTCCGCTTTCTGCACGAACAAGGCCTAAAGTGTTTTGCCCTTGCCAGTG---CGTTAGTGGA-GCTCTCTTACCATTCTTAAC

'Pterogorgia_citrina_SB723__c1/1' TTATCAAA-CAATGAACG-GCACACGTGCTCGTTCGATTGGGG-GTCGCGTAGCG--------------TTGC-ATTACTACGCGTCTCTCGAATTGCAGTACGTTCCGCTTTCTGCACGAACAAGGCCTAAAGTGTTTTGCCCTTGCCAGTG---CGTTAGTGGA-GCTCTCTTACCATTCTTAAC

'Pterogorgia_citrina_SB724_c2/2'   TTATCAAA-CAATGAACG-
GCACACGTGCTCGTTCGATTGGGG-GTCGCGTAGCG--------------TTGC-
ATTACTACGCGTCTCTCGAATTGCAGTACGTTCCGCTTTCTGCACGAACAAGG
CCTAAAGTGTTTTGCCCTTGCCAGTG---CGTTAGTGGA-
GCTCTCTTACCATTCTTAAC
'Pterogorgia_guadalupensis_SB728_c3/4'   TTATCAAA-CAATGAACG-
GCACACGTGCTCGTTCGATTGGGG-GTCGCGTAGCG--------------TTGC-
ATTACTACGCGTCTCTCGAATTGCAGTACGTTCCGCTTTCTGCACGAACAAGG
CCTAAAGTGTTTTGCCCTTGCCAGTG---CGTTAGTGGA-
GCTCTCTTACCATTCTTAAC
'Pterogorgia_guadalupensis_SB729_c3/3'   TTATCAAA-CAATGAACG-
GCACACGTGCTCGTTCGATTGGGG-GTCGCGTAGCG--------------TTGC-
ATTACTACGCGTCTCTCGAATTGCAGTACGTTCCGCTTTCTGCACGAACAAGG
CCTAAAGTGTTTTGCCCTTGCCAGTG---CGTTAGTGGA-
GCTCTCTTACCATTCTTAAC
Pinnigorgia_flava
CAAAAAGATGAACGAACGCGCATCGTCGTTCGTTCGAATGGGG-
GTCGCGTAGCGCGCAACTTACTTACTTGCTACTACTACGCGTCCCTCGAAGTG
CAGCACGTTCAGC--
TCTACATGAACAAGGCCTAAAGTTTTTCGTCCTTGCTAGTGAAGCGCTTGAGA
ACGTTCTTTTTACTTTCTTAAC
Plexaura_flexuosa
CAAACCGACARATGCGCGGACGTGYGCGTCCGYGCGACTGAGGTGTC
GCGCCGCCGTRGCGGARCGTCCCTCGAAGTGCTAY-CGTRAG-
CACGCCGCCGTTCATCTGTGCCGCCGTGCGAACAAGGCCTAAAGTGTTTCGA
CCTTGCCAGCACGGCGCGCCGRCGGCGCSCGCGGAGCTTCGAAAC

;
end;

(2) *ITS2* consensus

#NEXUS
begin taxa;
dimensions ntax=25;
taxlabels
Plexaura_flexuosa[&description=""]
Pinnigorgia_flava[&description=""]
Pterogorgia_guadalupensis_SB728_clonecon[&description=""]
Pterogorgia_guadalupensis_SB729_clonecon[&description=""]
Pterogorgia_citrina_FK4_clonecon[&description=""]
Pterogorgia_anceps_FK1_clonecon[&description=""]
Pterogorgia_citrina_FK1_clonecon[&description=""]
Pterogorgia_citrina_FK5_clonecon[&description=""]

Pterogorgia_citrina_FK6_clonecon[&description=""]
Pterogorgia_citrina_FK2_clonecon[&description=""]
Pterogorgia_citrina_SB724_clonecon[&description=""]
Pterogorgia_citrina_SB722_clonecon[&description=""]
Pterogorgia_citrina_FK3_clonecon[&description=""]
Pterogorgia_sp_SB2_clonecon[&description=""]
Pterogorgia_sp_SB1_clonecon[&description=""]
Pterogorgia_anceps_cf_SB721_clonecon[&description=""]
Pterogorgia_citrina_SB723_clonecon[&description=""]
Pterogorgia_guadalupensis_SB727_clonecon[&description=""]
Pterogorgia_anceps_FK3_clonecon[&description=""]
Pterogorgia_anceps_FK4_clonecon[&description=""]
Pterogorgia_anceps_FLlr6_clonecon[&description=""]
Pterogorgia_anceps_FK2_clonecon[&description=""]
Pterogorgia_anceps_FK5_clonecon[&description=""]
Pterogorgia_guadalupensis_SB726_clonecon[&description=""]
Pterogorgia_guadalupensis_SB725_clonecon[&description=""]
;
end;

begin characters;
dimensions nchar=231;
format datatype=dna missing=? gap=-;
matrix
Plexaura_flexuosa TGGT--
CAAACCGACARATGCGCGGACGTGYGCGTCCGYGCGACTGAGGTGTCGCGCC
GC-------------------
CGTRGCGGARCGTCCCTCGAAGTGCTAYCGTRAGCACGCCGCCGTTCATCTGT
GCCGCCGTGCGAACAAGGCCTAAAGTGTTTCGACCTTGCCAGCACGGCGCGC
CGRCGGCGCSCGCGGAGCTTCGAAACCGTTTCCTCGTCCATCTTAAC
Pinnigorgia_flava
TGGTTACAAAAAGATGAACGAACGCGCATCGTCGTTCGTTCGAATGGG
G-
GTCGCGTAGCGCGCAACTTACTTACTTGCTACTACTACGCGTCCCTCGAAGTG
C-------AGCA------CGTTCAGCT------
CTACATGAACAAGGCCTAAAGTTTTTCGTCCTTGCTAGTGAAGCGC--------------
-------TTGAGAACGTTCTTTTACTTTCTTAAC
Pterogorgia_guadalupensis_SB728_clonecon TTAT--CAAA-
CAATGAACG-GCACACGT----GCTCGTTCGATTGGGG-GTCGCGTAGCG-----------
----TTGCATTACTAYGCGTCTCTCGAATTGC-------AGTA------CGTTCYGCTTY----
CKGCACGAACAAGGCCTAAAGTGTTTTGCCSTTGCCAGTGCG---------------------
---TTAGTGGAGCTCTCTTACCATTCTTAAC
Pterogorgia_guadalupensis_SB729_clonecon TTAT--CAAA-
CAATGAACG-GCACACGT----GCTCGTTCGATTGGGG-GTCGCGTAGCG-----------
----TTGCATTACTAYGCGTCTCTCGAATTGC-------AGTA------CGTTCYGCTTY----

CKGCACGAACAAGGCCTAAAGTGTTTGCCCTTGCCAGTGCG---------------------
---TTAGTGGAGCKCTCTTACCATTCTTAAC
Pterogorgia_citrina_FK4_clonecon TTAT--CAAA-CAATGAACG-
GCACACGT----GCTCGTTCGATTGGGG-GTCGCGTAGCG---------------
TTGCATTACTACGCGTCTCTCGAATTGC-------AGTA------CGTTCCGCTTT----
CTGCACGAACAAGGYCYAAAGTGTTTGCCCTTGYCAGTGCG---------------------
----TTAGTGGAGCTCTCTTACCATTCTTAAC
Pterogorgia_anceps_FK1_clonecon TTAT--CAAA-CAATGAACG-
GCACACGT----GCTCGTTCGATTGGGG-GTCGCGYAGCG---------------
TTGCRTTACTATGCGTCTCTCGAATTGC-------AGTA------CGTTCTGCTTT----
CTGCACGAACAAGGCCTAAAGTGTTTGCCCTTGCCAGTGCG---------------------
---TTAGTGGAGCYCTCTTACCATTCTTAAC
Pterogorgia_citrina_FK1_clonecon TYAT--CAAA-CAATGAACG-
GCACACGT----GCTCGTTCGATTGGGG-GTCGCGTAGCG---------------
TTGCATTACTRCGCGTCTCTCGAATTGC-------AGTA------CGTTCCGCTTT----
CTGCACGAACAAGGCCTAAAGTGTTTGCCCTTGCCAGTGCG---------------------
---TTAGTGGAGCTCTCTTACCATTCTTAAC
Pterogorgia_citrina_FK5_clonecon TTAT--CAAA-CAATGAACG-
GCACACGT----GCTCGTTCGATTRGGG-GTCGCGTAGCG---------------
TTGCATTACTACGCGTCTCTCGAATTGC-------AGTA------CGTTCCGCTTT----
CYGCACGAACAAGGCCTAAAGTGTTTGCCCTTGCCAGTGYG---------------------
----TTAGTGGAGCTCTCTTACCATTCTTAAC
Pterogorgia_citrina_FK6_clonecon TTAT--CAAR-CAATGAACG-
GCACACGT----GCTCGTTCGATTGGGG-GTCGCGTAGCG---------------
TTGCATTACTACGCGTCTCTCGAATTGC-------AGTA------CGTTCCGCTTT----
CTGCACGAACAAGGCCTAAAGTGTTTGCCCTTGCCAGTGCG---------------------
---TTAGTGRAGCTCTCTTACCATTCTTAAC
Pterogorgia_citrina_FK2_clonecon TTAT--CAAA-CAATGAACG-
GCACACGT----GCTCGTTCGATTRGGG-GTCGCGTAGCG---------------
TTGCATTACTACGCGTCTCTCGAATTGC-------AGTA------CGTTCCGCTTT----
CTGCACGAACAAGGCCTAAAGTGTTTGCCCTTGCCAGTGCG---------------------
---TTAGTGGAGCTCTCTTACCATTCTTAAC
Pterogorgia_citrina_SB724_clonecon TTAT--CAAA-CAATGAACG-
GCACACGT----GCTCGTTCGATTGGGG-GTCGCGTAGCG---------------
TTGCATTACTACGCGTCTCTCGAATTGC-------AGTA------CGTTCCGCTTT----
CTGCACGAACAAGGCCTAAAGTGYTTTGCCCTTGCCAGTGCG---------------------
---TTAGTGGAGCTCTCTTACCATTCTTAAC
Pterogorgia_citrina_SB722_clonecon TTAT--CAAACCAATGAACG-
GCACACGT----GCTCGTTCGATTGGGG-GTCGCGTAGCG---------------
TTGCATTACTACGCGTCTCTCGAATTGC-------AGTA------CGTTCCGCTTT----
CTGCACGAACAAGGCCTAAAGTGTTTGCCCTYGCCAGTGCG---------------------
---TTAGTGGAGCTCTCTTACCATTCTTAAC
Pterogorgia_citrina_FK3_clonecon TTAT--CAAA-CAATGAACG-
GCACACGT----GCTCGTTCGATTGGGG-GTCGCGTAGCG---------------
TTGCATTACTACGCGTCTCTCGAATTGC-------AGTA------CGTTCCGCTTT----

CTGCACGAACAAGGCCTAAAGTGTTTGCCCTTGCCAGTGCG---------------------
---TTAGTGGAGCTCTCTTACCATTCTTAAC

Pterogorgia_sp_SB2_clonecon TTAT--CAAA-CAATGAACG-
GCACACGT----GCTCGTTCGATTGGGG-GTCGCGTAGCG--------------
TTGCATTACTACGCGTCTCTCGAATTGC-------AGTA------CGTTCCGCTTT----
CTGCACGAACAAGGCCTAAAGTGTTTGCCCTTGCCAGTGCG---------------------
---TTAGTGGAGCTCTCTTACCATTCTTAAC

Pterogorgia_sp_SB1_clonecon TTAT--CAAACCAATGAACG-
GCACACGT----GCTCGTTCGATTGGGG-GTCGCGTAGCG--------------
TTGCATTACTACGCGTCTCTCGAATTGC-------AGTR------CGTTCCGCTTT----
CTGCACGAACAAGGCCTAAAGTGTTTGCCCTTGCCAGTGCG---------------------
---TTAGTGGAGCTCTCTTACCATTCTTAAC

Pterogorgia_anceps_cf_SB721_clonecon TTAT--CAAA-CAATGAACG-
GCACACGT----GCTCGTTCGATTGGGG-GTCGCGTAGCG--------------
TTGCATTACTAYGCGYCTCTCGAATTGC-------AGTA------CGTTCYGCTTT----
CTGCACGAACAAGGCCTAAAGTGTTTGCCCTTGCCAGTGCG---------------------
---TTAGTGGAGCTCTCTTACCATTCTTAAC

Pterogorgia_citrina_SB723_clonecon TTAT--CAAA-CAATGAACG-
GCACACGT----GCTCGTTCGATTGGGG-GTCGCGTAGCG--------------
TTGCATTACTAYGCGTCTCTCGAATTGC-------AGTA------CGTTCYGCTTT----
CTGCACGAACAAGGCCTAAAGTGTTTGCCCTTGCCAGTGCG---------------------
---TTAGTGGAGCTCTCTTACCATTCTTAAC

Pterogorgia_guadalupensis_SB727_clonecon TTAT--CAAA-
CAATGAACG-GCACACGT----GCTCGTTCGATTGGGG-GTCGCGTAGCG-----------
----TTGCATTACTATGCGTCTCTCGAATTGC-------AGTA------CGTTCTGCYTT----
CTGCACGAACAAGGCCTAAAGTGTTTGCCCTTGCCAGTGCG---------------------
---TTAGTGGAGCTCTCTTAYCATTCTTAAC

Pterogorgia_anceps_FK3_clonecon TTAT--CAAA-CAATGAACG-
GCACACGT----GCTCGTTCGRTTGGGG-GTCGCGTAGCG--------------
TTGCATTACTATGCGTCTCTCGAATTGC-------AGTA------CGTTCTGCTTT----
CTGCACGAACAAGGCCTAAAGTGTTTGCCCTTGCCAGTGCG---------------------
---TTAGTGGAGCTCTCTTACCATTCTTAAC

Pterogorgia_anceps_FK4_clonecon TTAT--CAAA-CAATGAACG-
GCACACGT----GCTCGTTCGATTGGGG-GTCGCGTAGCG--------------
TTGCATYACTATGCGTCTCTCGAATTGC-------AGTA------CGTTCTGCTTT----
CTGCACGAACAAGGCCTAAAGTGTTTGCCCTTGCCAGTGCG---------------------
---TTAGTGGAGCTCTCTTACCATTCTTAAC

Pterogorgia_anceps_FLlr6_clonecon TTAT--CAAA-CAATGAACG-
GCACACGT----GCTCGTTCGATTGGGG-GTCGCGTAGCG--------------
TTGCATTACTATGCGTCTCTCGAATTGC-------AGTA------CGTTCTGCTTT----
CTGCACGAACAAGGCCTAAAGTGTTTGCCCTTGCCAGTGCG---------------------
---TTAGTGGRGCTCTCTTACCATTCTTAAC

Pterogorgia_anceps_FK2_clonecon TTAT--CAAA-CAATGAACG-
GCACACGT----GCTCGTTCGATTGGGG-GTCGCGTAGCG--------------
TTGCATTACTATGCGTCTCTCGAATTGC-------AGTA------CGTTCTGCTTT----

CTGCACGAACAAGGCCTAAAGTGTTTGCCCTTGCCAGTGCG---------------------
---TTAGTGGAGCTCTCTTACCATTCTTAAC
Pterogorgia_anceps_FK5_clonecon TTAT--CAAA-CAATGAACG-
GCACACGT----GCTCGTTCGATTGGGG-GTCGCGTAGCG--------------
TTGCATTACTATGCGTCTCTCGAATTGC-------AGTA------CGTTCTGCTTT----
CTGCACGAACAAGGCCTAAAGTGTTTGCCCTTGCCAGTGCG---------------------
---TTAGTGGAGCTCTCTTACCATTCTTAAC
Pterogorgia_guadalupensis_SB726_clonecon TTAT--CAAA-
CAATGAACG-GCACACGT----GCTCGTTCGATTGGGG-GTCGCGTAGCG-----------
----TTGCATTACTATGCGTCTCTCGAATTGC-------AGTA------CGTTCTGCTTT----
CTGCACGAACAAGGCCTAAAGTGTTTGCCCTTGCCAGTGCG---------------------
---TTAGTGGAGCTCTCTTACCATTCTTAAC
Pterogorgia_guadalupensis_SB725_clonecon TTAT--CAAA-
CAATGAACG-GCACACGT----GCTCGTTCGATTGGGG-GTCGCGTAGCG-----------
----TTGCATTACTATGCGTCTCTCGAATTGC-------AGTA------CGTTCTGCTTT----
CTGCACGAACAAGGCCTAAAGTGTTTYGCCCTTGCCAGTGCG---------------------
---TTAGTGGAGCTCTCTTACCATTCTTAAC

;
end;

(3) *SRP54* unique

#NEXUS
begin taxa;
dimensions ntax=49;
taxlabels
'Pterogorgia_anceps_FK1_c2/2'[&description=""]
'Pterogorgia_anceps_FK3_c3/4'[&description=""]
'Pterogorgia_anceps_FK3_c1/4'[&description=""]
'Pterogorgia_anceps_FK4_c1/2'[&description=""]
'Pterogorgia_guadalupensis_SB729_c1/1'[&description=""]
'Pterogorgia_guadalupensis_SB728_c3/3'[&description=""]
'Pterogorgia_guadalupensis_SB728_c2/3'[&description=""]
'Pterogorgia_anceps_FK2_c4/4'[&description=""]
'Pterogorgia_anceps_cf_SB721_c2/3'[&description=""]
'Pterogorgia_anceps_FK2_c3/4'[&description=""]
'Pterogorgia_anceps_FK2_c2/4'[&description=""]
'Pterogorgia_anceps_cf_SB721_c3/3'[&description=""]
'Pterogorgia_guadalupensis_SB728_c1/3'[&description=""]
'Pterogorgia_guadalupensis_SB726_c1/1'[&description=""]
'Pterogorgia_anceps_FK6_c1/1'[&description=""]
'Pterogorgia_anceps_FK5_c1/1'[&description=""]
'Pterogorgia_anceps_FK4_c2/2'[&description=""]

'Pterogorgia_anceps_FK3_c4/4'[&description=""]
'Pterogorgia_anceps_FK2_c1/4'[&description=""]
'Pterogorgia_anceps_cf_SB721_c1/3'[&description=""]
'Pterogorgia_anceps_FK3_c2/4'[&description=""]
'Pterogorgia_anceps_FK1_c1/2'[&description=""]
'Pterogorgia_citrina_SB723_c2/3'[&description=""]
'Pterogorgia_sp_SB1_c3/3'[&description=""]
'Pterogorgia_sp_SB1_c1/3'[&description=""]
'Pterogorgia_guadalupensis_SB727_c3/3'[&description=""]
'Pterogorgia_guadalupensis_SB727_c1/3'[&description=""]
'Pterogorgia_guadalupensis_SB725_c2/2'[&description=""]
'Pterogorgia_citrina_SB723_c3/3'[&description=""]
'Pterogorgia_citrina_FK2_c1/2'[&description=""]
'Pterogorgia_citrina_FK1_c1/2'[&description=""]
'Pterogorgia_sp_SB2_c3/3'[&description=""]
'Pterogorgia_sp_SB1_c2/3'[&description=""]
'Pterogorgia_guadalupensis_SB727_c2/3'[&description=""]
'Pterogorgia_guadalupensis_SB725_c1/2'[&description=""]
'Pterogorgia_citrina_SB724_c1/1'[&description=""]
'Pterogorgia_citrina_SB723_c1/3'[&description=""]
'Pterogorgia_citrina_SB722_c1/1'[&description=""]
'Pterogorgia_citrina_FK6_c1/1'[&description=""]
'Pterogorgia_citrina_FK5_c1/1'[&description=""]
'Pterogorgia_citrina_FK4_c1/1'[&description=""]
'Pterogorgia_citrina_FK3_c1/1'[&description=""]
'Pterogorgia_citrina_FK2_c2/2'[&description=""]
'Pterogorgia_citrina_FK1_c2/2'[&description=""]
'Pterogorgia_sp_SB2_c2/3'[&description=""]
'Pterogorgia_sp_SB2_c1/3'[&description=""]
Pseudopterogorgia_acerosa[&description=""]
Plexaura_flexuosa[&description=""]
Gorgonia_ventalina[&description=""]
;
end;

begin characters;
dimensions nchar=189;
format datatype=dna missing=? gap=-;
matrix
'Pterogorgia_anceps_FK1_c2/2'
TGAACTAAAATTAGAAGAAAATGAAGAACTAATAGGAAAATTAAAAC
ATGGTAAGTAAAT-----ACTTGATCGT---------TG------ACAAGTA-AAGT----AGA--
----------GAGATAACTTA------CAAGAATTAC-------ATTGATTTTATT-
AGGACAATTTACGCTAAGG
'Pterogorgia_anceps_FK3_c3/4'
TGAACTAAAATTAGAAGAAAATGAAGGACTAATAGGAAAATTAAAAC

ATGGTAAGTAAAT-----ACTTGATCGT---------TG------ACAAGTA-AAGT----AGA--
----------GAGATAACTTA------CAAGAATTAC-------ATTGATTTTATT-
AGGACAATTTACGCTAAGA

'Pterogorgia_anceps_FK3_c1/4'

TGAACTAAAATTAGAAGAAAATGAAGAACTAATAGGAAAATTAAAAC
ATGGTAAGTAAAT-----ACTTGATCGT---------TG------ACAAGTA-AAGT----AGA--
----------GAGGTAACTTA------CAAGAATTAC-------ATTGATTTTATT-
AGGACAATTTACGCTAAGA

'Pterogorgia_anceps_FK4_c1/2'

TGAACTAAAATTAGAAGAAAATGAAGAACTAATAGGAAAATTAAAAC
ATGGTAAGTAAAT-----ACTTGATCGT---------TG------ACAAGTA-AAGT----AGA--
----------GGGATAACTTA------CAAGAATTAC----ATCATTGATTTTATT-
AGGACAATTTACGCTAAGA

'Pterogorgia_guadalupensis_SB729_c1/1'

TGAACTAAAATTAGAAGAAAATGAAGAACTAATAGGAAAATTAAAAC
ATGGTAAGTAAAT-----ACTTGATCGT---------TG------ACAAGTA-AACT----AGA--
----------GAGATAACTTA------CAAGAATTAC----ATTATTGATTTTATT-
AGGACAATTTACGCTAAGA

'Pterogorgia_guadalupensis_SB728_c3/3'

TGAACTAAAATTAGAAGAAAATGAAGAACTAATAGGAAAATTAAAAC
ATGGTAAGTAAGT-----ACTTGATCGT---------TG------ACAAGTA-AAGT----AGA--
----------GAGATAACTTA------CAAGAATTAC----ATTATTGATTTTATT-
AGGACAATTTACGCTAAGA

'Pterogorgia_guadalupensis_SB728_c2/3'

TGAACTAAAATTAGAAGAAAATGAAGAACTAATAGGAAAATTAAAAC
ATGGTAAGTAAAT-----ACTTGATCGT---------TG------ACAAGTA-AAGT----AGA--
----------GAGATAACTTA------CAAGAATTAC----ATTATTGATTTTATT-
TGGACAATTTACGCTAAGA

'Pterogorgia_anceps_FK2_c4/4'

TGAACTAAAATTAGAAGAAAATGAAGAACTAATAGGAAGATTAAAAC
ATGGTAAGTAAAT-----ACTTGATCGT---------TG------ACAAGTA-AAGT----AGA--
----------GAGATAACTTA------CAAGAATTAC----ATTATTGATTTTATT-
AGGACAATTTACGCTAAGA

'Pterogorgia_anceps_cf_SB721_c2/3'

TGAACTAAAATTAGAAGAAAATGAAGAACTAATAGGAAGATTAAAAC
ATGGTAAGTAAAT-----ACTTGATCGT---------TG------ACAAGTA-AAGT----AGA--
----------GAGATAACTTA------CAAGAATTAC----ATTATTGATTTTATT-
AGGACAATTTACGCTAAGA

'Pterogorgia_anceps_FK2_c3/4'

TGAACTAAAATTAGAAGAAAATGAAGAACTAATAGGAAAATTAAAAC
ATGGTAAGTAAAT-----ACTTGATCGT---------TG------ACAAGTA-AAGT----AGA--
----------GAGATAACTTA------CTAGAATTAC----ATTATTGATTTTATT-
AGGACAATTTACGCTAAGA

'Pterogorgia_anceps_FK2_c2/4'

TGAACTAAAATTAGAAGAAAATGAAGAACTAATAGGAAAATTAAAAC
ATGGTAAGTAAAT-----ACTTGATCGT---------TG------ACAAGTA-AAGT----AGA--

----------GAGATAACTCA------CAAGAATTAC----ATTATTGATTTTATT-AGGACAATTTACGCTAAGA
'Pterogorgia_anceps_cf_SB721_c3/3'
TGAACTAAAATTAGAAGAAAATGAAGAACTAATAGGAAAATTAAAAC ATGGTAAGTAAAT-----ACTTGATCGT---------TG------ACAAGTA-AAGT----AGA------------GAGATAACTTA------CAAGAATTAG----ATTATTGATTTTATT-AGGACAATTTACGCTAAGA
'Pterogorgia_guadalupensis_SB728_c1/3'
TGAACTAAAATTAGAAGAAAATGAAGAACTAATAGGAAAATTAAAAC ATGGTAAGTAAAT-----ACTTGATCGT---------TG------ACAAGTA-AAGT----AGA------------GAGATAACTTA------CAAGAATTAC----ATTATTGATTTTATT-AGGACAATTTACGCTAAGA
'Pterogorgia_guadalupensis_SB726_c1/1'
TGAACTAAAATTAGAAGAAAATGAAGAACTAATAGGAAAATTAAAAC ATGGTAAGTAAAT-----ACTTGATCGT---------TG------ACAAGTA-AAGT----AGA------------GAGATAACTTA------CAAGAATTAC----ATTATTGATTTTATT-AGGACAATTTACGCTAAGA
'Pterogorgia_anceps_FK6_c1/1'
TGAACTAAAATTAGAAGAAAATGAAGAACTAATAGGAAAATTAAAAC ATGGTAAGTAAAT-----ACTTGATCGT---------TG------ACAAGTA-AAGT----AGA------------GAGATAACTTA------CAAGAATTAC----ATTATTGATTTTATT-AGGACAATTTACGCTAAGA
'Pterogorgia_anceps_FK5_c1/1'
TGAACTAAAATTAGAAGAAAATGAAGAACTAATAGGAAAATTAAAAC ATGGTAAGTAAAT-----ACTTGATCGT---------TG------ACAAGTA-AAGT----AGA------------GAGATAACTTA------CAAGAATTAC----ATTATTGATTTTATT-AGGACAATTTACGCTAAGA
'Pterogorgia_anceps_FK4_c2/2'
TGAACTAAAATTAGAAGAAAATGAAGAACTAATAGGAAAATTAAAAC ATGGTAAGTAAAT-----ACTTGATCGT---------TG------ACAAGTA-AAGT----AGA------------GAGATAACTTA------CAAGAATTAC----ATTATTGATTTTATT-AGGACAATTTACGCTAAGA
'Pterogorgia_anceps_FK3_c4/4'
TGAACTAAAATTAGAAGAAAATGAAGAACTAATAGGAAAATTAAAAC ATGGTAAGTAAAT-----ACTTGATCGT---------TG------ACAAGTA-AAGT----AGA------------GAGATAACTTA------CAAGAATTAC----ATTATTGATTTTATT-AGGACAATTTACGCTAAGA
'Pterogorgia_anceps_FK2_c1/4'
TGAACTAAAATTAGAAGAAAATGAAGAACTAATAGGAAAATTAAAAC ATGGTAAGTAAAT-----ACTTGATCGT---------TG------ACAAGTA-AAGT----AGA------------GAGATAACTTA------CAAGAATTAC----ATTATTGATTTTATT-AGGACAATTTACGCTAAGA
'Pterogorgia_anceps_cf_SB721_c1/3'
TGAACTAAAATTAGAAGAAAATGAAGAACTAATAGGAAAATTAAAAC ATGGTAAGTAAAT-----ACTTGATCGT---------TG------ACAAGTA-AAGT----AGA--

----------GAGATAACTTA------CAAGAATTAC----ATTATTGATTTTATT-AGGACAATTTACGCTAAGA

'Pterogorgia_anceps_FK3_c2/4'

TGAACTAAAATTAGAAGAAAATGAAGAACTAATAGGAAAATTAAAAC ATGGTAAGTAAAT-----ACTTGATCGT---------TG------ACAAGTA-AAGT----AGA------------GAGATAACTTA------CAAGAATTAC-------ATTGATTTTATT-AGGACAATTTACGCTAAGA

'Pterogorgia_anceps_FK1_c1/2'

TGAACTAAAATTAGAAGAAAATGAAGAACTAATAGGAAAATTAAAAC ATGGTAAGTAAAT-----ACTTGATCGT---------TG------ACAAGTA-AAGT----AGA------------GAGATAACTTA------CAAGAATTAC-------ATTGATTTTATT-AGGACAATTTACGCTAAGA

'Pterogorgia_citrina_SB723_c2/3'

TGAATTAAAATTAGAAGGAAATGAAGAACTAATAGGAAGATTAAAAC ATGGTAAGTAAAT-----ACTTGATCGT---------TG------ACAAGTA-AAAT----AGAGA----------GAGATAACTTA------CAGTAATTAT----ATTATTGATTTTATT-AGGACAATTTACGCTAAGA

'Pterogorgia_sp_SB1_c3/3'

TGAATTAAAATTAGAAGAAAATGAAGAACTAATAGGAAAATTAAAAC ATGGTAAGTAAAT-----ACTTGATCGT---------TG------ACAAGTA-AAAT----AGAGA----------GAGATAACTTA------CAGTAATTAT----ATTATTGATTTAATT-AGGACAATTTACGCTAAGA

'Pterogorgia_sp_SB1_c1/3'

TGAATTAAAATTAGAAGAAAATGGAGAACTAATAGGAAAATTAAAAC ATGGTAAGTAAAT-----ACTTGATCGT---------TG------ACAAGTA-AAAT----AGAGA----------GAGATAACTTA------CAGTAATTAT----ATTATTGATTTTATT-AGGACAATTTACGCTAAGA

'Pterogorgia_guadalupensis_SB727_c3/3'

TGAATTGAAATTAGAAGAAAATGAAGAACTAATAGGAAAATTAAAAC ATGGTAAGTAAAT-----ACTTGATCGT---------TG------ACAAGTA-AAAT----AGAGA----------GAGATAACTTA------CAGTAATTAT----ATTATTGATTTTATT-AGGACAATTTACGCTAAGA

'Pterogorgia_guadalupensis_SB727_c1/3'

TGAATTAAAATTAGAAGAGAATGAAGAACTAATAGGAAAATTAAAAC ATGGTAAGTAAAT-----ACTTGATCGT---------TG------ACAAGTA-AAAT----AGAGA----------GAGATAACTTA------CAGTAATTAT----ATTATTGATTTTATT-AGGACAATTTACGCTAAGA

'Pterogorgia_guadalupensis_SB725_c2/2'

TGAATTAAAATTAGAAGAAAATGAAGAACTAATAGGAAAATTAAAAC ATGGTAAGTAAAT-----ACTTGATCGT---------TG------ACAAGTA-AAAT----AGAGG----------GAGATAACTTA------CAGTAATTAT----ATTATTGATTTTATT-AGGACAATTTACGCTAAGA

'Pterogorgia_citrina_SB723_c3/3'

TGAATTAAAATTAGAAGAAAATGAGGAACTAATAGGAAAATTAAAAC ATGGTAAGTAAAT-----ACTTGATCGT---------TG------ACAAGTA-AAAT----

AGAGA----------GAGATAACTTA------CAGTAATTAT----ATTATTGATTTTATT-AGGACAATTTACGCTAAGA

'Pterogorgia_citrina_FK2_c1/2'

TGAATTAAAGTTAGAAGAAAATGAAGAACTAATAGGAAAATTAAAACATGGTAAGTAAAT-----ACTTGATCGT---------TG------ACAAGTA-AAAT----AGAGA----------GAGATAACTTA------CAGTAATTAT----ATTATTGATTTTATT-AGGACAATTTACGCTAAGA

'Pterogorgia_citrina_FK1_c1/2'

TGAATTAAAATTAGAAGAAAATGAAGAACTAATAGGAAAATTAAAACATGGTAAGTAAAT-----ACTTGATCGT---------TG------ACAAGTA-AAAT----AGAAA----------GAGATAACTTA------CAGTAATTAT----ATTATTGATTTTATT-AGGACAATTTACGCTAAGA

'Pterogorgia_sp_SB2_c3/3'

TGAATTAAAATTAGAAGAAAATGAAGAACTAATAGGAAAATTAAAACATGGTAAGTAAAT-----ACTTGATCGT---------TG------ACAAGTA-AAAT----AGAGA----------GAGATAACTTA------CAGTAATTAT----ATTATTGATTTTATT-AGGACAATTTACGCTAAGA

'Pterogorgia_sp_SB1_c2/3'

TGAATTAAAATTAGAAGAAAATGAAGAACTAATAGGAAAATTAAAACATGGTAAGTAAAT-----ACTTGATCGT---------TG------ACAAGTA-AAAT----AGAGA----------GAGATAACTTA------CAGTAATTAT----ATTATTGATTTTATT-AGGACAATTTACGCTAAGA

'Pterogorgia_guadalupensis_SB727_c2/3'

TGAATTAAAATTAGAAGAAAATGAAGAACTAATAGGAAAATTAAAACATGGTAAGTAAAT-----ACTTGATCGT---------TG------ACAAGTA-AAAT----AGAGA----------GAGATAACTTA------CAGTAATTAT----ATTATTGATTTTATT-AGGACAATTTACGCTAAGA

'Pterogorgia_guadalupensis_SB725_c1/2'

TGAATTAAAATTAGAAGAAAATGAAGAACTAATAGGAAAATTAAAACATGGTAAGTAAAT-----ACTTGATCGT---------TG------ACAAGTA-AAAT----AGAGA----------GAGATAACTTA------CAGTAATTAT----ATTATTGATTTTATT-AGGACAATTTACGCTAAGA

'Pterogorgia_citrina_SB724_c1/1'

TGAATTAAAATTAGAAGAAAATGAAGAACTAATAGGAAAATTAAAACATGGTAAGTAAAT-----ACTTGATCGT---------TG------ACAAGTA-AAAT----AGAGA----------GAGATAACTTA------CAGTAATTAT----ATTATTGATTTTATT-AGGACAATTTACGCTAAGA

'Pterogorgia_citrina_SB723_c1/3'

TGAATTAAAATTAGAAGAAAATGAAGAACTAATAGGAAAATTAAAACATGGTAAGTAAAT-----ACTTGATCGT---------TG------ACAAGTA-AAAT----AGAGA----------GAGATAACTTA------CAGTAATTAT----ATTATTGATTTTATT-AGGACAATTTACGCTAAGA

'Pterogorgia_citrina_SB722_c1/1'

TGAATTAAAATTAGAAGAAAATGAAGAACTAATAGGAAAATTAAAACATGGTAAGTAAAT-----ACTTGATCGT---------TG------ACAAGTA-AAAT----

AGAGA----------GAGATAACTTA------CAGTAATTAT----ATTATTGATTTTATT-AGGACAATTTACGCTAAGA

'Pterogorgia_citrina_FK6_c1/1'

TGAATTAAAATTAGAAGAAAATGAAGAACTAATAGGAAAATTAAAAC ATGGTAAGTAAAT-----ACTTGATCGT---------TG------ACAAGTA-AAAT----AGAGA----------GAGATAACTTA------CAGTAATTAT----ATTATTGATTTTATT-AGGACAATTTACGCTAAGA

'Pterogorgia_citrina_FK5_c1/1'

TGAATTAAAATTAGAAGAAAATGAAGAACTAATAGGAAAATTAAAAC ATGGTAAGTAAAT-----ACTTGATCGT---------TG------ACAAGTA-AAAT----AGAGA----------GAGATAACTTA------CAGTAATTAT----ATTATTGATTTTATT-AGGACAATTTACGCTAAGA

'Pterogorgia_citrina_FK4_c1/1'

TGAATTAAAATTAGAAGAAAATGAAGAACTAATAGGAAAATTAAAAC ATGGTAAGTAAAT-----ACTTGATCGT---------TG------ACAAGTA-AAAT----AGAGA----------GAGATAACTTA------CAGTAATTAT----ATTATTGATTTTATT-AGGACAATTTACGCTAAGA

'Pterogorgia_citrina_FK3_c1/1'

TGAATTAAAATTAGAAGAAAATGAAGAACTAATAGGAAAATTAAAAC ATGGTAAGTAAAT-----ACTTGATCGT---------TG------ACAAGTA-AAAT----AGAGA----------GAGATAACTTA------CAGTAATTAT----ATTATTGATTTTATT-AGGACAATTTACGCTAAGA

'Pterogorgia_citrina_FK2_c2/2'

TGAATTAAAATTAGAAGAAAATGAAGAACTAATAGGAAAATTAAAAC ATGGTAAGTAAAT-----ACTTGATCGT---------TG------ACAAGTA-AAAT----AGAGA----------GAGATAACTTA------CAGTAATTAT----ATTATTGATTTTATT-AGGACAATTTACGCTAAGA

'Pterogorgia_citrina_FK1_c2/2'

TGAATTAAAATTAGAAGAAAATGAAGAACTAATAGGAAAATTAAAAC ATGGTAAGTAAAT-----ACTTGATCGT---------TG------ACAAGTA-AAAT----AGAGA----------GAGATAACTTA------CAGTAATTAT----ATTATTGATTTTATT-AGGACAATTTACGCTAAGA

'Pterogorgia_sp_SB2_c2/3'

TGAATTAAAATTAGAAGAAAATGAAGAACTAATAGGAAAATTAAAAC ATGGTAAGTAAAT-----ACTTGATCGT---------TG------ACAAGTA-AAAT----AGAGA----------GAGATAACTTA------CGGTAATTAT----ATAATTGATTTTATT-AGGACAATTTACGCTAAGA

'Pterogorgia_sp_SB2_c1/3'

TGAATTAAAATTAGAAGAAAATGAAGAACTAATAGGAAAATTAAAAC ATGGTAAGTAAAT-----ACTTGATCGT---------TG------ACAAGTA-AAAT----AGAGA----------GAGATAACTTA------CAGTAATTAT----ATAATTGATTTTATT-AGGACAATTTACGCTAAGA

Pseudopterogorgia_acerosa

TGARCTAAAATTGGAAGAGAATGAAGAACTAATAGGAAAATTAAAAC ATGGTAGGTTTTT-----ATTTGATTTCT------TTGCACTGTACATGT-GTAAT----

TGTAATATTTTTNTAMGAAAGCCTAGCATGTTATTAACCATTGACGTGTTTG
ATTTTATTTAGGCCAGTTTACTTTGAGA
Plexaura_flexuosa
CGAACTTRAATTAGAAGAAAACGAAGAATTAATAGGAAAATTAAAAC
ATGGTAGGTGAAT----GGTGTGATTGTATAGAGAATAGGATTCT-
CATTTAGCAATGAAATGACAAAGGAGCATTCTGARCACTGAAT-
GGCAAGTGA--AT-GACGTTGCGTCTTTTTTTCAGGTCAGTTCACACTGAGA
Gorgonia_ventalina
TGAACTAAAATTGGAAGAAAATGAAGAACTAATAGGAAAATTGAAAC
ATGGTAGGTTTTTAATAGATTTGATTTTTT------TTGCACTGTACRTGT-GTACT-

TGTAATAYTTTTTGTACTAAAGCCAAATATGCTAGTGATCATTGACATGTTTG
ATTTTATT-AGGCCAGTTTACTTTGAGG

;
end;

(4) *SRP54* consensus

#NEXUS
begin taxa;
dimensions ntax=26;
taxlabels
Pterogorgia_guadalupensis_SB729_clonecon[&description=""]
Pterogorgia_anceps_FLR_clonecon[&description=""]
Pterogorgia_anceps5_KL_clonecon[&description=""]
Pterogorgia_guadalupensis_SB726_clonecon[&description=""]
Pterogorgia_guadalupensis_SB728_clonecon[&description=""]
Pterogorgia_anceps4_KL_clonescon[&description=""]
Pterogorgia_ancepsCF_SB721_clonecon[&description=""]
Pterogorgia_anceps2_KL_clonecon[&description=""]
Pterogorgia_anceps1_KL_clonecon[&description=""]
Pterogorgia_anceps3_KL_clonecon[&description=""]
Pterogorgia_guadalupensis_SB725_clonecon[&description=""]
Pterogorgia_guadalupensis_SB727_clonecon[&description=""]
Pterogorgia_citrina6_KL_clonecon[&description=""]
Pterogorgia_citrina4_KL_clonecon[&description=""]
Pterogorgia_citrina_SB722_clonecon[&description=""]
Pterogorgia_citrina3_KL_clonecon[&description=""]
Pterogorgia_citrina5_KL_clonecon[&description=""]
Pterogorgia_citrina2_KL_clonecon[&description=""]
Pterogorgia_sp_SB2_clonecon[&description=""]
Pterogorgia_citrina1_KL_clonecon[&description=""]
Pterogorgia_citrina_SB723_clonecon[&description=""]

Pterogorgia_citrina_SB724_clonecon[&description=""]
Pterogorgia_sp_SB1_clonecon[&description=""]
Gorgonia_ventalina_KW_clonecon[&description=""]
Pseudopterogorgia_acerosa_KW_clonecon[&description=""]
Plexaura_flexuosa_FR1_clonecon[&description=""]
;
end;

begin characters;
dimensions nchar=190;
format datatype=dna missing=? gap=-;
matrix
Pterogorgia_guadalupensis_SB729_clonecon
TGAACTAAAATTAGAAGAAAATGAAGAACTAATAGGAAAATTAAAAC
ATGGTAAGTAAAT-----ACTTGATCGT---------------------TGACAA--------
GTAAASTAGA--GAGATAACTTACAAG------------AATTACATTATTGATTTTA-
TTAGGACAATTTACGCTAAGA
Pterogorgia_anceps_FLR_clonecon
TGAACTAAAATTAGAAGAAAATGAAGAACTAATAGGAAAATTAAAAC
ATGGTAAGTAAAT-----ACTTGATCGT---------------------TGACAA--------
GTAAAGTAGA--GAGATAACTTACAAG------------AATTACATTATTGATTTTA-
TTAGGACAATTTACGCTAAGA
Pterogorgia_anceps5_KL_clonecon
TGAACTAAAATTAGAAGAAAATGAAGAACTAATAGGAAAATTAAAAC
ATGGTAAGTAAAT-----ACTTGATCGT---------------------TGACAA--------
GTAAAGTAGA--GAGATAACTTACAAG------------AATTACATTATTGATTTTA-
TTAGGACAATTTACGCTAAGA
Pterogorgia_guadalupensis_SB726_clonecon
TGAACTAAAATTAGAAGAAAATGAAGAACTAATAGGAAAATTAAAAC
ATGGTAAGTAAAT-----ACTTGATCGT---------------------TGACAA--------
GTAAAGTAGA--GAGATAACTTACAAG------------AATTACATTATTGATTTTA-
TTAGGACAATTTACGCTAAGA
Pterogorgia_guadalupensis_SB728_clonecon
TGAACTAAAATTAGAAGAAAATGAAGAACTAATAGGAAAATTAAAAC
ATGGTAAGTAART-----ACTTGATCGT---------------------TGACAA--------
GTAAAGTAGA--GAGATAACTTACAAG------------AATTACATTATTGATTTTA-
TTWGGACAATTTACGCTAAGA
Pterogorgia_anceps4_KL_clonescon
TGAACTAAAATTAGAAGAAAATGAAGAACTAATAGGAAAATTAAAAC
ATGGTAAGTAAAT-----ACTTGATCGT---------------------TGACAA--------
GTAAAGTAGA--GRGATAACTTACAAG------------AATTACATYATTGATTTTA-
TTAGGACAATTTACGCTAAGA
Pterogorgia_ancepsCF_SB721_clonecon
TGAACTAAAATTAGAAGAAAATGAAGAACTAATAGGAARATTAAAAC
ATGGTAAGTAAAT-----ACTTGATCGT---------------------TGACAA--------

GTAAAGTAGA--GAGATAACTTACAAG------------AATTASATTATTGATTTTA-TTAGGACAATTTACGCTAAGA

Pterogorgia_anceps2_KL_clonecon

TGAACTAAAATTAGAAGAAAATGAAGAACTAATAGGAARATTAAAACATGGTAAGTAAAT-----ACTTGATCGT---------------------TGACAA--------GTAAAGTAGA--GAGATAACTYACWAG------------AATTACATTATTGATTTTA-TTAGGACAATTTACGCTAAGA

Pterogorgia_anceps1_KL_clonecon

TGAACTAAAATTAGAAGAAAATGAAGAACTAATAGGAAAATTAAAACATGGTAAGTAAAT-----ACTTGATCGT---------------------TGACAA--------GTAAAGTAGA--GAGATAACTTACAAG------------AATTAC---ATTGATTTTA-TTAGGACAATTTACGCTAAGR

Pterogorgia_anceps3_KL_clonecon

TGAACTAAAATTAGAAGAAAATGAAGRACTAATAGGAAAATTAAAACATGGTAAGTAAAT-----ACTTGATCGT---------------------TGACAA--------GTAAAGTAGA--GAGRTAACTTACAAG------------AATTAC---ATTGATTTTA-TTAGGACAATTTACGCTAAGA

Pterogorgia_guadalupensis_SB725_clonecon

TGAATTAAAATTAGAAGAAAATGAAGAACTAATAGGAAAATTAAAACATGGTAAGTAAAT-----ACTTGATCGT---------------------TGACAA--------GTAAAATAGAGRGAGATAACTTACAGT------------AATTATATTATTGATTTTA-TTAGGACAATTTACGCTAAGA

Pterogorgia_guadalupensis_SB727_clonecon

TGAATTRAAATTAGAAGARAATGAAGAACTAATAGGAAAATTAAAACATGGTAAGTAAAT-----ACTTGATCGT---------------------TGACAA--------GTAAAATAGAGAGAGATAACTTACAGT------------AATTATATTATTGATTTTA-TTAGGACAATTTACGCTAAGA

Pterogorgia_citrina6_KL_clonecon

TGAATTAAAATTAGAAGAAAATGAAGAACTAATAGGAAAATTAAAACATGGTAAGTAAAT-----ACTTGATCGT---------------------TGACAA--------GTAAAATAGAGAGAGATAACTTACAGT------------AATTATATTATTGATTTTA-TTAGGACAATTTACGCTAAGA

Pterogorgia_citrina4_KL_clonecon

TGAATTAAAATTAGAAGAAAATGAAGAACTAATAGGAAAATTAAAACATGGTAAGTAAAT-----ACTTGATCGT---------------------TGACAA--------GTAAAATAGAGAGAGATAACTTACAGT------------AATTATATTATTGATTTTA-TTAGGACAATTTACGCTAAGA

Pterogorgia_citrina_SB722_clonecon

TGAATTAAAATTAGAAGAAAATGAAGAACTAATAGGAAAATTAAAACATGGTAAGTAAAT-----ACTTGATCGT---------------------TGACAA--------GTAAAATAGAGAGAGATAACTTACAGT------------AATTATATTATTGATTTTA-TTAGGACAATTTACGCTAAGA

Pterogorgia_citrina3_KL_clonecon

TGAATTAAAATTAGAAGAAAATGAAGAACTAATAGGAAAATTAAAACATGGTAAGTAAAT-----ACTTGATCGT---------------------TGACAA--------

GTAAAATAGAGAGAGATAACTTACAGT------------AATTATATTATTGATTTTA-TTAGGACAATTTACGCTAAGA

Pterogorgia_citrina5_KL_clonecon

TGAATTAAAATTAGAAGAAAATGAAGAACTAATAGGAAAATTAAAAC ATGGTAAGTAAAT-----ACTTGATCGT---------------------TGACAA--------GTAAAATAGAGAGAGATAACTTACAGT------------AATTATATTATTGATTTTA-TTAGGACAATTTACGCTAAGA

Pterogorgia_citrina2_KL_clonecon

TGAATTAAARTTAGAAGAAAATGAAGAACTAATAGGAAAATTAAAAC ATGGTAAGTAAAT-----ACTTGATCGT---------------------TGACAA--------GTAAAATAGAGAGAGATAACTTACAGT------------AATTATATTATTGATTTTA-TTAGGACAATTTACGCTAAGA

Pterogorgia_sp_SB2_clonecon

TGAATTAAAATTAGAAGAAAATGAAGAACTAATAGGAAAATTAAAAC ATGGTAAGTAAAT-----ACTTGATCGT---------------------TGACAA--------GTAAAATAGAGAGAGATAACTTACRGT------------AATTATATWATTGATTTTA-TTAGGACAATTTACGCTAAGA

Pterogorgia_citrina1_KL_clonecon

TGAATTAAAATTAGAAGAAAATGAAGAACTAATAGGAAAATTAAAAC ATGGTAAGTAAAT-----ACTTGATCGT---------------------TGACAA--------GTAAAATAGARAGAGATAACTTACAGT------------AATTATATTATTGATTTTA-TTAGGACAATTTACGCTAAGA

Pterogorgia_citrina_SB723_clonecon

TGAATTAAAATTAGAAGRAAATGARGAACTAATAGGAARATTAAAAC ATGGTAAGTAAAT-----ACTTGATCGT---------------------TGACAA--------GTAAAATAGAGAGAGATAACTTACAGT------------AATTATATTATTGATTTTA-TTAGGACAATTTACGCTAAGR

Pterogorgia_citrina_SB724_clonecon

TGAATTAAAATTAGAAGAAAATGAAGAACTAATAGGAAAATTAAAAC ATGGTAAGTAAAT-----ACTTGATCGT---------------------TGACAA--------GTAAAATAGAGAGAGATAACTTACAGT------------AATTATATTATTGAYTTTA-TTAGGACAATTTACGCTAAGA

Pterogorgia_sp_SB1_clonecon

TGAATTAAAATTAGAAGAAAATGRAGAACTAATAGGAAAATTAAAAC ATGGTAAGTAAAT-----ACTTGATCGT---------------------TGACAA--------GTAAAATAGAGAGAGATAACTTACAGT------------AATTATATTATTGATTTWA-TTAGGACAATTTACGCTAAGA

Gorgonia_ventalina_KW_clonecon

TGAACTAAAATTGGAAGAAAATGAAGAACTAATAGGAAAATTGAAAC ATGGTAGGTTTTTAATAGATTTGATTTTTTTGCACTGTACRTGTGTACTTGTA ATAYTTTTGTACTAAAGCCAAATATGCTAGTGATC------------ATTGACATGTTTGATTTTA-TTAGGCCAGTTTACTTTGAGG

Pseudopterogorgia_acerosa_KW_clonecon

TGARCTAAAATTGGAAGAGAATGAAGAACTAATAGGAAAATTAAAAC ATGGTAGGTTTTT-----ATTTGATTTTCTTTGCACTGTACATGTGTAATTGTAATATTTTTTNTAMGAAA

GCCTAGCATGTTATTAACC------------
ATTGACGTGTTTGATTTTATTTAGGCCAGTTTACTTTGAGA
Plexaura_flexuosa_FR1_clonecon
CGAACTTRAATTAGAAGAAAACGAAGAATTAATAGGAAAATTAAAAC
ATGGTAGGTGAAT----
GGTGTGATTGTATAGAGAATAGGATTCTCATTTAGCAATG------
AAATGACAAAGGAGCATTCTGARCACTGAATGGCAAGTGAATGACGTTGCGT
CTTTTTTTCAGGTCAGTTCACACTGAGA

;
end;

(5) Mitochondrial loci (*mtMutS*, cytochrome b, *IGR4*)

#NEXUS
begin taxa;
dimensions ntax=26;
taxlabels
Pterogorgia_sp_SB1[&description=""]
Pterogorgia_sp_SB2[&description=""]
Pterogorgia_citrina_FK1[&description=""]
Pterogorgia_citrina_FK2[&description=""]
Pterogorgia_citrina_FK3[&description=""]
Pterogorgia_citrina_FK4[&description=""]
Pterogorgia_citrina_FK5[&description=""]
Pterogorgia_citrina_FK6[&description=""]
Pterogorgia_citrina_SB722[&description=""]
Pterogorgia_citrina_SB723[&description=""]
Pterogorgia_citrina_SB724[&description=""]
Pterogorgia_anceps_FK1[&description=""]
Pterogorgia_anceps_FK2[&description=""]
Pterogorgia_anceps_FK3[&description=""]
Pterogorgia_anceps_FK4[&description=""]
Pterogorgia_anceps_FK5[&description=""]
Pterogorgia_anceps_FLR[&description=""]
Pterogorgia_ancepsCF_SB721[&description=""]
Pterogorgia_guadalupensis_SB725[&description=""]
Pterogorgia_guadalupensis_SB726[&description=""]
Pterogorgia_guadalupensis_SB728[&description=""]
Pterogorgia_guadalupensis_SB729[&description=""]
Pterogorgia_guadalupensis_SB727_concat[&description=""]
Gorgonian_ventalina_FK[&description=""]
Pseudopterogorgia_acerosa_FK[&description=""]
Plexaura_flexuosa_FR1[&description=""]

;
end;

begin characters;
dimensions nchar=726;
format datatype=dna missing=? gap=-;
matrix
Pterogorgia_sp_SB1
CCAGCTTATCCAAATTGGTAAGTTCTATGAACTTTGGCATGAGCCTGA
TACTCCTAGTAGGCAACAAGTATACTCTCAAGCCGAGTTATTAATTGAATCAT
CCATGCGAAGTGGGCCTTTAGAGGCAACGCCTCCCATTGAACAAGTTGCCTC
GTTACTTGATATGAGAATAACATCGCCTGGTAAAAGATCTTTGCTTCAAATGG
GATTTCCAATTTATTCCCTAACTAATCACTTAAGTACCTTGTTGGATAAAGGT
TGGACTGTTATAGTTATCGATGAATTAGTCACTGGTAAATCCGGGCCAAAAC
AACGTGCAGTATCTCAAGTTTATTCTCCTTGTTGTAATTTAGAGGACTGTTCG
GAATTATCCTATGTGTTATCAATTTATTTTTCTCAAGATGACTTATTAGGTATT
ACTTTATTTTCAGCCATGAATGGGCATAGTATAATGTTTCCTGTCTCTTGGGC
GGACAGAGACAAAGTCGCCCGGTTATTAATCAGCTATCGTATTAGAGAAATA
GTAATTTGGGCAAATTTAGAGGTTGGCTCAGAGATTTTAATTAATAAAATATA
TAATTTATTAATTGGCTGGAATTTATTCCCTTCTGAACCCAATATGCTATTTCT
TAGTATTGGTTCCTTTATTAGGCTGGGTAGAAACCAAATTGCTCCGTATGAAA
TAGTATAAGAAGTAAAGTAGGTCATTGTTGGCCGAAGTGCAAT
Pterogorgia_sp_SB2
CCAGCTTATCCAAATTGGTAAGTTCTATGAACTTTGGCATGAGCCTGA
TACTCCTAGTAGGCAACAAGTATACTCTCAAGCCGAGTTATTAATTGAATCAT
CCATGCGAAGTGGGCCTTTAGAGGCAACGCCTCCCATTGAACAAGTTGCCTC
GTTACTTGATATGAGAATAACATCGCCTGGTAAAAGATCTTTGCTTCAAATGG
GATTTCCAATTTATTCCCTAACTAATCACTTAAGTACCTTGTTGGATAAAGGT
TGGACTGTTATAGTTATCGATGAATTAGTCACTGGTAAATCCGGGCCAAAAC
AACGTGCAGTATCTCAAGTTTATTCTCCTTGTTGTAATTTAGAGGACTGTTCG
GAATTATCCTATGTGTTATCAATTTATTTTTCTCAAGATGACTTATTAGGTATT
ACTTTATTTTCAGCCATGAATGGGCATAGTATAATGTTTCCTGTCTCTTGGGC
GGACAGAGACAAAGTCGCCCGGTTATTAATCAGCTATCGTATTAGAGAAATA
GTAATTTGGGCAAATTTAGAGGTTGGCTCAGAGATTTTAATTAATAAAATATA
TAATTTATTAATTGGCTGGAATTTATTCCCTTCTGAACCCAATATGCTATTTCT
TAGTATTGGTTCCTTTATTAGGCTGGGTAGAAACCAAATTGCTCCGTATGAAA
TAGTATAAGAAGTAAAGTAGGTCATTGTTGGCCGAAGTGCAAT
Pterogorgia_citrina_FK1
CCAGCTTATCCAAATTGGTAAGTTCTATGAACTTTGGCATGAGCCTGA
TACTCCTAGTAGGCAACAAGTATACTCTCAAGCCGAGTTATTAATTGAATCAT
CCATGCGAAGTGGGCCTTTAGAGGCAACGCCTCCCATTGAACAAGTTGCCTC
GTTACTTGATATGAGAATAACATCGCCTGGTAAAAGATCTTTGCTTCAAATGG
GATTTCCAATTTATTCCCTAACTAATCACTTAAGTACCTTGTTGGATAAAGGT
TGGACTGTTATAGTTATCGATGAATTAGTCACTGGTAAATCCGGGCCAAAAC
AACGTGCAGTATCTCAAGTTTATTCTCCTTGTTGTAATTTAGAGGACTGTTCG
GAATTATCCTATGTGTTATCAATTTATTTTTCTCAAGATGACTTATTAGGTATT

ACTTTATTTTCAGCCATGAATGGGCATAGTATAATGTTTCCTGTCTCTTGGGC
GGACAGAGACAAAGTCGCCCGGTTATTAATCAGCTATCGTATTAGAGAAATA
GTAATTTGGGCAAATTTAGAGGTTGGCTCAGAGATTTTAATTAATAAAATATA
TAATTTATTAATTGGCTGGAATTTATTCCCTTCTGAACCCAATATGCTATTTCT
TAGTATTGGTTCCTTTATTAGGCTGGGTAGAAACCAAATTGCTCCGTATGAAA
TAGTATAAGAAGTAAAGTAGGTCATTGTTGGCCGAAGTGCAAT

Pterogorgia_citrina_FK2

CCAGCTTATCCAAATTGGTAAGTTCTATGAACTTTGGCATGAGCCTGA
TACTCCTAGTAGGCAACAAGTATACTCTCAAGCCGAGTTATTAATTGAATCAT
CCATGCGAAGTGGGCCTTTAGAGGCAACGCCTCCCATTGAACAAGTTGCCTC
GTTACTTGATATGAGAATAACATCGCCTGGTAAAAGATCTTTGCTTCAAATGG
GATTTCCAATTTATTCCCTAACTAATCACTTAAGTACCTTGTTGGATAAAGGT
TGGACTGTTATAGTTATCGATGAATTAGTCACTGGTAAATCCGGGCCAAAAC
AACGTGCAGTATCTCAAGTTTATTCTCCTTGTTGTAATTTAGAGGACTGTTCG
GAATTATCCTATGTGTTATCAATTTATTTTTCTCAAGATGACTTATTAGGTATT
ACTTTATTTTCAGCCATGAATGGGCATAGTATAATGTTTCCTGTCTCTTGGGC
GGACAGAGACAAAGTCGCCCGGTTATTAATCAGCTATCGTATTAGAGAAATA
GTAATTTGGGCAAATTTAGAGGTTGGCTCAGAGATTTTAATTAATAAAATATA
TAATTTATTAATTGGCTGGAATTTATTCCCTTCTGAACCCAATATGCTATTTCT
TAGTATTGGTTCCTTTATTAGGCTGGGTAGAAACCAAATTGCTCCGTATGAAA
TAGTATAAGAAGTAAAGTAGGTCATTGTTGGCCGAAGTGCAAT

Pterogorgia_citrina_FK3

CCAGCTTATCCAAATTGGTAAGTTCTATGAACTTTGGCATGAGCCTGA
TACTCCTAGTAGGCAACAAGTATACTCTCAAGCCGAGTTATTAATTGAATCAT
CCATGCGAAGTGGGCCTTTAGAGGCAACGCCTCCCATTGAACAAGTTGCCTC
GTTACTTGATATGAGAATAACATCGCCTGGTAAAAGATCTTTGCTTCAAATGG
GATTTCCAATTTATTCCCTAACTAATCACTTAAGTACCTTGTTGGATAAAGGT
TGGACTGTTATAGTTATCGATGAATTAGTCACTGGTAAATCCGGGCCAAAAC
AACGTGCAGTATCTCAAGTTTATTCTCCTTGTTGTAATTTAGAGGACTGTTCG
GAATTATCCTATGTGTTATCAATTTATTTTTCTCAAGATGACTTATTAGGTATT
ACTTTATTTTCAGCCATGAATGGGCATAGTATAATGTTTCCTGTCTCTTGGGC
GGACAGAGACAAAGTCGCCCGGTTATTAATCAGCTATCGTATTAGAGAAATA
GTAATTTGGGCAAATTTAGAGGTTGGCTCAGAGATTTTAATTAATAAAATATA
TAATTTATTAATTGGCTGGAATTTATTCCCTTCTGAACCCAATATGCTATTTCT
TAGTATTGGTTCCTTTATTAGGCTGGGTAGAAACCAAATTGCTCCGTATGAAA
TAGTATAAGAAGTAAAGTAGGTCATTGTTGGCCGAAGTGCAAT

Pterogorgia_citrina_FK4

CCAGCTTATCCAAATTGGTAAGTTCTATGAACTTTGGCATGAGCCTGA
TACTCCTAGTAGGCAACAAGTATACTCTCAAGCCGAGTTATTAATTGAATCAT
CCATGCGAAGTGGGCCTTTAGAGGCAACGCCTCCCATTGAACAAGTTGCCTC
GTTACTTGATATGAGAATAACATCGCCTGGTAAAAGATCTTTGCTTCAAATGG
GATTTCCAATTTATTCCCTAACTAATCACTTAAGTACCTTGTTGGATAAAGGT
TGGACTGTTATAGTTATCGATGAATTAGTCACTGGTAAATCCGGGCCAAAAC
AACGTGCAGTATCTCAAGTTTATTCTCCTTGTTGTAATTTAGAGGACTGTTCG
GAATTATCCTATGTGTTATCAATTTATTTTTCTCAAGATGACTTATTAGGTATT
ACTTTATTTTCAGCCATGAATGGGCATAGTATAATGTTTCCTGTCTCTTGGGC

GGACAGAGACAAAGTCGCCCGGTTATTAATCAGCTATCGTATTAGAGAAATA
GTAATTTGGGCAAATTTAGAGGTTGGCTCAGAGATTTTAATTAATAAAATATA
TAATTTATTAATTGGCTGGAATTTATTCCCTTCTGAACCCAATATGCTATTTCT
TAGTATTGGTTCCTTTATTAGGCTGGGTAGAAACCAAATTGCTCCGTATGAAA
TAGTATAAGAAGTAAAGTAGGTCATTGTTGGCCGAAGTGCAAT

Pterogorgia_citrina_FK5

CCAGCTTATCCAAATTGGTAAGTTCTATGAACTTTGGCATGAGCCTGA
TACTCCTAGTAGGCAACAAGTATACTCTCAAGCCGAGTTATTAATTGAATCAT
CCATGCGAAGTGGGCCTTTAGAGGCAACGCCTCCCATTGAACAAGTTGCCTC
GTTACTTGATATGAGAATAACATCGCCTGGTAAAAGATCTTTGCTTCAAATGG
GATTTCCAATTTATTCCCTAACTAATCACTTAAGTACCTTGTTGGATAAAGGT
TGGACTGTTATAGTTATCGATGAATTAGTCACTGGTAAATCCGGGCCAAAAC
AACGTGCAGTATCTCAAGTTTATTCTCCTTGTTGTAATTTAGAGGACTGTTCG
GAATTATCCTATGTGTTATCAATTTATTTTTCTCAAGATGACTTATTAGGTATT
ACTTTATTTTCAGCCATGAATGGGCATAGTATAATGTTTCCTGTCTCTTGGGC
GGACAGAGACAAAGTCGCCCGGTTATTAATCAGCTATCGTATTAGAGAAATA
GTAATTTGGGCAAATTTAGAGGTTGGCTCAGAGATTTTAATTAATAAAATATA
TAATTTATTAATTGGCTGGAATTTATTCCCTTCTGAACCCAATATGCTATTTCT
TAGTATTGGTTCCTTTATTAGGCTGGGTAGAAACCAAATTGCTCCGTATGAAA
TAGTATAAGAAGTAAAGTAGGTCATTGTTGGCCGAAGTGCAAT

Pterogorgia_citrina_FK6

CCAGCTTATCCAAATTGGTAAGTTCTATGAACTTTGGCATGAGCCTGA
TACTCCTAGTAGGCAACAAGTATACTCTCAAGCCGAGTTATTAATTGAATCAT
CCATGCGAAGTGGGCCTTTAGAGGCAACGCCTCCCATTGAACAAGTTGCCTC
GTTACTTGATATGAGAATAACATCGCCTGGTAAAAGATCTTTGCTTCAAATGG
GATTTCCAATTTATTCCCTAACTAATCACTTAAGTACCTTGTTGGATAAAGGT
TGGACTGTTATAGTTATCGATGAATTAGTCACTGGTAAATCCGGGCCAAAAC
AACGTGCAGTATCTCAAGTTTATTCTCCTTGTTGTAATTTAGAGGACTGTTCG
GAATTATCCTATGTGTTATCAATTTATTTTTCTCAAGATGACTTATTAGGTATT
ACTTTATTTTCAGCCATGAATGGGCATAGTATAATGTTTCCTGTCTCTTGGGC
GGACAGAGACAAAGTCGCCCGGTTATTAATCAGCTATCGTATTAGAGAAATA
GTAATTTGGGCAAATTTAGAGGTTGGCTCAGAGATTTTAATTAATAAAATATA
TAATTTATTAATTGGCTGGAATTTATTCCCTTCTGAACCCAATATGCTATTTCT
TAGTATTGGTTCCTTTATTAGGCTGGGTAGAAACCAAATTGCTCCGTATGAAA
TAGTATAAGAAGTAAAGTAGGTCATTGTTGGCCGAAGTGCAAT

Pterogorgia_citrina_SB722

CCAGCTTATCCAAATTGGTAAGTTCTATGAACTTTGGCATGAGCCTGA
TACTCCTAGTAGGCAACAAGTATACTCTCAAGCCGAGTTATTAATTGAATCAT
CCATGCGAAGTGGGCCTTTAGAGGCAACGCCTCCCATTGAACAAGTTGCCTC
GTTACTTGATATGAGAATAACATCGCCTGGTAAAAGATCTTTGCTTCAAATGG
GATTTCCAATTTATTCCCTAACTAATCACTTAAGTACCTTGTTGGATAAAGGT
TGGACTGTTATAGTTATCGATGAATTAGTCACTGGTAAATCCGGGCCAAAAC
AACGTGCAGTATCTCAAGTTTATTCTCCTTGTTGTAATTTAGAGGACTGTTCG
GAATTATCCTATGTGTTATCAATTTATTTTTCTCAAGATGACTTATTAGGTATT
ACTTTATTTTCAGCCATGAATGGGCATAGTATAATGTTTCCTGTCTCTTGGGC
GGACAGAGACAAAGTCGCCCGGTTATTAATCAGCTATCGTATTAGAGAAATA

GTAATTTGGGCAAATTTAGAGGTTGGCTCAGAGATTTTAATTAATAAAATATA
TAATTTATTAATTGGCTGGAATTTATTCCCTTCTGAACCCAATATGCTATTTCT
TAGTATTGGTTCCTTTATTAGGCTGGGTAGAAACCAAATTGCTCCGTATGAAA
TAGTATAAGAAGTAAAGTAGGTCATTGTTGGCCGAAGTGCAAT

Pterogorgia_citrina_SB723

CCAGCTTATCCAAATTGGTAAGTTCTATGAACTTTGGCATGAGCCTGA
TACTCCTAGTAGGCAACAAGTATACTCTCAAGCCGAGTTATTAATTGAATCAT
CCATGCGAAGTGGGCCTTTAGAGGCAACGCCTCCCATTGAACAAGTTGCCTC
GTTACTTGATATGAGAATAACATCGCCTGGTAAAAGATCTTTGCTTCAAATGG
GATTTCCAATTTATTCCCTAACTAATCACTTAAGTACCTTGTTGGATAAAGGT
TGGACTGTTATAGTTATCGATGAATTAGTCACTGGTAGATCCGGGCCAAAAC
AACGTGCAGTATCTCAAGTTTATTCTCCTTGTTGTAATTTAGAGGACTGTTCG
GAATTATCCTATGTGTTATCAATTTATTTTTCTCAAGATGACTTATTAGGTATT
ACTTTATTTTCAGCCATGAATGGGCATAGTATAATGTTTCCTGTCTCTTGGGC
GGACAGAGACAAAGTCGCCCGGTTATTAATCAGCTATCGTATTAGAGAAATA
GTAATTTGGGCAAATTTAGAGGTTGGCCCAGAGATTTTAATTAATAAAATAT
ATAATTTATTAATTGGCTGGAATTTATTCCCTTCTGAACCCAATATGCTATTTC
TTAGTATTGGTTCCTTTATTAGGCTGGGTAGAAACCAAATTGCTCCGTATGAA
ATAGTATAAGAAGTAAAGTAGGTCATTGTTGGCCGAAGTGCAAT

Pterogorgia_citrina_SB724

CCAGCTTATCCAAATTGGTAAGTTCTATGAACTTTGGCATGAGCCTGA
TACTCCTAGTAGGCAACAAGTATACTCTCAAGCCGAGTTATTAATTGAATCAT
CCATGCGAAGTGGGCCTTTAGAGGCAACGCCTCCCATTGAACAAGTTGCCTC
GTTACTTGATATGAGAATAACATCGCCTGGTAAAAGATCTTTGCTTCAAATGG
GATTTCCAATTTATTCCCTAACTAATCACTTAAGTACCTTGTTGGATAAAGGT
TGGACTGTTATAGTTATCGATGAATTAGTCACTGGTAAATCCGGGCCAAAAC
AACGTGCAGTATCTCAAGTTTATTCTCCTTGTTGTAATTTAGAGGACTGTTCG
GAATTATCCTATGTGTTATCAATTTATTTTTCTCAAGATGACTTATTAGGTATT
ACTTTATTTTCAGCCATGAATGGGCATAGTATAATGTTTCCTGTCTCTTGGGC
GGACAGAGACAAAGTCGCCCGGTTATTAATCAGCTATCGTATTAGAGAAATA
GTAATTTGGGCAAATTTAGAGGTTGGCTCAGAGATTTTAATTAATAAAATATA
TAATTTATTAATTGGCTGGAATTTATTCCCTTCTGAACCCAATATGCTATTTCT
TAGTATTGGTTCCTTTATTAGGCTGGGTAGAAACCAAATTGCTCCGTATGAAA
TAGTATAAGAAGTAAAGTAGGTCATTGTTGGCCGAAGTGCAAT

Pterogorgia_anceps_FK1

TCAGCTTATCCAAATTGGTAAGTTCTATGAACTTTGGCATGAGCCTGAT
ACTCCTAGTAGGCAACAAGTATACTCTCAAACCGAGTTATTAATTGAATCATC
CATGCGAAGTGGGCCTTTAGAGGCAACGCCTCCCATTGAACAAGTTGCTTCG
TTACTTGATATGAGAATAACATCGCCTGGTAAAAGATCTTTGCTTCAAATGGG
ATTTCCAATTTATTCCCTAACTAATCACTTAAGTACCTTGTTGGATAAAGGTT
GGACTGTTATAGTTATCGATGAATTAGTCACTGGTAAATCTGGGCCAAAACA
ACGTGCAGTATCTCAAGTTTATTCTCCTTGTTGTAATTTAGAAGACTGTTCGG
AATTATCCTATGTGTTATCAATTTATTTTTCTCAAGATGACTTATTAGGTATTA
CTTTATTTTCAGCCATGAATGGGCATAGTATAATGTTTCCTGTTTCTTGGGCG
GACAGAGACAAAGTCGCCCGGTTATTAATCAGCTATCGTATTAGAGAAATAG
TAATTTGGGCAAATTTAGAGGTTGGCTCAGAGATTTTAATTAATAAAATATAT

AATTTATTAATTGGCTGGAATTTATTCCCTTCTGAACCCAATGTGCTATTTCTT
AGTATTGATTCCTTTATTAGGCTGGGTAGAAACCAAATTGCTCCGTATGAAAT
AGTATAAGAAGTAAAATAGGTCATTGTTGGCCGAAGTGCAAT

Pterogorgia_anceps_FK2

TCAGCTTATCCAAATTGGTAAGTTCTATGAACTTTGGCATGAGCCTGAT
ACTCCTAGTAGGCAACAAGTATACTCTCAAACCGAGTTATTAATTGAATCATC
CATGCGAAGTGGGCCTTTAGAGGCAACGCCTCCCATTGAACAAGTTGCTTCG
TTACTTGATATGAGAATAACATCGCCTGGTAAAAGATCTTTGCTTCAAATGGG
ATTTCCAATTTATTCCCTAACTAATCACTTAAGTACCTTGTTGGATAAAGGTT
GGACTGTTATAGTTATCGATGAATTAGTCACTGGTAAATCTGGGCCAAAACA
ACGTGCAGTATCTCAAGTTTATTCTCCTTGTTGTAATTTAGAAGACTGTTCGG
AATTATCCTATGTGTTATCAATTTATTTTTCTCAAGATGACTTATTAGGTATTA
CTTTATTTTCAGCCATGAATGGGCATAGTATAATGTTTCCTGTTTCTTGGGCG
GACAGAGACAAAGTCGCCCGGTTATTAATCAGCTATCGTATTAGAGAAATAG
TAATTTGGGCAAATTTAGAGGTTGGCTCAGAGATTTTAATTAATAAAATATAT
AATTTATTAATTGGCTGGAATTTATTCCCTTCTGAACCCAATGTGCTATTTCTT
AGTATTGATTCCTTTATTAGGCTGGGTAGAAACCAAATTGCTCCGTATGAAAT
AGTATAAGAAGTAAAATAGGTCATTGTTGGCCGAAGTGCAAT

Pterogorgia_anceps_FK3

TCAGCTTATCCAAATTGGTAAGTTCTATGAACTTTGGCATGAGCCTGAT
ACTCCTAGTAGGCAACAAGTATACTCTCAAACCGAGTTATTAATTGAATCATC
CATGCGAAGTGGGCCTTTAGAGGCAACGCCTCCCATTGAACAAGTTGCTTCG
TTACTTGATATGAGAATAACATCGCCTGGTAAAAGATCTTTGCTTCAAATGGG
ATTTCCAATTTATTCCCTAACTAATCACTTAAGTACCTTGTTGGATAAAGGTT
GGACTGTTATAGTTATCGATGAATTAGTCACTGGTAAATCTGGGCCAAAACA
ACGTGCAGTATCTCAAGTTTATTCTCCTTGTTGTAATTTAGAAGACTGTTCGG
AATTATCCTATGTGTTATCAATTTATTTTTCTCAAGATGACTTATTAGGTATTA
CTTTATTTTCAGCCATGAATGGGCATAGTATAATGTTTCCTGTTTCTTGGGCG
GACAGAGACAAAGTCGCCCGGTTATTAATCAGCTATCGTATTAGAGAAATAG
TAATTTGGGCAAATTTAGAGGTTGGCTCAGAGATTTTAATTAATAAAATATAT
AATTTATTAATTGGCTGGAATTTATTCCCTTCTGAACCCAATGTGCTATTTCTT
AGTATTGATTCCTTTATTAGGCTGGGTAGAAACCAAATTGCTCCGTATGAAAT
AGTATAAGAAGTAAAATAGGTCATTGTTGGCCGAAGTGCAAT

Pterogorgia_anceps_FK4

TCAGCTTATCCAAATTGGTAAGTTCTATGAACTTTGGCATGAGCCTGAT
ACTCCTAGTAGGCAACAAGTATACTCTCAAACCGAGTTATTAATTGAATCATC
CATGCGAAGTGGGCCTTTAGAGGCAACGCCTCCCATTGAACAAGTTGCTTCG
TTACTTGATATGAGAATAACATCGCCTGGTAAAAGATCTTTGCTTCAAATGGG
ATTTCCAATTTATTCCCTAACTAATCACTTAAGTACCTTGTTGGATAAAGGTT
GGACTGTTATAGTTATCGATGAATTAGTCACTGGTAAATCTGGGCCAAAACA
ACGTGCAGTATCTCAAGTTTATTCTCCTTGTTGTAATTTAGAAGACTGTTCGG
AATTATCCTATGTGTTATCAATTTATTTTTCTCAAGATGACTTATTAGGTATTA
CTTTATTTTCAGCCATGAATGGGCATAGTATAATGTTTCCTGTTTCTTGGGCG
GACAGAGACAAAGTCGCCCGGTTATTAATCAGCTATCGTATTAGAGAAATAG
TAATTTGGGCAAATTTAGAGGTTGGCTCAGAGATTTTAATTAATAAAATATAT
AATTTATTAATTGGCTGGAATTTATTCCCTTCTGAACCCAATGTGCTATTTCTT

AGTATTGATTCCTTTATTAGGCTGGGTAGAAACCAAATTGCTCCGTATGAAAT
AGTATAAGAAGTAAAATAGGTCATTGTTGGCCGAAGTGCAAT

Pterogorgia_anceps_FK5

TCAGCTTATCCAAATTGGTAAGTTCTATGAACTTTGGCATGAGCCTGAT
ACTCCTAGTAGGCAACAAGTATACTCTCAAACCGAGTTATTAATTGAATCATC
CATGCGAAGTGGGCCTTTAGAGGCAACGCCTCCCATTGAACAAGTTGCTTCG
TTACTTGATATGAGAATAACATCGCCTGGTAAAAGATCTTTGCTTCAAATGGG
ATTTCCAATTTATTCCCTAACTAATCACTTAAGTACCTTGTTGGATAAAGGTT
GGACTGTTATAGTTATCGATGAATTAGTCACTGGTAAATCTGGGCCAAAACA
ACGTGCAGTATCTCAAGTTTATTCTCCTTGTTGTAATTTAGAAGACTGTTCGG
AATTATCCTATGTGTTATCAATTTATTTTTCTCAAGATGACTTATTAGGTATTA
CTTTATTTTCAGCCATGAATGGGCATAGTATAATGTTTCCTGTTTCTTGGGCG
GACAGAGACAAAGTCGCCCGGTTATTAATCAGCTATCGTATTAGAGAAATAG
TAATTTGGGCAAATTTAGAGGTTGGCTCAGAGATTTTAATTAATAAAATATAT
AATTTATTAATTGGCTGGAATTTATTCCCTTCTGAACCCAATGTGCTATTTCTT
AGTATTGATTCCTTTATTAGGCTGGGTAGAAACCAAATTGCTCCGTATGAAAT
AGTATAAGAAGTAAAATAGGTCATTGTTGGCCGAAGTGCAAT

Pterogorgia_anceps_FLR

TCAGCTTATCCAAATTGGTAAGTTCTATGAACTTTGGCATGAGCCTGAT
ACTCCTAGTAGGCAACAAGTATACTCTCAAACCGAGTTATTAATTGAATCATC
CATGCGAAGTGGGCCTTTAGAGGCAACGCCTCCCATTGAACAAGTTGCTTCG
TTACTTGATATGAGAATAACATCGCCTGGTAAAAGATCTTTGCTTCAAATGGG
ATTTCCAATTTATTCCCTAACTAATCACTTAAGTACCTTGTTGGATAAAGGTT
GGACTGTTATAGTTATCGATGAATTAGTCACTGGTAAATCTGGGCCAAAACA
ACGTGCAGTATCTCAAGTTTATTCTCCTTGTTGTAATTTAGAAGACTGTTCGG
AATTATCCTATGTGTTATCAATTTATTTTTCTCAAGATGACTTATTAGGTATTA
CTTTATTTTCAGCCATGAATGGGCATAGTATAATGTTTCCTGTTTCTTGGGCG
GACAGAGACAAAGTCGCCCGGTTATTAATCAGCTATCGTATTAGAGAAATAG
TAATTTGGGCAAATTTAGAGGTTGGCTCAGAGATTTTAATTAATAAAATATAT
AATTTATTAATTGGCTGGAATTTATTCCCTTCTGAACCCAATGTGCTATTTCTT
AGTATTGATTCCTTTATTAGGCTGGGTAGAAACCAAATTGCTCCGTATGAAAT
AGTATAAGAAGTAAAATAGGTCATTGTTGGCCGAAGTGCAAT

Pterogorgia_ancepsCF_SB721

TCAGCTTATCCAAATTGGTAAGTTCTATGAACTTTGGCATGAGCCTGAT
ACTCCTAGTAGGCAACAAGTATACTCTCAAACCGAGTTATTAATTGAATCATC
CATGCGAAGTGGGCCTTTAGAGGCAACGCCTCCCATTGAACAAGTTGCTTCG
TTACTTGATATGAGAATAACATCGCCTGGTAAAAGATCTTTGCTTCAAATGGG
ATTTCCAATTTATTCCCTAACTAATCACTTAAGTACCTTGTTGGATAAAGGTT
GGACTGTTATAGTTATCGATGAATTAGTCACTGGTAAATCTGGGCCAAAACA
ACGTGCAGTATCTCAAGTTTATTCTCCTTGTTGTAATTTAGAAGACTGTTCGG
AATTATCCTATGTGTTATCAATTTATTTTTCTCAAGATGACTTATTAGGTATTA
CTTTATTTTCAGCCATGAATGGGCATAGTATAATGTTTCCTGTTTCTTGGGCG
GACAGAGACAAAGTCGCCCGGTTATTAATCAGCTATCGTATTAGAGAAATAG
TAATTTGGGCAAATTTAGAGGTTGGCTCAGAGATTTTAATTAATAAAATATAT
AATTTATTAATTGGCTGGAATTTATTCCCTTCTGAACCCAATGTGCTATTTCTT

AGTATTGATTCCTTTATTAGGCTGGGTAGAAACCAAATTGCTCCGTATGAAAT
AGTATAAGAAGTAAAATAGGTCATTGTTGGCCGAAGTGCAAT

Pterogorgia_guadalupensis_SB725

TCAGCTTATCCAAATTGGTAAGTTCTATGAACTTTGGCATGAGCCTGAT
ACTCCTAGTAGGCAACAAGTATACTCTCAAACCGAGTTATTAATTGAATCATC
CATGCGAAGTGGGCCTTTAGAGGCAACGCCTCCCATTGAACAAGTTGCTTCG
TTACTTGATATGAGAATAACATCGCCTGGTAAAAGATCTTTGCTTCAAATGGG
ATTTCCAATTTATTCCCTAACTAATCACTTAAGTACCTTGTTGGATAAAGGTT
GGACTGTTATAGTTATCGATGAATTAGTCACTGGTAAATCTGGGCCAAAACA
ACGTGCAGTATCTCAAGTTTATTCTCCTTGTTGTAATTTAGAAGACTGTTCGG
AATTATCCTATGTGTTATCAATTTATTTTTCTCAAGATGACTTATTAGGTATTA
CTTTATTTTCAGCCATGAATGGGCATAGTATAATGTTTCCTGTTTCTTGGGCG
GACAGAGACAAAGTCGCCCGGTTATTAATCAGCTATCGTATTAGAGAAATAG
TAATTTGGGCAAATTTAGAGGTTGGCTCAGAGATTTTAATTAATAAAATATAT
AATTTATTAATTGGCTGGAATTTATTCCCTTMTGAACCCAATGTGCTATTTCTT
AGTATTGATTCCTTTATTAGGCTGGGTAGAAACCAAATTGCTCCGTATGAAAT
AGTATAAGAAGTAAAATAGGTCATTGTTGGCCGAAGTGCAAT

Pterogorgia_guadalupensis_SB726

TCAGCTTATCCAAATTGGTAAGTTCTATGAACTTTGGCATGAGCCTGAT
ACTCCTAGTAGGCAACAAGTATACTCTCAAACCGAGTTATTAATTGAATCATC
CATGCGAAGTGGGCCTTTAGAGGCAACGCCTCCCATTGAACAAGTTGCTTCG
TTACTTGATATGAGAATAACATCGCCTGGTAAAAGATCTTTGCTTCAAATGGG
ATTTCCAATTTATTCCCTAACTAATCACTTAAGTACCTTGTTGGATAAAGGTT
GGACTGTTATAGTTATCGATGAATTAGTCACTGGTAAATCTGGGCCAAAACA
ACGTGCAGTATCTCAAGTTTATTCTCCTTGTTGTAATTTAGAAGACTGTTCGG
AATTATCCTATGTGTTATCAATTTATTTTTCTCAAGATGACTTATTAGGTATTA
CTTTATTTTCAGCCATGAATGGGCATAGTATAATGTTTCCTGTTTCTTGGGCG
GACAGAGACAAAGTCGCCCGGTTATTAATCAGCTATCGTATTAGAGAAATAG
TAATTTGGGCAAATTTAGAGGTTGGCTCAGAGATTTTAATTAATAAAATATAT
AATTTATTAATTGGCTGGAATTTATTCCCTTCTGAACCCAATGTGCTATTTCTT
AGTATTGATTCCTTTATTAGGCTGGGTAGAAACCAAATTGCTCCGTATGAAAT
AGTATAAGAAGTAAAATAGGTCATTGTTGGCCGAAGTGCAAT

Pterogorgia_guadalupensis_SB728

TCAGCTTATCCAAATTGGTAAGTTCTATGAACTTTGGCATGAGCCTGAT
ACTCCTAGTAGGCAACAAGTATACTCTCAAACCGAGTTATTAATTGAATCATC
CATGCGAAGTGGGCCTTTAGAGGCAACGCCTCCCATTGAACAAGTTGCTTCG
TTACTTGATATGAGAATAACATCGCCTGGTAAAAGATCTTTGCTTCAAATGGG
ATTTCCAATTTATTCCCTAACTAATCACTTAAGTACCTTGTTGGATAAAGGTT
GGACTGTTATAGTTATCGATGAATTAGTCACTGGTAAATCTGGGCCAAAACA
ACGTGCAGTATCTCAAGTTTATTCTCCTTGTTGTAATTTAGAAGACTGTTCGG
AATTATCCTATGTGTTATCAATTTATTTTTCTCAAGATGACTTATTAGGTATTA
CTTTATTTTCAGCCATGAATGGGCATAGTATAATGTTTCCTGTTTCTTGGGCG
GACAGAGACAAAGTCGCCCGGTTATTAATCAGCTATCGTATTAGAGAAATAG
TAATTTGGGCAAATTTAGAGGTTGGCTCAGAGATTTTAATTAATAAAATATAT
AATTTATTAATTGGCTGGAATTTATTCCCTTCTGAACCCAATGTGCTATTTCTT

AGTATTGATTCCTTTATTAGGCTGGGTAGAAACCAAATTGCTCCGTATGAAAT
AGTATAAGAAGTAAAATAGGTCATTGTTGGCCGAAGTGCAAT

Pterogorgia_guadalupensis_SB729

TCAGCTTATCCAAATTGGTAAGTTCTATGAACTTTGGCATGAGCCTGAT
ACTCCTAGTAGGCAACAAGTATACTCTCAAACCGAGTTATTAATTGAATCATC
CATGCGAAGTGGGCCTTTAGAGGCAACGCCTCCCATTGAACAAGTTGCTTCG
TTACTTGATATGAGAATAACATCGCCTGGTAAAAGATCTTTGCTTCAAATGGG
ATTTCCAATTTATTCCCTAACTAATCACTTAAGTACCTTGTTGGATAAAGGTT
GGACTGTTATAGTTATCGATGAATTAGTCACTGGTAAATCTGGGCCAAAACA
ACGTGCAGTATCTCAAGTTTATTCTCCTTGTTGTAATTTAGAAGACTGTTCGG
AATTATCCTATGTGTTATCAATTTATTTTTCTCAAGATGACTTATTAGGTATTA
CTTTATTTTCAGCCATGAATGGGCATAGTATAATGTTTCCTGTTTCTTGGGCG
GACAGAGACAAAGTCGCCCGGTTATTAATCAGCTATCGTATTAGAGAAATAG
TAATTTGGGCAAATTTAGAGGTTGGCTCAGAGATTTTAATTAATAAAATATAT
AATTTATTAATTGGCTGGAATTTATTCCCTTCTGAACCCAATGTGCTATTTCTT
AGTATTGATTCCTTTATTAGGCTGGGTAGAAACCAAATTGCTCCGTATGAAAT
AGTATAAGAAGTAAAATAGGTCATTGTTGGCCGAAGTGCAAT

Pterogorgia_guadalupensis_SB727_concat

TCAGCTTATCCAAATTGGTAAGTTCTATGAACTTTGGCATGAGCCTGAT
ACTCCTAGTAGGCAACAAGTATACTCTCAAACCGAGTTATTAATTGAATCATC
CATGCGAAGTGGGCCTTTAGAGGCAACGCCTCCCATTGAACAAGTTGCTTCG
TTACTTGATATGAGAATAACATCGCCTGGTAAAAGATCTTTGCTTCAAATGGG
ATTTCCAATTTATTCCCTAACTAATCACTTAAGTACCTTGTTGGATAAAGGTT
GGACTGTTATAGTTATCGATGAATTAGTCACTGGTAAATCTGGGCCAAAACA
ACGTGCAGTATCTCAAGTTTATTCTCCTTGTTGTAATTTAGAAGACTGTTCGG
AATTATCCTATGTGTTATCAATTTATTTTTCTCAAGATGACTTATTAGGTATTA
CTTTATTTTCAGCCATGAATGGGCATAGTATAATGTTTCCTGTTTCTTGGGCG
GACAGAGACAAAGTCGCCCGGTTATTAATCAGCTATCGTATTAGAGAAATAG
TAATTTGGGCAAATTTAGAGGTTGGCTCAGAGATTTTAATTAATAAAATATAT
AATTTATTAATTGGCTGGAATTTATTCCCTTCTGAACCCAATGTGCTATTTCTT
AGTATTGATTCCTTTATTAGGCTGGGTAGAAACCAAATTGCTCCGTATGAAAT
AGTATAAGAAGTAAAATAGGTCATTGTTGGCCGAAGTGCAAT

Gorgonian_ventalina_FK

CCAACTTATTCAGATTGGTAAGTTCTATGAACTTTGGCATGAGCCTGAT
ACTCCTAGTGGGCAACAAGCATACTCTCAAGCCGAGTTATTAGTTGGGCCAT
CCATGCGAAGTGGGCCTTTGGGGGTAACGCCCCCCATAGAACAAATTGCCTC
GTTACTTGATATGAGAATAATATCGCCCGGCAAAAGATCCTTGCTTCAAATG
GGGTTTCCAATTTACTCCCTTACTACTCACCTAAGTACTTTGTTGGATAAAGG
TTGGACTGTTATAGTTATTGATGAATTAGTTACTGGTAAATCTGGGCCAAAAC
AACGGGCGGTATCTCGGGTTTACTCTCCTAGTTGTAATTTAGAGGACTGTTCG
GAATTACCCTATGTGTTATCAATTTATTCCTCTCAAGACGACTTATTAGGTAT
CACTTTATTTTCAGCCATGAATGGGCATAGTATAATGTTTCCTGTCTCTTGAA
CGGACAGAGACAAAGTAGGCCGGTTATTAATCAGTTATCGTATTAGAGAAAT
AGTAATTTGGGTAGACTCGGGGGCTGGCTCAGAGATTTTAATTAATAAAATA
TATAATTTATTAATTGGTTGGAATCTATTCCCCTCTGAGCCCAACGTTCTACTT

CTTAGTATTGATCCCCCTGTTAGGCTGGGTAGAAACCAAATTACTCCGTATGA
AATAGTATAAGAAGTAGTATAGGTCATTGTTGGCCAAAGTGCAAT
Pseudopterogorgia_acerosa_FK
CCAACTTATTCAGATTGGTAAGTTCTATGAACTTTGGCATGAGCCTGAT
ACTCCTAGTGGGCAACAAGCATACTCTCAAGCCGAGTTATTAGTTGGGCCAT
CCATGCGAAGTAGGCCTTTGGAGGTAACGCCCCCCATAGAACAAATTGCCTC
GTTACTTGATATGAGAATAATATCGCCCGGCAAAAGATCCTTGCTTCAAATG
GGGTTTCCAATTTACTCCCTTACTACTCACCTAAGTACTTTGTTGGATAAAGG
TTGGACTGTTATAGTTATTGATGAATTAGTTACTGGTAAATCTGGGCCAAAAC
AACGCGCGGTATCTCAGGTTTACTCTCCTAGTTGTAATTTAGAGGACTGTTCG
GAATTACCCTATGTGTTATCAATTTATTCCTCTCAAGACGACTTATTAGGTATT
ACTTTATTTTCAGCCATGAATGGGCATAGTATAATGTTTCCTGTCTCTTGAAC
GGACAGAGACAAAGTAGGCCGCTTATTAATCAGTTATCGTATTAGAGAAATA
GTAATTTGGGTAGACTCAGGGGCTGGCTCAGAGATTTTAATTAATAAAATAT
ATAATTTATTAATTGGTTGGAATCTATTCCCCTCTGAGCCCAACGTTCTACTTC
TTAGTATTGATACCCTTGTTAGGCTGGGTAGAAACCAAATTACTCCGTATGAA
ATAGTATAAGAAGTAGTATAGGTCATTGTTGGCCAAAGTGCAAT
Plexaura_flexuosa_FR1
CCAGCTTATCCAAATTGGTAAGTTCTATGAACTTTGGCATGAGCCTAA
TACTCCTAGTAGGCAACAAGCATATTTCAAGCCGAGTTATTAGTTGAGCCAT
CCATGCGAAGTAGGCCTTTGGAGGTGGCGCCCCCCATTGAACAAGTTGCCTC
GTTACTTGATATGAGAATAATATCGCCCGGTAAAAGATCCTTGCTTCAAATGG
GGTTTCCAATTTATTCCCTTACTACTCATCTAAGTATCTTGTTGGATAAAGGTT
GGACTGTTATAGTTATCGATGAATTAGTCACTGGTAAATCAGGGCCAAAACA
ACGCGCAGTATCTCAAGTTTATTCTCCTAGTTGTAATTTAGAGGACTGTTCAG
AATTATCCTATGTGTTATCAATTTATTTTTCTCAAGATGATTTATTAGGTATTA
CTTTATTTTCAGCCATGAATGGGCATAGTATCATGTTTCCTGTCTCTTGGACG
GACAGAGACAAAGTAGCCCGGTTATTAATCAGTTATCGTATTAGAGAAATAG
TAATTTGGGTAGACTTAGGAGTTGGCTCAGAGATTTTAACAAATAAAATATA
TAATTTATTAATTGGTTGGAATTTATTCCCTTGTGAACCCAATGAGCTATTTCT
TAGTATTGATCCCCTTGTTAGGCTGGGCAGAAAACAAATTGCTCCATATGAA
ATAATATAAAAAGTAGTATAGGTCATTGTCGGCCAAAGTGCAAT

;
end;

(6) *SRP54*, *mtMutS*, cytochrome b, *IGR4*

#NEXUS
begin taxa;
dimensions ntax=26;
taxlabels
Pterogorgia_guadalupensis_SB729[&description="Concatenation of 2 sequences"]

Pterogorgia_anceps_FLR[&description="Concatenation of 2 sequences"]
Pterogorgia_anceps_FK5[&description="Concatenation of 2 sequences"]
Pterogorgia_guadalupensis_SB726[&description="Concatenation of 2 sequences"]
Pterogorgia_guadalupensis_SB728[&description="Concatenation of 2 sequences"]
Pterogorgia_anceps_FK4[&description="Concatenation of 2 sequences"]
Pterogorgia_ancepsCF_SB721[&description="Concatenation of 2 sequences"]
Pterogorgia_anceps_FK2[&description="Concatenation of 2 sequences"]
Pterogorgia_anceps_FK1[&description="Concatenation of 2 sequences"]
Pterogorgia_anceps_FK3[&description="Concatenation of 2 sequences"]
Pterogorgia_guadalupensis_SB725[&description="Concatenation of 2 sequences"]
Pterogorgia_guadalupensis_SB727[&description="Concatenation of 2 sequences"]
Pterogorgia_citrina_FK6[&description="Concatenation of 2 sequences"]
Pterogorgia_citrina_FK4[&description="Concatenation of 2 sequences"]
Pterogorgia_citrina_SB722[&description="Concatenation of 2 sequences"]
Pterogorgia_citrina_FK3[&description="Concatenation of 2 sequences"]
Pterogorgia_citrina_FK5[&description="Concatenation of 2 sequences"]
Pterogorgia_citrina_FK2[&description="Concatenation of 2 sequences"]
Pterogorgia_sp_SB2[&description="Concatenation of 2 sequences"]
Pterogorgia_citrina_FK1[&description="Concatenation of 2 sequences"]
Pterogorgia_citrina_SB723[&description="Concatenation of 2 sequences"]
Pterogorgia_citrina_SB724[&description="Concatenation of 2 sequences"]
Pterogorgia_sp_SB1[&description="Concatenation of 2 sequences"]
Gorgonia_ventalina_FK[&description="Concatenation of 2 sequences"]
Pseudopterogorgia_acerosa_FK[&description="Concatenation of 2 sequences"]
Plexaura_flexuosa_FR1[&description="Concatenation of 2 sequences"]
;
end;

begin characters;
dimensions nchar=916;
format datatype=dna missing=? gap=-;
matrix
Pterogorgia_guadalupensis_SB729
TGAACTAAAATTAGAAGAAAATGAAGAACTAATAGGAAAATTAAAAC
ATGGTAAGTAAAT-----ACTTGATCGT---------------------TGACAA--------
GTAAASTAGA--GAGATAACTTACAAG------------AATTACATTATTGATTTTA-
TTAGGACAATTTACGCTAAGATCAGCTTATCCAAATTGGTAAGTTCTATGAAC
TTTGGCATGAGCCTGATACTCCTAGTAGGCAACAAGTATACTCTCAAACCGA
GTTATTAATTGAATCATCCATGCGAAGTGGGCCTTTAGAGGCAACGCCTCCCA
TTGAACAAGTTGCTTCGTTACTTGATATGAGAATAACATCGCCTGGTAAAAG
ATCTTTGCTTCAAATGGGATTTCCAATTTATTCCCTAACTAATCACTTAAGTAC
CTTGTTGGATAAAGGTTGGACTGTTATAGTTATCGATGAATTAGTCACTGGTA

AATCTGGGCCAAAACAACGTGCAGTATCTCAAGTTTATTCTCCTTGTTGTAAT
TTAGAAGACTGTTCGGAATTATCCTATGTGTTATCAATTTATTTTTCTCAAGAT
GACTTATTAGGTATTACTTTATTTTCAGCCATGAATGGGCATAGTATAATGTT
TCCTGTTTCTTGGGCGGACAGAGACAAAGTCGCCCGGTTATTAATCAGCTATC
GTATTAGAGAAATAGTAATTTGGGCAAATTTAGAGGTTGGCTCAGAGATTTT
AATTAATAAAATATATAATTTATTAATTGGCTGGAATTTATTCCCTTCTGAAC
CCAATGTGCTATTTCTTAGTATTGATTCCTTTATTAGGCTGGGTAGAAACCAA
ATTGCTCCGTATGAAATAGTATAAGAAGTAAAATAGGTCATTGTTGGCCGAA
GTGCAAT

Pterogorgia_anceps_FLR

TGAACTAAAATTAGAAGAAAATGAAGAACTAATAGGAAAATTAAAAC
ATGGTAAGTAAAT-----ACTTGATCGT---------------------TGACAA--------
GTAAAGTAGA--GAGATAACTTACAAG------------AATTACATTATTGATTTTA-
TTAGGACAATTTACGCTAAGATCAGCTTATCCAAATTGGTAAGTTCTATGAAC
TTTGGCATGAGCCTGATACTCCTAGTAGGCAACAAGTATACTCTCAAACCGA
GTTATTAATTGAATCATCCATGCGAAGTGGGCCTTTAGAGGCAACGCCTCCCA
TTGAACAAGTTGCTTCGTTACTTGATATGAGAATAACATCGCCTGGTAAAAG
ATCTTTGCTTCAAATGGGATTTCCAATTTATTCCCTAACTAATCACTTAAGTAC
CTTGTTGGATAAAGGTTGGACTGTTATAGTTATCGATGAATTAGTCACTGGTA
AATCTGGGCCAAAACAACGTGCAGTATCTCAAGTTTATTCTCCTTGTTGTAAT
TTAGAAGACTGTTCGGAATTATCCTATGTGTTATCAATTTATTTTTCTCAAGAT
GACTTATTAGGTATTACTTTATTTTCAGCCATGAATGGGCATAGTATAATGTT
TCCTGTTTCTTGGGCGGACAGAGACAAAGTCGCCCGGTTATTAATCAGCTATC
GTATTAGAGAAATAGTAATTTGGGCAAATTTAGAGGTTGGCTCAGAGATTTT
AATTAATAAAATATATAATTTATTAATTGGCTGGAATTTATTCCCTTCTGAAC
CCAATGTGCTATTTCTTAGTATTGATTCCTTTATTAGGCTGGGTAGAAACCAA
ATTGCTCCGTATGAAATAGTATAAGAAGTAAAATAGGTCATTGTTGGCCGAA
GTGCAAT

Pterogorgia_anceps_FK5

TGAACTAAAATTAGAAGAAAATGAAGAACTAATAGGAAAATTAAAAC
ATGGTAAGTAAAT-----ACTTGATCGT---------------------TGACAA--------
GTAAAGTAGA--GAGATAACTTACAAG------------AATTACATTATTGATTTTA-
TTAGGACAATTTACGCTAAGATCAGCTTATCCAAATTGGTAAGTTCTATGAAC
TTTGGCATGAGCCTGATACTCCTAGTAGGCAACAAGTATACTCTCAAACCGA
GTTATTAATTGAATCATCCATGCGAAGTGGGCCTTTAGAGGCAACGCCTCCCA
TTGAACAAGTTGCTTCGTTACTTGATATGAGAATAACATCGCCTGGTAAAAG
ATCTTTGCTTCAAATGGGATTTCCAATTTATTCCCTAACTAATCACTTAAGTAC
CTTGTTGGATAAAGGTTGGACTGTTATAGTTATCGATGAATTAGTCACTGGTA
AATCTGGGCCAAAACAACGTGCAGTATCTCAAGTTTATTCTCCTTGTTGTAAT
TTAGAAGACTGTTCGGAATTATCCTATGTGTTATCAATTTATTTTTCTCAAGAT
GACTTATTAGGTATTACTTTATTTTCAGCCATGAATGGGCATAGTATAATGTT
TCCTGTTTCTTGGGCGGACAGAGACAAAGTCGCCCGGTTATTAATCAGCTATC
GTATTAGAGAAATAGTAATTTGGGCAAATTTAGAGGTTGGCTCAGAGATTTT
AATTAATAAAATATATAATTTATTAATTGGCTGGAATTTATTCCCTTCTGAAC
CCAATGTGCTATTTCTTAGTATTGATTCCTTTATTAGGCTGGGTAGAAACCAA

ATTGCTCCGTATGAAATAGTATAAGAAGTAAAATAGGTCATTGTTGGCCGAA GTGCAAT

Pterogorgia_guadalupensis_SB726

TGAACTAAAATTAGAAGAAAATGAAGAACTAATAGGAAAATTAAAAC ATGGTAAGTAAAT-----ACTTGATCGT---------------------TGACAA-------- GTAAAGTAGA--GAGATAACTTACAAG------------AATTACATTATTGATTTTA- TTAGGACAATTTACGCTAAGATCAGCTTATCCAAATTGGTAAGTTCTATGAAC TTTGGCATGAGCCTGATACTCCTAGTAGGCAACAAGTATACTCTCAAACCGA GTTATTAATTGAATCATCCATGCGAAGTGGCCTTTAGAGGCAACGCCTCCCA TTGAACAAGTTGCTTCGTTACTTGATATGAGAATAACATCGCCTGGTAAAAG ATCTTTGCTTCAAATGGGATTTCCAATTTATTCCCTAACTAATCACTTAAGTAC CTTGTTGGATAAAGGTTGGACTGTTATAGTTATCGATGAATTAGTCACTGGTA AATCTGGGCCAAAACAACGTGCAGTATCTCAAGTTTATTCTCCTTGTTGTAAT TTAGAAGACTGTTCGGAATTATCCTATGTGTTATCAATTTATTTTTCTCAAGAT GACTTATTAGGTATTACTTTATTTTCAGCCATGAATGGGCATAGTATAATGTT TCCTGTTTCTTGGGCGGACAGAGACAAAGTCGCCCGGTTATTAATCAGCTATC GTATTAGAGAAATAGTAATTTGGGCAAATTTAGAGGTTGGCTCAGAGATTTT AATTAATAAAATATATAATTTATTAATTGGCTGGAATTTATTCCCTTCTGAAC CCAATGTGCTATTTCTTAGTATTGATTCCTTTATTAGGCTGGGTAGAAACCAA ATTGCTCCGTATGAAATAGTATAAGAAGTAAAATAGGTCATTGTTGGCCGAA GTGCAAT

Pterogorgia_guadalupensis_SB728

TGAACTAAAATTAGAAGAAAATGAAGAACTAATAGGAAAATTAAAAC ATGGTAAGTAART-----ACTTGATCGT---------------------TGACAA-------- GTAAAGTAGA--GAGATAACTTACAAG------------AATTACATTATTGATTTTA- TTWGGACAATTTACGCTAAGATCAGCTTATCCAAATTGGTAAGTTCTATGAA CTTTGGCATGAGCCTGATACTCCTAGTAGGCAACAAGTATACTCTCAAACCG AGTTATTAATTGAATCATCCATGCGAAGTGGCCTTTAGAGGCAACGCCTCCC ATTGAACAAGTTGCTTCGTTACTTGATATGAGAATAACATCGCCTGGTAAAA GATCTTTGCTTCAAATGGGATTTCCAATTTATTCCCTAACTAATCACTTAAGT ACCTTGTTGGATAAAGGTTGGACTGTTATAGTTATCGATGAATTAGTCACTGG TAAATCTGGGCCAAAACAACGTGCAGTATCTCAAGTTTATTCTCCTTGTTGTA ATTTAGAAGACTGTTCGGAATTATCCTATGTGTTATCAATTTATTTTTCTCAAG ATGACTTATTAGGTATTACTTTATTTTCAGCCATGAATGGGCATAGTATAATG TTTCCTGTTTCTTGGGCGGACAGAGACAAAGTCGCCCGGTTATTAATCAGCTA TCGTATTAGAGAAATAGTAATTTGGGCAAATTTAGAGGTTGGCTCAGAGATT TTAATTAATAAAATATATAATTTATTAATTGGCTGGAATTTATTCCCTTCTGA ACCCAATGTGCTATTTCTTAGTATTGATTCCTTTATTAGGCTGGGTAGAAACC AAATTGCTCCGTATGAAATAGTATAAGAAGTAAAATAGGTCATTGTTGGCCG AAGTGCAAT

Pterogorgia_anceps_FK4

TGAACTAAAATTAGAAGAAAATGAAGAACTAATAGGAAAATTAAAAC ATGGTAAGTAAAT-----ACTTGATCGT---------------------TGACAA-------- GTAAAGTAGA--GRGATAACTTACAAG------------AATTACATYATTGATTTTA- TTAGGACAATTTACGCTAAGATCAGCTTATCCAAATTGGTAAGTTCTATGAAC TTTGGCATGAGCCTGATACTCCTAGTAGGCAACAAGTATACTCTCAAACCGA

GTTATTAATTGAATCATCCATGCGAAGTGGGCCTTTAGAGGCAACGCCTCCCA
TTGAACAAGTTGCTTCGTTACTTGATATGAGAATAACATCGCCTGGTAAAAG
ATCTTTGCTTCAAATGGGATTTCCAATTTATTCCCTAACTAATCACTTAAGTAC
CTTGTTGGATAAAGGTTGGACTGTTATAGTTATCGATGAATTAGTCACTGGTA
AATCTGGGCCAAAACAACGTGCAGTATCTCAAGTTTATTCTCCTTGTTGTAAT
TTAGAAGACTGTTCGGAATTATCCTATGTGTTATCAATTTATTTTTCTCAAGAT
GACTTATTAGGTATTACTTTATTTTCAGCCATGAATGGGCATAGTATAATGTT
TCCTGTTTCTTGGGCGGACAGAGACAAAGTCGCCCGGTTATTAATCAGCTATC
GTATTAGAGAAATAGTAATTTGGGCAAATTTAGAGGTTGGCTCAGAGATTTT
AATTAATAAAATATATAATTTATTAATTGGCTGGAATTTATTCCCTTCTGAAC
CCAATGTGCTATTTCTTAGTATTGATTCCTTTATTAGGCTGGGTAGAAACCAA
ATTGCTCCGTATGAAATAGTATAAGAAGTAAAATAGGTCATTGTTGGCCGAA
GTGCAAT

Pterogorgia_ancepsCF_SB721

TGAACTAAAATTAGAAGAAAATGAAGAACTAATAGGAARATTAAAAC
ATGGTAAGTAAAT-----ACTTGATCGT---------------------TGACAA--------
GTAAAGTAGA--GAGATAACTTACAAG------------AATTASATTATTGATTTTA-
TTAGGACAATTTACGCTAAGATCAGCTTATCCAAATTGGTAAGTTCTATGAAC
TTTGGCATGAGCCTGATACTCCTAGTAGGCAACAAGTATACTCTCAAACCGA
GTTATTAATTGAATCATCCATGCGAAGTGGGCCTTTAGAGGCAACGCCTCCCA
TTGAACAAGTTGCTTCGTTACTTGATATGAGAATAACATCGCCTGGTAAAAG
ATCTTTGCTTCAAATGGGATTTCCAATTTATTCCCTAACTAATCACTTAAGTAC
CTTGTTGGATAAAGGTTGGACTGTTATAGTTATCGATGAATTAGTCACTGGTA
AATCTGGGCCAAAACAACGTGCAGTATCTCAAGTTTATTCTCCTTGTTGTAAT
TTAGAAGACTGTTCGGAATTATCCTATGTGTTATCAATTTATTTTTCTCAAGAT
GACTTATTAGGTATTACTTTATTTTCAGCCATGAATGGGCATAGTATAATGTT
TCCTGTTTCTTGGGCGGACAGAGACAAAGTCGCCCGGTTATTAATCAGCTATC
GTATTAGAGAAATAGTAATTTGGGCAAATTTAGAGGTTGGCTCAGAGATTTT
AATTAATAAAATATATAATTTATTAATTGGCTGGAATTTATTCCCTTCTGAAC
CCAATGTGCTATTTCTTAGTATTGATTCCTTTATTAGGCTGGGTAGAAACCAA
ATTGCTCCGTATGAAATAGTATAAGAAGTAAAATAGGTCATTGTTGGCCGAA
GTGCAAT

Pterogorgia_anceps_FK2

TGAACTAAAATTAGAAGAAAATGAAGAACTAATAGGAARATTAAAAC
ATGGTAAGTAAAT-----ACTTGATCGT---------------------TGACAA--------
GTAAAGTAGA--GAGATAACTYACWAG------------AATTACATTATTGATTTTA-
TTAGGACAATTTACGCTAAGATCAGCTTATCCAAATTGGTAAGTTCTATGAAC
TTTGGCATGAGCCTGATACTCCTAGTAGGCAACAAGTATACTCTCAAACCGA
GTTATTAATTGAATCATCCATGCGAAGTGGGCCTTTAGAGGCAACGCCTCCCA
TTGAACAAGTTGCTTCGTTACTTGATATGAGAATAACATCGCCTGGTAAAAG
ATCTTTGCTTCAAATGGGATTTCCAATTTATTCCCTAACTAATCACTTAAGTAC
CTTGTTGGATAAAGGTTGGACTGTTATAGTTATCGATGAATTAGTCACTGGTA
AATCTGGGCCAAAACAACGTGCAGTATCTCAAGTTTATTCTCCTTGTTGTAAT
TTAGAAGACTGTTCGGAATTATCCTATGTGTTATCAATTTATTTTTCTCAAGAT
GACTTATTAGGTATTACTTTATTTTCAGCCATGAATGGGCATAGTATAATGTT
TCCTGTTTCTTGGGCGGACAGAGACAAAGTCGCCCGGTTATTAATCAGCTATC

GTATTAGAGAAATAGTAATTTGGGCAAATTTAGAGGTTGGCTCAGAGATTTT
AATTAATAAAATATATAATTTATTAATTGGCTGGAATTTATTCCCTTCTGAAC
CCAATGTGCTATTTCTTAGTATTGATTCCTTTATTAGGCTGGGTAGAAACCAA
ATTGCTCCGTATGAAATAGTATAAGAAGTAAAATAGGTCATTGTTGGCCGAA
GTGCAAT

Pterogorgia_anceps_FK1

TGAACTAAAATTAGAAGAAAATGAAGAACTAATAGGAAAATTAAAAC
ATGGTAAGTAAAT-----ACTTGATCGT---------------------TGACAA--------
GTAAAGTAGA--GAGATAACTTACAAG------------AATTAC---ATTGATTTTA-
TTAGGACAATTTACGCTAAGRTCAGCTTATCCAAATTGGTAAGTTCTATGAAC
TTTGGCATGAGCCTGATACTCCTAGTAGGCAACAAGTATACTCTCAAACCGA
GTTATTAATTGAATCATCCATGCGAAGTGGGCCTTTAGAGGCAACGCCTCCCA
TTGAACAAGTTGCTTCGTTACTTGATATGAGAATAACATCGCCTGGTAAAAG
ATCTTTGCTTCAAATGGGATTTCCAATTTATTCCCTAACTAATCACTTAAGTAC
CTTGTTGGATAAAGGTTGGACTGTTATAGTTATCGATGAATTAGTCACTGGTA
AATCTGGGCCAAAACAACGTGCAGTATCTCAAGTTTATTCTCCTTGTTGTAAT
TTAGAAGACTGTTCGGAATTATCCTATGTGTTATCAATTTATTTTTCTCAAGAT
GACTTATTAGGTATTACTTTATTTTCAGCCATGAATGGGCATAGTATAATGTT
TCCTGTTTCTTGGGCGGACAGAGACAAAGTCGCCCGGTTATTAATCAGCTATC
GTATTAGAGAAATAGTAATTTGGGCAAATTTAGAGGTTGGCTCAGAGATTTT
AATTAATAAAATATATAATTTATTAATTGGCTGGAATTTATTCCCTTCTGAAC
CCAATGTGCTATTTCTTAGTATTGATTCCTTTATTAGGCTGGGTAGAAACCAA
ATTGCTCCGTATGAAATAGTATAAGAAGTAAAATAGGTCATTGTTGGCCGAA
GTGCAAT

Pterogorgia_anceps_FK3

TGAACTAAAATTAGAAGAAAATGAAGRACTAATAGGAAAATTAAAAC
ATGGTAAGTAAAT-----ACTTGATCGT---------------------TGACAA--------
GTAAAGTAGA--GAGRTAACTTACAAG------------AATTAC---ATTGATTTTA-
TTAGGACAATTTACGCTAAGATCAGCTTATCCAAATTGGTAAGTTCTATGAAC
TTTGGCATGAGCCTGATACTCCTAGTAGGCAACAAGTATACTCTCAAACCGA
GTTATTAATTGAATCATCCATGCGAAGTGGGCCTTTAGAGGCAACGCCTCCCA
TTGAACAAGTTGCTTCGTTACTTGATATGAGAATAACATCGCCTGGTAAAAG
ATCTTTGCTTCAAATGGGATTTCCAATTTATTCCCTAACTAATCACTTAAGTAC
CTTGTTGGATAAAGGTTGGACTGTTATAGTTATCGATGAATTAGTCACTGGTA
AATCTGGGCCAAAACAACGTGCAGTATCTCAAGTTTATTCTCCTTGTTGTAAT
TTAGAAGACTGTTCGGAATTATCCTATGTGTTATCAATTTATTTTTCTCAAGAT
GACTTATTAGGTATTACTTTATTTTCAGCCATGAATGGGCATAGTATAATGTT
TCCTGTTTCTTGGGCGGACAGAGACAAAGTCGCCCGGTTATTAATCAGCTATC
GTATTAGAGAAATAGTAATTTGGGCAAATTTAGAGGTTGGCTCAGAGATTTT
AATTAATAAAATATATAATTTATTAATTGGCTGGAATTTATTCCCTTCTGAAC
CCAATGTGCTATTTCTTAGTATTGATTCCTTTATTAGGCTGGGTAGAAACCAA
ATTGCTCCGTATGAAATAGTATAAGAAGTAAAATAGGTCATTGTTGGCCGAA
GTGCAAT

Pterogorgia_guadalupensis_SB725

TGAATTAAAATTAGAAGAAAATGAAGAACTAATAGGAAAATTAAAAC
ATGGTAAGTAAAT-----ACTTGATCGT---------------------TGACAA--------

GTAAAATAGAGRGAGATAACTTACAGT------------AATTATATTATTGATTTTA-
TTAGGACAATTTACGCTAAGATCAGCTTATCCAAATTGGTAAGTTCTATGAAC
TTTGGCATGAGCCTGATACTCCTAGTAGGCAACAAGTATACTCTCAAACCGA
GTTATTAATTGAATCATCCATGCGAAGTGGCCTTTAGAGGCAACGCCTCCCA
TTGAACAAGTTGCTTCGTTACTTGATATGAGAATAACATCGCCTGGTAAAAG
ATCTTTGCTTCAAATGGGATTTCCAATTTATTCCCTAACTAATCACTTAAGTAC
CTTGTTGGATAAAGGTTGGACTGTTATAGTTATCGATGAATTAGTCACTGGTA
AATCTGGGCCAAAACAACGTGCAGTATCTCAAGTTTATTCTCCTTGTTGTAAT
TTAGAAGACTGTTCGGAATTATCCTATGTGTTATCAATTTATTTTTCTCAAGAT
GACTTATTAGGTATTACTTTATTTTCAGCCATGAATGGGCATAGTATAATGTT
TCCTGTTTCTTGGGCGGACAGAGACAAAGTCGCCCGGTTATTAATCAGCTATC
GTATTAGAGAAATAGTAATTTGGGCAAATTTAGAGGTTGGCTCAGAGATTTT
AATTAATAAAATATATAATTTATTAATTGGCTGGAATTTATTCCCTTMTGAAC
CCAATGTGCTATTTCTTAGTATTGATTCCTTTATTAGGCTGGGTAGAAACCAA
ATTGCTCCGTATGAAATAGTATAAGAAGTAAAATAGGTCATTGTTGGCCGAA
GTGCAAT

Pterogorgia_guadalupensis_SB727

TGAATTRAAATTAGAAGARAATGAAGAACTAATAGGAAAATTAAAAC
ATGGTAAGTAAAT-----ACTTGATCGT---------------------TGACAA--------
GTAAAATAGAGAGAGATAACTTACAGT------------AATTATATTATTGATTTTA-
TTAGGACAATTTACGCTAAGATCAGCTTATCCAAATTGGTAAGTTCTATGAAC
TTTGGCATGAGCCTGATACTCCTAGTAGGCAACAAGTATACTCTCAAACCGA
GTTATTAATTGAATCATCCATGCGAAGTGGCCTTTAGAGGCAACGCCTCCCA
TTGAACAAGTTGCTTCGTTACTTGATATGAGAATAACATCGCCTGGTAAAAG
ATCTTTGCTTCAAATGGGATTTCCAATTTATTCCCTAACTAATCACTTAAGTAC
CTTGTTGGATAAAGGTTGGACTGTTATAGTTATCGATGAATTAGTCACTGGTA
AATCTGGGCCAAAACAACGTGCAGTATCTCAAGTTTATTCTCCTTGTTGTAAT
TTAGAAGACTGTTCGGAATTATCCTATGTGTTATCAATTTATTTTTCTCAAGAT
GACTTATTAGGTATTACTTTATTTTCAGCCATGAATGGGCATAGTATAATGTT
TCCTGTTTCTTGGGCGGACAGAGACAAAGTCGCCCGGTTATTAATCAGCTATC
GTATTAGAGAAATAGTAATTTGGGCAAATTTAGAGGTTGGCTCAGAGATTTT
AATTAATAAAATATATAATTTATTAATTGGCTGGAATTTATTCCCTTCTGAAC
CCAATGTGCTATTTCTTAGTATTGATTCCTTTATTAGGCTGGGTAGAAACCAA
ATTGCTCCGTATGAAATAGTATAAGAAGTAAAATAGGTCATTGTTGGCCGAA
GTGCAAT

Pterogorgia_citrina_FK6

TGAATTAAAATTAGAAGAAAATGAAGAACTAATAGGAAAATTAAAAC
ATGGTAAGTAAAT-----ACTTGATCGT---------------------TGACAA--------
GTAAAATAGAGAGAGATAACTTACAGT------------AATTATATTATTGATTTTA-
TTAGGACAATTTACGCTAAGACCAGCTTATCCAAATTGGTAAGTTCTATGAAC
TTTGGCATGAGCCTGATACTCCTAGTAGGCAACAAGTATACTCTCAAGCCGA
GTTATTAATTGAATCATCCATGCGAAGTGGCCTTTAGAGGCAACGCCTCCCA
TTGAACAAGTTGCCTCGTTACTTGATATGAGAATAACATCGCCTGGTAAAAG
ATCTTTGCTTCAAATGGGATTTCCAATTTATTCCCTAACTAATCACTTAAGTAC
CTTGTTGGATAAAGGTTGGACTGTTATAGTTATCGATGAATTAGTCACTGGTA
AATCCGGGCCAAAACAACGTGCAGTATCTCAAGTTTATTCTCCTTGTTGTAAT

TTAGAGGACTGTTCGGAATTATCCTATGTGTTATCAATTTATTTTTCTCAAGAT
GACTTATTAGGTATTACTTTATTTTCAGCCATGAATGGGCATAGTATAATGTT
TCCTGTCTCTTGGGCGGACAGAGACAAAGTCGCCCGGTTATTAATCAGCTATC
GTATTAGAGAAATAGTAATTTGGGCAAATTTAGAGGTTGGCTCAGAGATTTT
AATTAATAAAATATATAATTTATTAATTGGCTGGAATTTATTCCCTTCTGAAC
CCAATATGCTATTTCTTAGTATTGGTTCCTTTATTAGGCTGGGTAGAAACCAA
ATTGCTCCGTATGAAATAGTATAAGAAGTAAAGTAGGTCATTGTTGGCCGAA
GTGCAAT

Pterogorgia_citrina_FK4

TGAATTAAAATTAGAAGAAAATGAAGAACTAATAGGAAAATTAAAAC
ATGGTAAGTAAAT-----ACTTGATCGT---------------------TGACAA--------
GTAAAATAGAGAGAGATAACTTACAGT------------AATTATATTATTGATTTTA-
TTAGGACAATTTACGCTAAGACCAGCTTATCCAAATTGGTAAGTTCTATGAAC
TTTGGCATGAGCCTGATACTCCTAGTAGGCAACAAGTATACTCTCAAGCCGA
GTTATTAATTGAATCATCCATGCGAAGTGGGCCTTTAGAGGCAACGCCTCCCA
TTGAACAAGTTGCCTCGTTACTTGATATGAGAATAACATCGCCTGGTAAAAG
ATCTTTGCTTCAAATGGGATTTCCAATTTATTCCCTAACTAATCACTTAAGTAC
CTTGTTGGATAAAGGTTGGACTGTTATAGTTATCGATGAATTAGTCACTGGTA
AATCCGGGCCAAAACAACGTGCAGTATCTCAAGTTTATTCTCCTTGTTGTAAT
TTAGAGGACTGTTCGGAATTATCCTATGTGTTATCAATTTATTTTTCTCAAGAT
GACTTATTAGGTATTACTTTATTTTCAGCCATGAATGGGCATAGTATAATGTT
TCCTGTCTCTTGGGCGGACAGAGACAAAGTCGCCCGGTTATTAATCAGCTATC
GTATTAGAGAAATAGTAATTTGGGCAAATTTAGAGGTTGGCTCAGAGATTTT
AATTAATAAAATATATAATTTATTAATTGGCTGGAATTTATTCCCTTCTGAAC
CCAATATGCTATTTCTTAGTATTGGTTCCTTTATTAGGCTGGGTAGAAACCAA
ATTGCTCCGTATGAAATAGTATAAGAAGTAAAGTAGGTCATTGTTGGCCGAA
GTGCAAT

Pterogorgia_citrina_SB722

TGAATTAAAATTAGAAGAAAATGAAGAACTAATAGGAAAATTAAAAC
ATGGTAAGTAAAT-----ACTTGATCGT---------------------TGACAA--------
GTAAAATAGAGAGAGATAACTTACAGT------------AATTATATTATTGATTTTA-
TTAGGACAATTTACGCTAAGACCAGCTTATCCAAATTGGTAAGTTCTATGAAC
TTTGGCATGAGCCTGATACTCCTAGTAGGCAACAAGTATACTCTCAAGCCGA
GTTATTAATTGAATCATCCATGCGAAGTGGGCCTTTAGAGGCAACGCCTCCCA
TTGAACAAGTTGCCTCGTTACTTGATATGAGAATAACATCGCCTGGTAAAAG
ATCTTTGCTTCAAATGGGATTTCCAATTTATTCCCTAACTAATCACTTAAGTAC
CTTGTTGGATAAAGGTTGGACTGTTATAGTTATCGATGAATTAGTCACTGGTA
AATCCGGGCCAAAACAACGTGCAGTATCTCAAGTTTATTCTCCTTGTTGTAAT
TTAGAGGACTGTTCGGAATTATCCTATGTGTTATCAATTTATTTTTCTCAAGAT
GACTTATTAGGTATTACTTTATTTTCAGCCATGAATGGGCATAGTATAATGTT
TCCTGTCTCTTGGGCGGACAGAGACAAAGTCGCCCGGTTATTAATCAGCTATC
GTATTAGAGAAATAGTAATTTGGGCAAATTTAGAGGTTGGCTCAGAGATTTT
AATTAATAAAATATATAATTTATTAATTGGCTGGAATTTATTCCCTTCTGAAC
CCAATATGCTATTTCTTAGTATTGGTTCCTTTATTAGGCTGGGTAGAAACCAA
ATTGCTCCGTATGAAATAGTATAAGAAGTAAAGTAGGTCATTGTTGGCCGAA
GTGCAAT

Pterogorgia_citrina_FK3

TGAATTAAAATTAGAAGAAAATGAAGAACTAATAGGAAAATTAAAAC
ATGGTAAGTAAAT-----ACTTGATCGT---------------------TGACAA--------
GTAAAATAGAGAGAGATAACTTACAGT------------AATTATATTATTGATTTTA-
TTAGGACAATTTACGCTAAGACCAGCTTATCCAAATTGGTAAGTTCTATGAAC
TTTGGCATGAGCCTGATACTCCTAGTAGGCAACAAGTATACTCTCAAGCCGA
GTTATTAATTGAATCATCCATGCGAAGTGGGCCTTTAGAGGCAACGCCTCCCA
TTGAACAAGTTGCCTCGTTACTTGATATGAGAATAACATCGCCTGGTAAAAG
ATCTTTGCTTCAAATGGGATTTCCAATTTATTCCCTAACTAATCACTTAAGTAC
CTTGTTGGATAAAGGTTGGACTGTTATAGTTATCGATGAATTAGTCACTGGTA
AATCCGGGCCAAAACAACGTGCAGTATCTCAAGTTTATTCTCCTTGTTGTAAT
TTAGAGGACTGTTCGGAATTATCCTATGTGTTATCAATTTATTTTTCTCAAGAT
GACTTATTAGGTATTACTTTATTTTCAGCCATGAATGGGCATAGTATAATGTT
TCCTGTCTCTTGGGCGGACAGAGACAAAGTCGCCCGGTTATTAATCAGCTATC
GTATTAGAGAAATAGTAATTTGGGCAAATTTAGAGGTTGGCTCAGAGATTTT
AATTAATAAAATATATAATTTATTAATTGGCTGGAATTTATTCCCTTCTGAAC
CCAATATGCTATTTCTTAGTATTGGTTCCTTTATTAGGCTGGGTAGAAACCAA
ATTGCTCCGTATGAAATAGTATAAGAAGTAAAGTAGGTCATTGTTGGCCGAA
GTGCAAT

Pterogorgia_citrina_FK5

TGAATTAAAATTAGAAGAAAATGAAGAACTAATAGGAAAATTAAAAC
ATGGTAAGTAAAT-----ACTTGATCGT---------------------TGACAA--------
GTAAAATAGAGAGAGATAACTTACAGT------------AATTATATTATTGATTTTA-
TTAGGACAATTTACGCTAAGACCAGCTTATCCAAATTGGTAAGTTCTATGAAC
TTTGGCATGAGCCTGATACTCCTAGTAGGCAACAAGTATACTCTCAAGCCGA
GTTATTAATTGAATCATCCATGCGAAGTGGGCCTTTAGAGGCAACGCCTCCCA
TTGAACAAGTTGCCTCGTTACTTGATATGAGAATAACATCGCCTGGTAAAAG
ATCTTTGCTTCAAATGGGATTTCCAATTTATTCCCTAACTAATCACTTAAGTAC
CTTGTTGGATAAAGGTTGGACTGTTATAGTTATCGATGAATTAGTCACTGGTA
AATCCGGGCCAAAACAACGTGCAGTATCTCAAGTTTATTCTCCTTGTTGTAAT
TTAGAGGACTGTTCGGAATTATCCTATGTGTTATCAATTTATTTTTCTCAAGAT
GACTTATTAGGTATTACTTTATTTTCAGCCATGAATGGGCATAGTATAATGTT
TCCTGTCTCTTGGGCGGACAGAGACAAAGTCGCCCGGTTATTAATCAGCTATC
GTATTAGAGAAATAGTAATTTGGGCAAATTTAGAGGTTGGCTCAGAGATTTT
AATTAATAAAATATATAATTTATTAATTGGCTGGAATTTATTCCCTTCTGAAC
CCAATATGCTATTTCTTAGTATTGGTTCCTTTATTAGGCTGGGTAGAAACCAA
ATTGCTCCGTATGAAATAGTATAAGAAGTAAAGTAGGTCATTGTTGGCCGAA
GTGCAAT

Pterogorgia_citrina_FK2

TGAATTAAARTTAGAAGAAAATGAAGAACTAATAGGAAAATTAAAAC
ATGGTAAGTAAAT-----ACTTGATCGT---------------------TGACAA--------
GTAAAATAGAGAGAGATAACTTACAGT------------AATTATATTATTGATTTTA-
TTAGGACAATTTACGCTAAGACCAGCTTATCCAAATTGGTAAGTTCTATGAAC
TTTGGCATGAGCCTGATACTCCTAGTAGGCAACAAGTATACTCTCAAGCCGA
GTTATTAATTGAATCATCCATGCGAAGTGGGCCTTTAGAGGCAACGCCTCCCA
TTGAACAAGTTGCCTCGTTACTTGATATGAGAATAACATCGCCTGGTAAAAG

ATCTTTGCTTCAAATGGGATTTCCAATTTATTCCCTAACTAATCACTTAAGTAC
CTTGTTGGATAAAGGTTGGACTGTTATAGTTATCGATGAATTAGTCACTGGTA
AATCCGGGCCAAAACAACGTGCAGTATCTCAAGTTTATTCTCCTTGTTGTAAT
TTAGAGGACTGTTCGGAATTATCCTATGTGTTATCAATTTATTTTTCTCAAGAT
GACTTATTAGGTATTACTTTATTTTCAGCCATGAATGGGCATAGTATAATGTT
TCCTGTCTCTTGGGCGGACAGAGACAAAGTCGCCCGGTTATTAATCAGCTATC
GTATTAGAGAAATAGTAATTTGGGCAAATTTAGAGGTTGGCTCAGAGATTTT
AATTAATAAAATATATAATTTATTAATTGGCTGGAATTTATTCCCTTCTGAAC
CCAATATGCTATTTCTTAGTATTGGTTCCTTTATTAGGCTGGGTAGAAACCAA
ATTGCTCCGTATGAAATAGTATAAGAAGTAAAGTAGGTCATTGTTGGCCGAA
GTGCAAT

Pterogorgia_sp_SB2

TGAATTAAAATTAGAAGAAAATGAAGAACTAATAGGAAAATTAAAAC
ATGGTAAGTAAAT-----ACTTGATCGT---------------------TGACAA--------
GTAAAATAGAGAGAGATAACTTACRGT------------AATTATATWATTGATTTTA-
TTAGGACAATTTACGCTAAGACCAGCTTATCCAAATTGGTAAGTTCTATGAAC
TTTGGCATGAGCCTGATACTCCTAGTAGGCAACAAGTATACTCTCAAGCCGA
GTTATTAATTGAATCATCCATGCGAAGTGGGCCTTTAGAGGCAACGCCTCCCA
TTGAACAAGTTGCCTCGTTACTTGATATGAGAATAACATCGCCTGGTAAAAG
ATCTTTGCTTCAAATGGGATTTCCAATTTATTCCCTAACTAATCACTTAAGTAC
CTTGTTGGATAAAGGTTGGACTGTTATAGTTATCGATGAATTAGTCACTGGTA
AATCCGGGCCAAAACAACGTGCAGTATCTCAAGTTTATTCTCCTTGTTGTAAT
TTAGAGGACTGTTCGGAATTATCCTATGTGTTATCAATTTATTTTTCTCAAGAT
GACTTATTAGGTATTACTTTATTTTCAGCCATGAATGGGCATAGTATAATGTT
TCCTGTCTCTTGGGCGGACAGAGACAAAGTCGCCCGGTTATTAATCAGCTATC
GTATTAGAGAAATAGTAATTTGGGCAAATTTAGAGGTTGGCTCAGAGATTTT
AATTAATAAAATATATAATTTATTAATTGGCTGGAATTTATTCCCTTCTGAAC
CCAATATGCTATTTCTTAGTATTGGTTCCTTTATTAGGCTGGGTAGAAACCAA
ATTGCTCCGTATGAAATAGTATAAGAAGTAAAGTAGGTCATTGTTGGCCGAA
GTGCAAT

Pterogorgia_citrina_FK1

TGAATTAAAATTAGAAGAAAATGAAGAACTAATAGGAAAATTAAAAC
ATGGTAAGTAAAT-----ACTTGATCGT---------------------TGACAA--------
GTAAAATAGARAGAGATAACTTACAGT------------AATTATATTATTGATTTTA-
TTAGGACAATTTACGCTAAGACCAGCTTATCCAAATTGGTAAGTTCTATGAAC
TTTGGCATGAGCCTGATACTCCTAGTAGGCAACAAGTATACTCTCAAGCCGA
GTTATTAATTGAATCATCCATGCGAAGTGGGCCTTTAGAGGCAACGCCTCCCA
TTGAACAAGTTGCCTCGTTACTTGATATGAGAATAACATCGCCTGGTAAAAG
ATCTTTGCTTCAAATGGGATTTCCAATTTATTCCCTAACTAATCACTTAAGTAC
CTTGTTGGATAAAGGTTGGACTGTTATAGTTATCGATGAATTAGTCACTGGTA
AATCCGGGCCAAAACAACGTGCAGTATCTCAAGTTTATTCTCCTTGTTGTAAT
TTAGAGGACTGTTCGGAATTATCCTATGTGTTATCAATTTATTTTTCTCAAGAT
GACTTATTAGGTATTACTTTATTTTCAGCCATGAATGGGCATAGTATAATGTT
TCCTGTCTCTTGGGCGGACAGAGACAAAGTCGCCCGGTTATTAATCAGCTATC
GTATTAGAGAAATAGTAATTTGGGCAAATTTAGAGGTTGGCTCAGAGATTTT
AATTAATAAAATATATAATTTATTAATTGGCTGGAATTTATTCCCTTCTGAAC

CCAATATGCTATTTCTTAGTATTGGTTCCTTTATTAGGCTGGGTAGAAACCAA
ATTGCTCCGTATGAAATAGTATAAGAAGTAAAGTAGGTCATTGTTGGCCGAA
GTGCAAT

Pterogorgia_citrina_SB723

TGAATTAAAATTAGAAGRAAATGARGAACTAATAGGAARATTAAAAC
ATGGTAAGTAAAT-----ACTTGATCGT---------------------TGACAA--------
GTAAAATAGAGAGAGATAACTTACAGT------------AATTATATTATTGATTTTA-
TTAGGACAATTTACGCTAAGRCCAGCTTATCCAAATTGGTAAGTTCTATGAAC
TTTGGCATGAGCCTGATACTCCTAGTAGGCAACAAGTATACTCTCAAGCCGA
GTTATTAATTGAATCATCCATGCGAAGTGGGCCTTTAGAGGCAACGCCTCCCA
TTGAACAAGTTGCCTCGTTACTTGATATGAGAATAACATCGCCTGGTAAAAG
ATCTTTGCTTCAAATGGGATTTCCAATTTATTCCCTAACTAATCACTTAAGTAC
CTTGTTGGATAAAGGTTGGACTGTTATAGTTATCGATGAATTAGTCACTGGTA
GATCCGGGCCAAAACAACGTGCAGTATCTCAAGTTTATTCTCCTTGTTGTAAT
TTAGAGGACTGTTCGGAATTATCCTATGTGTTATCAATTTATTTTTCTCAAGAT
GACTTATTAGGTATTACTTTATTTTCAGCCATGAATGGGCATAGTATAATGTT
TCCTGTCTCTTGGGCGGACAGAGACAAAGTCGCCCGGTTATTAATCAGCTATC
GTATTAGAGAAATAGTAATTTGGGCAAATTTAGAGGTTGGCCCAGAGATTTT
AATTAATAAAATATATAATTTATTAATTGGCTGGAATTTATTCCCTTCTGAAC
CCAATATGCTATTTCTTAGTATTGGTTCCTTTATTAGGCTGGGTAGAAACCAA
ATTGCTCCGTATGAAATAGTATAAGAAGTAAAGTAGGTCATTGTTGGCCGAA
GTGCAAT

Pterogorgia_citrina_SB724

TGAATTAAAATTAGAAGAAAATGAAGAACTAATAGGAAAATTAAAAC
ATGGTAAGTAAAT-----ACTTGATCGT---------------------TGACAA--------
GTAAAATAGAGAGAGATAACTTACAGT------------AATTATATTATTGAYTTTA-
TTAGGACAATTTACGCTAAGACCAGCTTATCCAAATTGGTAAGTTCTATGAAC
TTTGGCATGAGCCTGATACTCCTAGTAGGCAACAAGTATACTCTCAAGCCGA
GTTATTAATTGAATCATCCATGCGAAGTGGGCCTTTAGAGGCAACGCCTCCCA
TTGAACAAGTTGCCTCGTTACTTGATATGAGAATAACATCGCCTGGTAAAAG
ATCTTTGCTTCAAATGGGATTTCCAATTTATTCCCTAACTAATCACTTAAGTAC
CTTGTTGGATAAAGGTTGGACTGTTATAGTTATCGATGAATTAGTCACTGGTA
AATCCGGGCCAAAACAACGTGCAGTATCTCAAGTTTATTCTCCTTGTTGTAAT
TTAGAGGACTGTTCGGAATTATCCTATGTGTTATCAATTTATTTTTCTCAAGAT
GACTTATTAGGTATTACTTTATTTTCAGCCATGAATGGGCATAGTATAATGTT
TCCTGTCTCTTGGGCGGACAGAGACAAAGTCGCCCGGTTATTAATCAGCTATC
GTATTAGAGAAATAGTAATTTGGGCAAATTTAGAGGTTGGCTCAGAGATTTT
AATTAATAAAATATATAATTTATTAATTGGCTGGAATTTATTCCCTTCTGAAC
CCAATATGCTATTTCTTAGTATTGGTTCCTTTATTAGGCTGGGTAGAAACCAA
ATTGCTCCGTATGAAATAGTATAAGAAGTAAAGTAGGTCATTGTTGGCCGAA
GTGCAAT

Pterogorgia_sp_SB1

TGAATTAAAATTAGAAGAAAATGRAGAACTAATAGGAAAATTAAAAC
ATGGTAAGTAAAT-----ACTTGATCGT---------------------TGACAA--------
GTAAAATAGAGAGAGATAACTTACAGT------------AATTATATTATTGATTTWA-
TTAGGACAATTTACGCTAAGACCAGCTTATCCAAATTGGTAAGTTCTATGAAC

TTTGGCATGAGCCTGATACTCCTAGTAGGCAACAAGTATACTCTCAAGCCGA
GTTATTAATTGAATCATCCATGCGAAGTGGGCCTTTAGAGGCAACGCCTCCCA
TTGAACAAGTTGCCTCGTTACTTGATATGAGAATAACATCGCCTGGTAAAAG
ATCTTTGCTTCAAATGGGATTTCCAATTTATTCCCTAACTAATCACTTAAGTAC
CTTGTTGGATAAAGGTTGGACTGTTATAGTTATCGATGAATTAGTCACTGGTA
AATCCGGGCCAAAACAACGTGCAGTATCTCAAGTTTATTCTCCTTGTTGTAAT
TTAGAGGACTGTTCGGAATTATCCTATGTGTTATCAATTTATTTTTCTCAAGAT
GACTTATTAGGTATTACTTTATTTTCAGCCATGAATGGGCATAGTATAATGTT
TCCTGTCTCTTGGGCGGACAGAGACAAAGTCGCCCGGTTATTAATCAGCTATC
GTATTAGAGAAATAGTAATTTGGGCAAATTTAGAGGTTGGCTCAGAGATTTT
AATTAATAAAATATATAATTTATTAATTGGCTGGAATTTATTCCCTTCTGAAC
CCAATATGCTATTTCTTAGTATTGGTTCCTTTATTAGGCTGGGTAGAAACCAA
ATTGCTCCGTATGAAATAGTATAAGAAGTAAAGTAGGTCATTGTTGGCCGAA
GTGCAAT

Gorgonia_ventalina_FK

TGAACTAAAATTGGAAGAAAATGAAGAACTAATAGGAAAATTGAAAC
ATGGTAGGTTTTAATAGATTTGATTTTTTTGCACTGTACRTGTGTACTTGTA
ATAYTTTTGTACTAAAGCCAAATATGCTAGTGATC------------
ATTGACATGTTTGATTTTA-
TTAGGCCAGTTTACTTTGAGGCCAACTTATTCAGATTGGTAAGTTCTATGAAC
TTTGGCATGAGCCTGATACTCCTAGTGGGCAACAAGCATACTCTCAAGCCGA
GTTATTAGTTGGGCCATCCATGCGAAGTGGGCCTTTGGGGGTAACGCCCCCC
ATAGAACAAATTGCCTCGTTACTTGATATGAGAATAATATCGCCCGGCAAAA
GATCCTTGCTTCAAATGGGGTTTCCAATTTACTCCCTTACTACTCACCTAAGT
ACTTTGTTGGATAAAGGTTGGACTGTTATAGTTATTGATGAATTAGTTACTGG
TAAATCTGGGCCAAAACAACGGGCGGTATCTCGGGTTTACTCTCCTAGTTGTA
ATTTAGAGGACTGTTCGGAATTACCCTATGTGTTATCAATTTATTCCTCTCAA
GACGACTTATTAGGTATCACTTTATTTTCAGCCATGAATGGGCATAGTATAAT
GTTTCCTGTCTCTTGAACGGACAGAGACAAAGTAGGCCGGTTATTAATCAGTT
ATCGTATTAGAGAAATAGTAATTTGGGTAGACTCGGGGGCTGGCTCAGAGAT
TTTAATTAATAAAATATATAATTTATTAATTGGTTGGAATCTATTCCCCTCTGA
GCCCAACGTTCTACTTCTTAGTATTGATCCCCCTGTTAGGCTGGGTAGAAACC
AAATTACTCCGTATGAAATAGTATAAGAAGTAGTATAGGTCATTGTTGGCCA
AAGTGCAAT

Pseudopterogorgia_acerosa_FK

TGARCTAAAATTGGAAGAGAATGAAGAACTAATAGGAAAATTAAAAC
ATGGTAGGTTTTT-----
ATTTGATTTTCTTTGCACTGTACATGTGTAATTGTAATATTTTTTNTAMGAAA
GCCTAGCATGTTATTAACC------------
ATTGACGTGTTTGATTTTATTTAGGCCAGTTTACTTTGAGACCAACTTATTCAG
ATTGGTAAGTTCTATGAACTTTGGCATGAGCCTGATACTCCTAGTGGGCAACA
AGCATACTCTCAAGCCGAGTTATTAGTTGGGCCATCCATGCGAAGTAGGCCTT
TGGAGGTAACGCCCCCCATAGAACAAATTGCCTCGTTACTTGATATGAGAAT
AATATCGCCCGGCAAAAGATCCTTGCTTCAAATGGGGTTTCCAATTTACTCCC
TTACTACTCACCTAAGTACTTTGTTGGATAAAGGTTGGACTGTTATAGTTATT
GATGAATTAGTTACTGGTAAATCTGGGCCAAAACAACGCGCGGTATCTCAGG

TTTACTCTCCTAGTTGTAATTTAGAGGACTGTTCGGAATTACCCTATGTGTTAT
CAATTTATTCCTCTCAAGACGACTTATTAGGTATTACTTTATTTTCAGCCATGA
ATGGGCATAGTATAATGTTTCCTGTCTCTTGAACGGACAGAGACAAAGTAGG
CCGCTTATTAATCAGTTATCGTATTAGAGAAATAGTAATTTGGGTAGACTCAG
GGGCTGGCTCAGAGATTTTAATTAATAAAATATATAATTTATTAATTGGTTGG
AATCTATTCCCCTCTGAGCCCAACGTTCTACTTCTTAGTATTGATACCCTTGTT
AGGCTGGGTAGAAACCAAATTACTCCGTATGAAATAGTATAAGAAGTAGTAT
AGGTCATTGTTGGCCAAAGTGCAAT

Plexaura_flexuosa_FR1

CGAACTTRAATTAGAAGAAAACGAAGAATTAATAGGAAAATTAAAAC
ATGGTAGGTGAAT----
GGTGTGATTGTATAGAGAATAGGATTCTCATTTAGCAATG------
AAATGACAAAGGAGCATTCTGARCACTGAATGGCAAGTGAATGACGTTGCGT
CTTTTTTTCAGGTCAGTTCACACTGAGACCAGCTTATCCAAATTGGTAAGTTC
TATGAACTTTGGCATGAGCCTAATACTCCTAGTAGGCAACAAGCATATTTTCA
AGCCGAGTTATTAGTTGAGCCATCCATGCGAAGTAGGCCTTTGGAGGTGGCG
CCCCCCATTGAACAAGTTGCCTCGTTACTTGATATGAGAATAATATCGCCCGG
TAAAAGATCCTTGCTTCAAATGGGGTTTCCAATTTATTCCCTTACTACTCATCT
AAGTATCTTGTTGGATAAAGGTTGGACTGTTATAGTTATCGATGAATTAGTCA
CTGGTAAATCAGGGCCAAAACAACGCGCAGTATCTCAAGTTTATTCTCCTAGT
TGTAATTTAGAGGACTGTTCAGAATTATCCTATGTGTTATCAATTTATTTTTCT
CAAGATGATTTATTAGGTATTACTTTATTTTCAGCCATGAATGGGCATAGTAT
CATGTTTCCTGTCTCTTGGACGGACAGAGACAAAGTAGCCCGGTTATTAATCA
GTTATCGTATTAGAGAAATAGTAATTTGGGTAGACTTAGGAGTTGGCTCAGA
GATTTTAACAAATAAAATATATAATTTATTAATTGGTTGGAATTTATTCCCTT
GTGAACCCAATGAGCTATTTCTTAGTATTGATCCCCTTGTTAGGCTGGGCAGA
AAACAAATTGCTCCATATGAAATAATATAAAAAGTAGTATAGGTCATTGTCG
GCCAAAGTGCAAT

;
end;
