# Supplementary material for: Molecular and Morphological Species Boundaries in the Gorgonian Octocoral Genus Pterogorgia (Octocorallia: Gorgoniidae)
Source: PLoS One. 2015 Jul 21;10(7):e0133517. doi: 10.1371/journal.pone.0133517 (PMC4510298; doi:10.1371/journal.pone.0133517)
Supplement: S2 Fig — Phylograms generated from ITS2 sequences for (a) unique clones found for each individual and (b) consensus sequences for each individual using Bayesian Inference (BI), maximum likelihood (ML), and maximum parsimony (MP). Node support for BI, ML and MP is shown from left to right. A method with node support <50 contains “—". In (a) clones are coded by a “c” (clone) followed by the number of the clone relative to the total unique clones found for that individual. (PDF) [file pone.0133517.s002.pdf]

Figure S1 Figure. ITS2 phylogenies. Phylograms generated from ITS2 sequences for (a) unique clones found for each individual and (b) consensus sequences for each individual using Bayesian likelihood (BL), maximum likelihood (ML), and maximum parsimony (MP). Node support for BL, ML and MP is shown from left to right. Clades with node support <50 for all three phylogenetic reconstruction methods contain "--". In (a) clones are coded by a "c" (clone) followed by the number of the clone relative to the total unique clones found for that individual.

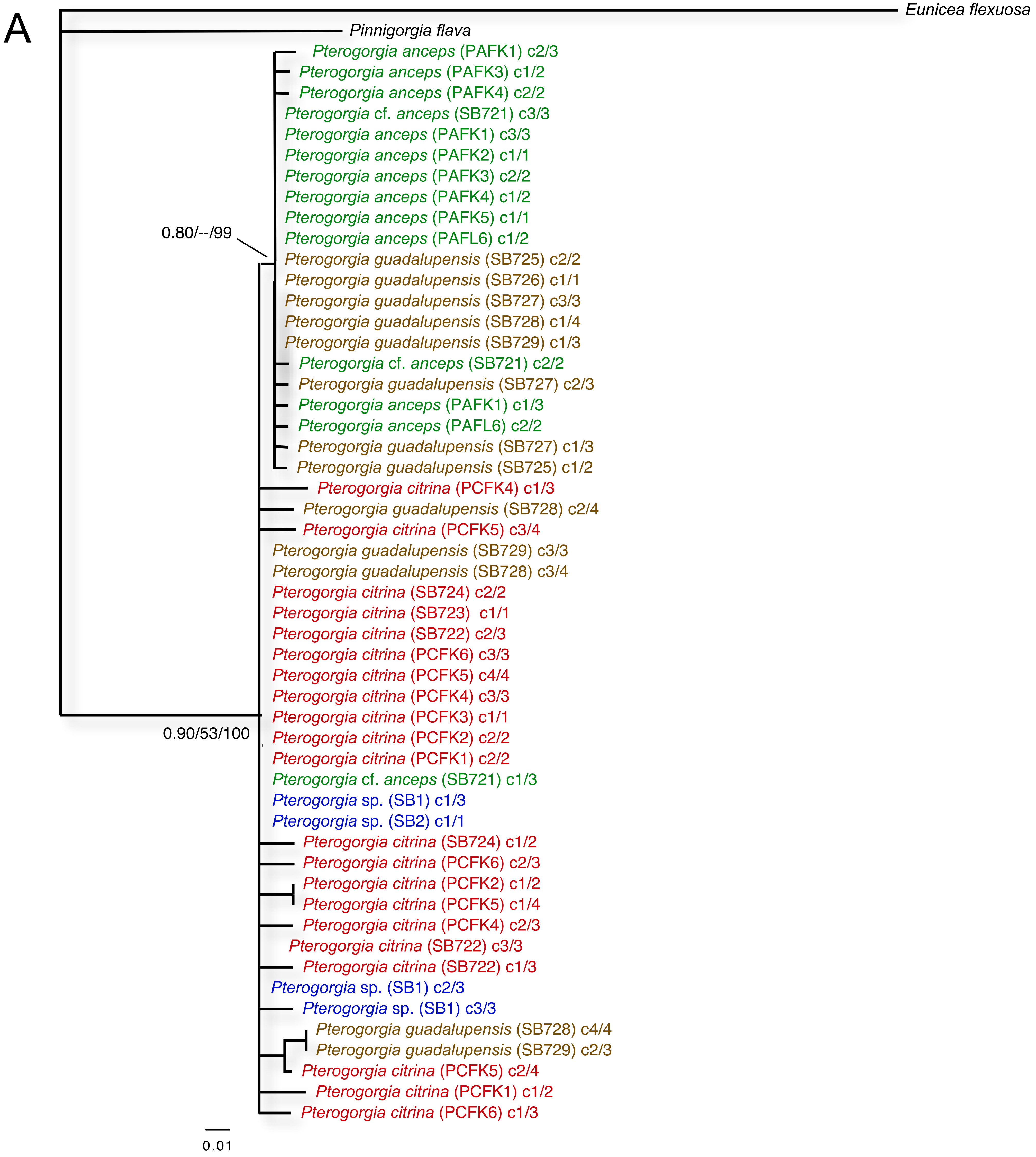

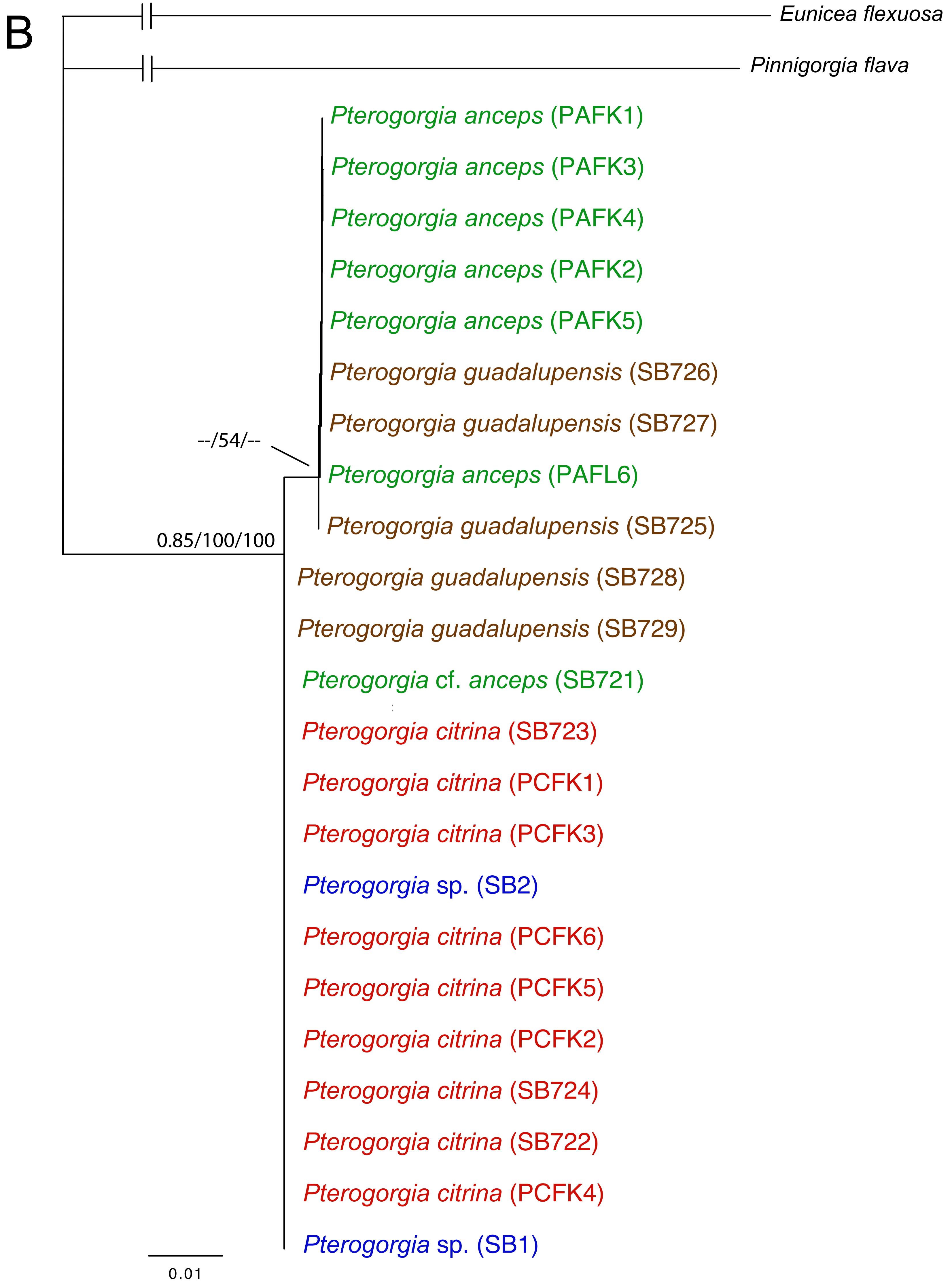

B
Eunicea flexuosa
Pinnigorgia flava
Pterogorgia anceps (PAFK1)
Pterogorgia anceps (PAFK3)
Pterogorgia anceps (PAFK4)
Pterogorgia anceps (PAFK2)
Pterogorgia anceps (PAFK5)
Pterogorgia guadalupensis (SB726)
Pterogorgia guadalupensis (SB727)
--/54/--
Pterogorgia anceps (PAFL6)
Pterogorgia guadalupensis (SB725)
0.85/100/100
Pterogorgia guadalupensis (SB728)
Pterogorgia guadalupensis (SB729)
Pterogorgia cf. anceps (SB721)
Pterogorgia citrina (SB723)
Pterogorgia citrina (PCFK1)
Pterogorgia citrina (PCFK3)
Pterogorgia sp. (SB2)
Pterogorgia citrina (PCFK6)
Pterogorgia citrina (PCFK5)
Pterogorgia citrina (PCFK2)
Pterogorgia citrina (SB724)
Pterogorgia citrina (SB722)
Pterogorgia citrina (PCFK4)
Pterogorgia sp. (SB1)
0.01
