# Supplementary material for: Molecular and Morphological Species Boundaries in the Gorgonian Octocoral Genus Pterogorgia (Octocorallia: Gorgoniidae)
Source: PLoS One. 2015 Jul 21;10(7):e0133517. doi: 10.1371/journal.pone.0133517 (PMC4510298; doi:10.1371/journal.pone.0133517)
Supplement: S1 Table — The number of clones found for each individual for SRP54. (PDF) [file pone.0133517.s003.pdf]

S1 Table. *SRP54* clones. The number of clones found for each individual for *SRP54**.

			Sequence variant 1	Sequence variant 2	Sequence variant 3	Sequenc variant 4
Pterogoriga	citrina	PCFK1	n=6 (75%)	n=2 (25%)		
Pterogoriga	citrina	PCFK2	n=6 (85.7%)	n=1 (14.3%)		
Pterogoriga	citrina	PCFK3	n=8 (100%)			
Pterogoriga	citrina	PCFK4	n=8 (100%)			
Pterogoriga	citrina	PCFK5	n=8 (100%)			
Pterogoriga	citrina	PCFK6	n=8 (100%)			
Pterogoriga	citrina	USNM1122722, SB722	n=8 (100%)			
Pterogoriga	citrina	USNM1122723, SB723	n=6 (75%)	n=1 (12.5%)	n=1 (12.5%)	
Pterogoriga	citrina	USNM1122724, SB724	n=7 (87.5%)	n=1 (12.5%)		
Pterogorgia	sp.	USNM1122730, SB2	n=4 (57.1%)	n=2 (28.6%)	n=1 (14.3%)	
Pterogorgia	sp.	SB1	n=4 (57.1%)	n=2 (28.6%)	n=1 (14.3%)	
Pterogorgia	anceps	PAFK1	n=7 (87.5%)	n=1 (12.5%)		
Pterogorgia	anceps	PAFK2	n=4 (57.1%)	n=1 (14.3%)	n=1 (14.3%)	n=1 (14.3%)
Pterogorgia	anceps	PAFK3	n=5 (62.5%)	n=1 (12.5%)	n=1 (12.5%)	n=1 (12.5%)
Pterogorgia	anceps	PAFK4	n=7 (87.5%)	n=1 (12.5%)		
Pterogorgia	anceps	PAFK5	n=8 (100%)			
Pterogorgia	anceps	PAFL6	n=8 (100%)			
Pterogorgia	cf. anceps	USNM1122721, SB721	n=5 (71.4%)	n=1 (14.3%)	n=1 (14.3%)	
Pterogorgia	guadalupensis	USNM1122725, SB725	n=7 (87.5%)	n=1 (12.5%)		
Pterogorgia	guadalupensis	USNM1122726, SB726	n=7 (100%)			
Pterogorgia	guadalupensis	USNM1122727, SB727	n=6 (75%)	n=1 (12.5%)	n=1 (12.5%)	
Pterogorgia	guadalupensis	USNM1122728, SB728	n=6 (75%)	n=1 (12.5%)	n=1 (12.5%)	
Pterogorgia	guadalupensis	USNM1122729, SB729	n=6 (75%)	n=2 (25%)		

* Total amount of clones sequenced for each individual was 7 or 8 (depending on sequencing success).
